# Twisted Tin‐Chloride Perovskite Single‐Crystal Heterostructures

**DOI:** 10.1002/anie.202520140

**Published:** 2025-12-19

**Authors:** Jamie L. Cleron, Chih‐Yi Chen, Feng Pan, Santanu Saha, Frederick P. Marlton, Robert M. Stolz, Jiayi Li, Jennifer A. Dionne, Fang Liu, Marina R. Filip, Hemamala I. Karunadasa

**Affiliations:** ^1^ Department of Chemistry Stanford University Stanford CA 94305 USA; ^2^ Department of Materials Science and Engineering Stanford University Stanford CA 94305 USA; ^3^ Department of Physics University of Oxford Clarendon Laboratory Parks Road Oxford OXI 3PU UK; ^4^ Institut de Recherche sur les Ceramiques (IRCER) UMR CNRS 7315‐Université de Limoges 12 Rue Atlantis Limoges 87068 France; ^5^ School of Mathematical and Physical Sciences Faculty of Science University of Technology Sydney Sydney NSW 2007 Australia; ^6^ Stanford Institute for Materials and Energy Sciences (SIMES) SLAC National Accelerator Laboratory Menlo Park CA 94025 USA

**Keywords:** Band structure, Interfacial strain, Perovskite phases, Polarized spectroscopy, Twisted heterostructure

## Abstract

Self‐assembly affords simpler synthetic routes to heterostructures compared with manual layer‐by‐layer stacking, yet controlling interlayer twist angles in a bulk solid remains an outstanding challenge. We report two new single‐crystal heterostructures: (Sn_2_Cl_2_)(CYS)_2_SnCl_4_ (CYS = ^+^NH_3_(CH_2_)_2_S^–^; **Sn_CYS**) and (Sn_2_Cl_2_)(SeCYS)_2_SnCl_4_ (SeCYS = ^+^NH_3_(CH_2_)_2_Se^–^; **Sn_SeCYS**) synthesized in solution, with alternating perovskite and intergrowth layers. Notably, compared to the recently reported lead analog, (Pb_2_Cl_2_)(CYS)_2_PbCl_4_ (**Pb_CYS**), the tin heterostructures feature a twist between the perovskite and intergrowth layers. We trace this twist to local distortions at the Sn centers, which change the interfacial lattice‐matching requirements compared to those of the Pb analog. Electronic band structure calculations show that the striking differences in the relative energies of perovskite‐ and intergrowth‐derived bands in **Sn_CYS** and **Pb_CYS** arise from structural and not compositional differences. The structural anisotropy of **Sn_CYS** is also reflected in a large in‐plane photoluminescence linear anisotropy ratio. Interfacial strain further affords differential incorporation of Pb into the perovskite and intergrowth layers of the Sn heterostructures, resulting in redshifted optical absorption onsets. Thus, we posit that local structural distortions may be exploited to manipulate the twist angle and interfacial strain in bulk heterostructures, providing a new handle for tuning the band alignments of bulk quantum‐well electronic structures.

## Graphical abstract

Replacing lead with tin in a single‐crystal halide perovskite heterostructure drives a twist between the perovskite (gray) and intergrowth (blue) layers. The accompanying structural distortions and interfacial strain change the calculated orbital composition of the band edges and enable high in‐plane optical anisotropy in the Sn analog. (VBT = valence band top; CBB = conduction band bottom).

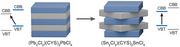

## Introduction

The realization of emergent phenomena at the interface where two monolayers meet has created intense interest in heterostructures formed one layer at a time.^[^ ^1^, ^2^, ^3^, ^4^, ^5^, ^6^ ^]^ Such heterostructures afford a tunable electronic landscape dictated by the choice of monolayers.^[^ ^3^, ^4^ ^]^ Recently, the demonstration that a twist between the layers can drive dramatic electronic transformations has provided an intriguing new avenue for manipulating electronic structure.^[^ ^7^ ^]^ Despite the impressive advances in this field, however, most studies revolve around monolayers that are readily peeled from van der Waals solids with hexagonal symmetry. Further, exfoliating and restacking monolayers requires expert manipulation and specialized equipment. Vapor deposition or multi‐step processes afford more scalable heterostructure films, but exerting precise control over interfaces remains a challenge.^[^ ^8^, ^9^, ^10^, ^11^, ^12^ ^]^

Bulk materials that self‐assemble in solution or in the solid state have considerably cheaper and simpler syntheses. Although there are examples of bulk heterostructures, including naturally occurring minerals,^[^ ^13^, ^14^, ^15^ ^]^ they are limited in number and most require high‐temperature syntheses. For example, misfit layered compounds, comprising alternating rock‐salt and hexagonal or trigonal layers have been synthesized through solid‐state or deposition techniques^[^ ^16^, ^17^ ^]^ and solid‐state syntheses afford cuprate superconductors, consisting of copper‐oxide sheets separated by inorganic charge‐reservoir layers.^[^ ^18^, ^19^, ^20^ ^]^ Furthermore, creating a twist between layers in a self‐assembling solid remains a great challenge. In nanoscale and mesoscale inorganic structures, twists between layers have been created through solution‐state self‐assembly,^[^ ^21^ ^]^ epitaxial growth and solid‐state transformation,^[^ ^22^ ^]^ intercalation,^[^ ^23^ ^]^ chemical vapor deposition,^[^ ^24^, ^25^ ^]^ screw dislocation,^[^ ^26^, ^27^ ^]^ and liquid‐phase exfoliation and restacking.^[^ ^28^ ^]^ However, twist angles are difficult to generate and predict in bulk heterostructures, and to intentionally twist the layers of a self‐assembled solid with infinitely repeating layers we must understand the driving forces behind the displacement.

We have previously demonstrated that bifunctional organic molecules can direct the assembly of layered heterostructures in solution, where the two different termini of the molecule template two different inorganic layers, placing various inorganic intergrowth layers between 2D halide perovskite layers.^[^ ^29^, ^30^, ^31^ ^]^ These materials offer a promising, scalable platform for potentially realizing emergent properties at the repeating interfaces of a bulk crystalline solid. Of particular interest is the (Pb_2_Cl_2_)(CYS)_2_PbCl_4_ heterostructure (CYS = ^+^H_3_N(CH_2_)_2_S^‒^; **Pb_CYS**), where a bridging halide connects the perovskite and intergrowth layers, enabling enhanced interlayer electronic interactions. The calculated band structure of **Pb_CYS** shows a valence‐band top (VBT) comprising primarily intergrowth states and a conduction‐band bottom (CBB) formed primarily of perovskite states. Indeed, the calculated lowest‐energy optical exciton shows some delocalization across perovskite and intergrowth layers. Since the Pb frontier orbitals have a substantial presence at the band edges, we hypothesized that replacing the Pb 6s and 6p orbitals with the higher‐energy Sn 5s and 5p orbitals (due to the lesser relativistic contraction of the Sn core orbitals)^[^ ^32^ ^]^ will significantly modulate the band‐edge orbital composition.

Herein, we report the (Sn_2_Cl_2_)(*RCh*)_2_SnCl_4_ heterostructures (**Sn_RCh**; *RCh* = CYS or SeCYS (^+^H_3_N(CH_2_)_2_Se^‒^). We find that replacing *M* = Pb^2+^ with Sn^2+^ in the (*M*_2_Cl_2_)(CYS)_2_ *M*Cl_4_ heterostructure induces a twist between the perovskite and intergrowth layers and an increase in anisotropic interfacial lattice strain, which we attribute to the stereochemically active 5s^2^ lone pair in Sn.^[^ ^33^ ^]^ Calculations indicate that the band extrema of **Sn_RCh** consist largely of contributions from a highly strained intergrowth layer. We demonstrate how the optical absorption onsets of the **Sn_RCh** family respond to the chalcogen identity and to Pb alloying at the Sn site. We further investigate the structural origins of the optical anisotropy in **Sn_RCh**, with an in‐plane photoluminescence linear anisotropy ratio of up to 9.5 for **Sn_CYS**.

## Results and Discussion

### Synthesis and Structure

Dissolving SnCl_2_ and cysteamine (CYS) or selenocysteamine hydrochloride (SeCYS·HCl) in hot 5.3 M aqueous NaCl and allowing the solutions to cool afforded single crystals of **Sn_RCh** (see the Supporting Information for detailed procedures). The crystals form as blades with the flat face parallel to the intralayer planes (Figure S1). Combustion analysis for C, H, and N agreed with the formula (Sn_2_Cl_2_)(*RCh*)_2_SnCl_4_ for both heterostructures, and powder X‐ray diffraction (PXRD) patterns agreed well with simulated patterns from single‐crystal X‐ray diffraction (SC‐XRD) structures^[^ ^34^ ^]^ (Figure S2, S3). Pulverized crystals were used for all powder measurements.

The SC‐XRD structures indicated that **Sn_CYS** and **Sn_SeCYS** are isostructural, crystallizing in the *C*2*/c* space group. Similar to **Pb_CYS** (*Pnma* space group; Figure S4),^[^ ^29^, ^35^ ^]^ the **Sn_RCh** heterostructures consist of perovskite layers of corner‐sharing, distorted SnCl_6_ octahedra and intergrowth layers that resemble slices excised from the (001) crystallographic plane of the 3D SnCl_2_ crystal structure^[^ ^36^ ^]^ (Figure S5). The perovskite and intergrowth layers are directly connected by a bridging chloride, resulting in a 3D metal‐chloride network.

**Figure 1:**
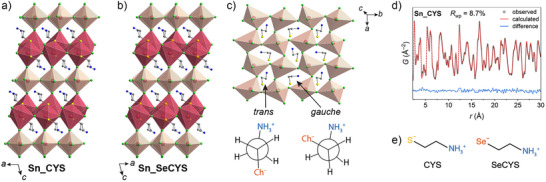
Single‐crystal X‐ray diffraction structures of a) (Sn_2_Cl_2_)(CYS)_2_SnCl_4_ (**Sn_CYS**) and b) (Sn_2_Cl_2_)(SeCYS)_2_SnCl_4_ (**Sn_SeCYS**), in the *P* $\bar{1}$ space group. The perovskite and intergrowth polyhedra are shaded in cream and dark red, respectively. Atom colors: Se: orange; S: yellow; Cl: green; N: blue; C: grey. Only the majority‐occupancy positions of disordered atoms are shown, and hydrogen atoms are omitted. c) Ordering of the *gauche* and *trans* conformations of CYS in **Sn_CYS**. d) Pair distribution function (PDF) fitting of **Sn_CYS** at 100 K. e) Schematics of the cysteamine (CYS) and selenocysteamine (SeCYS) ligands.

In the *C*2*/c* SC‐XRD solutions, all Sn and Cl positions in the perovskite layer, as well as C, N, and H atoms, were equally disordered across two sites. To resolve this disorder and visualize distortions of the perovskite octahedra, we obtained SC‐XRD solutions for **Sn_RCh** in the triclinic space group *P* $\bar{1}$ (Figure 1, Tables S1, S2). At 100 K, the *P* $\bar{1}$ solutions for **Sn_RCh** showed disorder, in the perovskite layer and in C, N, and H atoms, between two superimposed sublattices, one with 76%–80% occupancy and the other with 20%–24% occupancy. An “ordered model” was constructed by using only the majority‐occupation sites for the disordered atoms. This model revealed highly distorted and tilted SnCl_6_ octahedra with three long Sn–Cl bonds (2.9–3.8 Å) and three short Sn–Cl bonds (2.5–2.6 Å), indicative of stereochemical expression of the Sn^2+^ 5s^2^ lone pair.^[^ ^33^, ^37^, ^38^, ^39^ ^]^ To test if these local distortions were reasonable, and not an artifact of disorder modeling, we synthesized a new 2D tin‐chloride perovskite with a sulfur‐containing A‐site cation, (T4YMA)_2_SnCl_4_ (**Sn_control**; T4YMA = thian‐4‐ylmethanammonium), which showed similar octahedral tilting as in the perovskite layers of the *P* $\bar{1}$ **Sn_RCh** ordered model (Figure S6). The calculated octahedral distortion parameters Δ_oct_^[^ ^40^ ^]^ and σ^2^_oct_^[^ ^41^ ^]^ (which quantify local distortions) for the ordered models of **Sn_RCh, Sn_control**, and previously reported 2D^[^ ^42^ ^]^ and 3D tin chloride perovskites^[^ ^43^ ^]^ are roughly comparable (Table S6), suggesting that any significant changes in optical properties in **Sn_RCh,** compared to those of typical tin‐chloride perovskites, primarily arise from the intergrowth layers.

The ordered *P* $\bar{1}$ models of **Sn_RCh** show the CYS and SeCYS ligands alternating between *gauche* and *trans* conformations, with dihedral angles of ca. 70° and 170°, respectively (Figure 1c). The conformation is correlated with the different shapes of the cavities between perovskite octahedra due to tilting, where the *gauche* conformation occupies longer and narrower cavities and the *trans* conformation occupies shorter and wider cavities. Raman spectra (Figure S7) corroborated the presence and ratios of both rotamers in **Sn_RCh**.

We collected high‐energy X‐ray scattering data suitable for pair distribution function analysis (PDF) using powders of **Sn_CYS** to further corroborate our ordered model from SC‐XRD. PDF analysis is sensitive to the local structure, providing additional structural information that may become averaged when analyzing Bragg peaks alone. Refinements were conducted against the PDF data using the ordered *P* $\bar{1}$ model. The PDF at 100 K was fit to reduce peak broadening from thermal motion at room temperature. Due to the many atoms and degrees of freedom in the *P* $\bar{1}$ space group, restraints were implemented to avoid overfitting and generating unphysical models (see the Supporting Information). We obtained a good fit to the PDF (Figure 1d), providing further evidence for the structural distortions observed in our ordered models. The CIF for the structure obtained from PDF fitting is provided as Supporting Information.

### A Twist between the Layers

**Figure 2:**
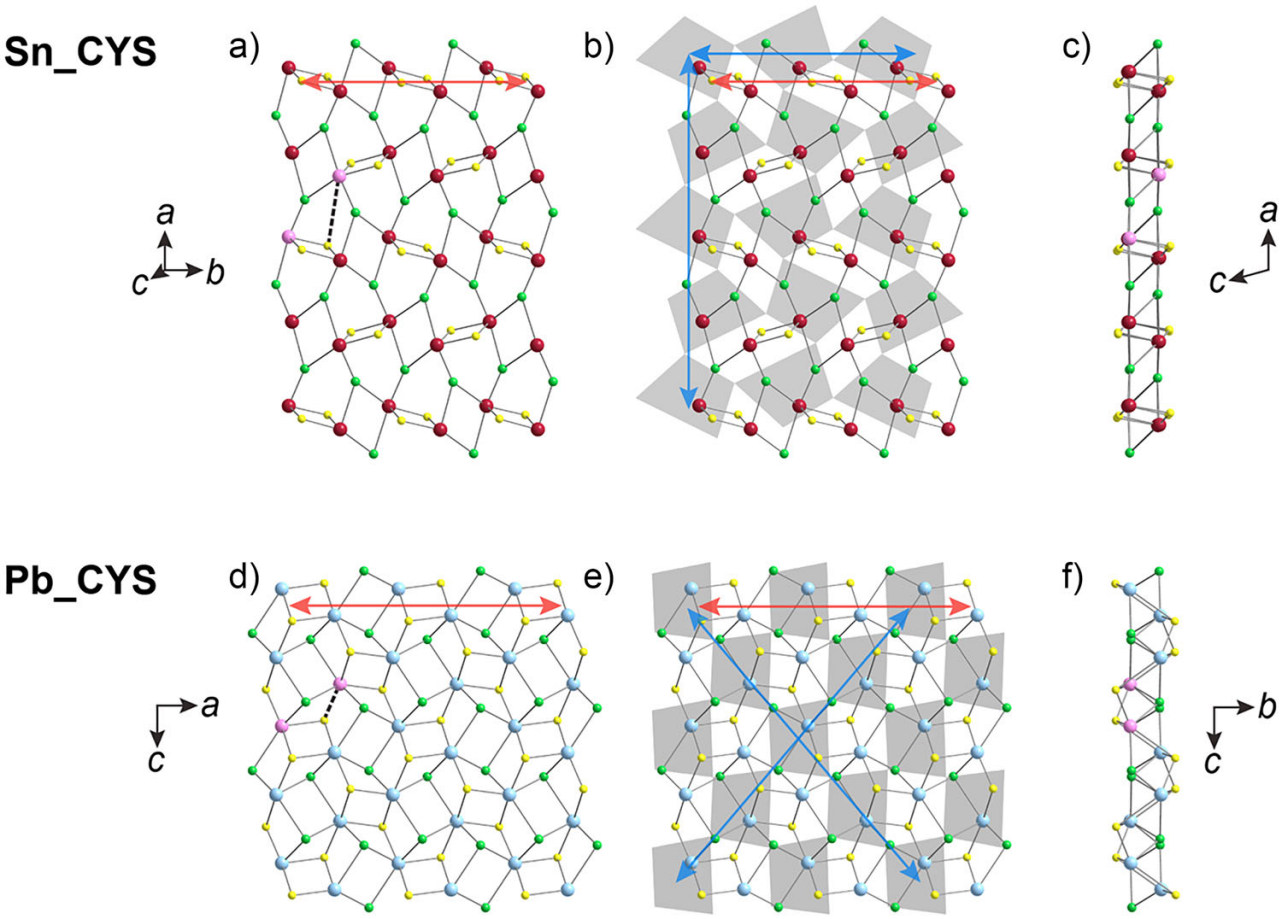
The difference in interlayer twist angle for **Sn_CYS** (top; *P* $\bar{1}$ model) compared with that of **Pb_CYS** (bottom). a) The intergrowth layer of **Sn_CYS**, with the propagation direction of the chains of [Sn_2_Cl_2_] and [Sn_2_S_2_] dimers shown with a red arrow (Figure S5) and the broken Sn–S bond (> 5 Å), which links the dimer chains in the **Pb_CYS** structure, shown by a black dashed line. Pink atoms indicate a pair of metal atoms in adjacent dimer chains. b) The intergrowth layer of **Sn_CYS**, with a projection of the perovskite layer superimposed. The directions of corner‐sharing connectivity of the perovskite octahedra are shown with blue arrows. c) A side‐on view of the intergrowth layer shown in (A), note that the metal atoms highlighted in pink are on opposite sides of the layer. d) The intergrowth layer of **Pb_CYS**, with the propagation direction of the chains of [Pb_2_Cl_2_] and [Pb_2_S_2_] dimers shown with a red arrow and the Pb–S (2.7 Å) bond linking the dimer chains shown by a black dotted line. Pink atoms indicate a pair of metal atoms in adjacent dimer chains, which are equivalent to the pink atoms in (A). e) The intergrowth layer of **Pb_CYS**, with a projection of the perovskite layer superimposed. The directions of corner‐sharing connectivity of the perovskite octahedra are shown with blue arrows. f) A side‐on view of the intergrowth layer shown in (D), note that the metal atoms highlighted in pink are on the same side of the layer. Atom colors: Pb: turquoise; Sn: maroon; Cl: green; S: yellow.

Despite the structural similarities between **Sn_RCh** and **Pb_CYS**, they differ notably in the twist angle between the perovskite and intergrowth layers (Figure 2). The 2D intergrowth layer can be viewed as connected 1D chains of alternating [*M*_2_Cl_2_] and [*M*_2_ *Ch*_2_] dimers (*M* = Sn, Pb; red arrows in Figure 2; Figure S5). In **Sn_RCh**, these chains are aligned along a direction of corner‐sharing connectivity of the [SnCl_6_] perovskite octahedra (blue arrows in Figure 2b). In **Pb_CYS**, however, the 1D chains in the intergrowth layer (red arrow) are at a ca. 50° angle to the direction of corner‐sharing connectivity of the [PbCl_6_] octahedra (blue arrows in Figure 2e). We attribute this difference in twist angle to the much larger *n*s^2^ lone‐pair expression for Sn^2+^ compared to that of Pb^2+[^ ^33^ ^]^ causing octahedral distortions in the perovskite layer that allow the Sn–Sn distances along the *b*‐axis (6.2–6.3 Å) to be significantly longer than those along the *a*‐axis (5.4 Å). We propose that this distortion of the perovskite layer allows for lattice‐matching with the intergrowth layer in a different orientation than for **Pb_CYS**. Furthermore, to connect to the perovskite layers above and below, the **Sn_RCh** intergrowth layer distorts compared to the **Pb_CYS** intergrowth layer. The **Pb_CYS** intergrowth layer is a closer match to a slice cut from the (001) crystallographic plane of PbCl_2_; however, in the intergrowth layer of **Sn_RCh**, every other chain of corner‐sharing dimers “flips”, inverting the positions of the metal atoms to opposite sides of the intergrowth layer (Figure 2c,f). This distortion breaks the metal–S bond that links the dimer chains in **Pb_CYS** (2.7 Å in **Pb_CYS**; > 5 Å in **Sn_RCh**; dashed black line in Figure 2a,d).

We expected the difference in twist angle between **Sn_RCh** and **Pb_CYS** to change the strain induced by interfacial lattice‐matching requirements. Following our previously reported method,^[^ ^29^ ^]^ lattice strain in each layer of **Sn_RCh** (*P* $\bar{1}$ model) was estimated by calculating strain tensors for the perovskite and intergrowth sublattices relative to similar 3D parent lattices (Figure S10; Table S7, S8).^[^ ^36^, ^43^ ^]^ Compared to the SnCl_2_ lattice, the intergrowth layers show significant expansion along the *a*‐axis (16%–17%) and contraction along the *b*‐axis (19%–20%). Compared with (CH_3_NH_3_)SnCl_3_, the perovskite layers of **Sn_RCh** show a corresponding contraction along the *a*‐axis (5%) and expansion along the *b*‐axis (9%–10%), indicating that both sublattices exhibit large anisotropic in‐plane distortions to accommodate each other at the shared interface. Considering the similarity of Sn–Cl, Sn–S, and Sn–Se bond lengths in the heterostructure and in SnCl_2_,^[^ ^36^ ^]^ SnS, and SnSe,^[^ ^44^ ^]^ respectively, we attribute the distortion in the intergrowth layer, relative to SnCl_2_, to strain induced by interfacial lattice matching, rather than the replacement of Cl with S or Se. The perovskite layer of **Pb_CYS** shows a 7% contraction and a 9% expansion along the in‐plane *a‐* and *c*‐axes, respectively, compared to (CH_3_NH_3_)PbCl_3_ (Table S9).^[^ ^45^ ^]^ By contrast, the intergrowth layer of **Pb_CYS** shows small, nearly isotropic strain, with only a 2.3%–2.4% contraction along each in‐plane axis compared to the PbCl_2_ lattice.^[^ ^46^ ^]^ The larger strain and anisotropy in **Sn_RCh** can be explained by the significant lone‐pair expression and elongated bond lengths in the perovskite sublattice, along with the lattice‐matching requirements at the interlayer interface. The different twist angle in **Pb_CYS** mitigates this strain by providing a different lattice‐matching requirement. Similar to strain‐induced bandgap tuning in 2D semiconductors^[^ ^47^, ^48^, ^49^ ^]^ we hypothesized that these distortions induced by the heterostructure framework will substantially affect their electronic structures.

### Calculated Electronic Structure

**Figure 3:**
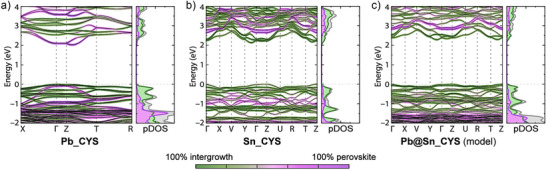
Calculated electronic band structures (left) and projected density of states (pDOS, right) of a) **Pb_CYS**, b) **Sn_CYS**, and c) the model compound: **Pb@Sn_CYS,** in which all Sn atoms are replaced with Pb in the structure of **Sn_CYS**. The color gradient shows the relative perovskite (purple) and intergrowth (green) contributions to the electronic band structures. The band structures are calculated along the high symmetry paths: X(0.5,0,0) – Γ(0,0,0) – Z(0,0.5,0) – T(0,0.5,0.5) – R(0.5,0.5,0.5) for **Pb_CYS**, and Γ(0,0,0) – X(0.5,0,0) – V(0.5,0.5,0) – Y(0,0.5,0) – Γ(0,0,0) – Z(0,0,0.5) – U(0.5,0,0.5) – R(0.5,0.5,0.5) – T(0,0.5,0.5) – Z(0,0,0.5) for **Sn_CYS** and **Pb@Sn_CYS**; Figure S16. Here, the inorganic sheets are parallel to the *ab* plane. The energies of the valence band maxima have been arbitrarily set to 0 eV. The Γ→Z direction corresponds to the interlayer stacking direction. Color scheme for the pDOS: perovskite: purple; intergrowth: green; total: grey.

We calculated the electronic band structures for simplified model structures of **Sn_CYS** and **Sn_SeCYS**, constructed from 100‐K SC‐XRD structures, (Figures 3, S11; note that the 100‐K and 300‐K SC‐XRD structures of **Sn_RCh** are very similar) using density functional theory (DFT)^[^ ^50^, ^51^ ^]^ within the Perdew–Burke–Ernzerhof (PBE)^[^ ^52^ ^]^ parametrization of the generalized gradient approximation, and including spin–orbit coupling, as implemented in the Quantum Espresso code^[^ ^53^ ^]^ (see the Supporting Information for details). The high‐symmetry path for the **Sn_RCh** band structures was chosen using the Bilbao Crystallographic Server get_kvec utility.^[^ ^54^ ^]^ The band structures include contributions from the perovskite and intergrowth layers to each electronic state (Figures 3, S11, S12), with the bridging Cl split equally between the layers. The contributions of individual elements to the valence band top (VBT) and conduction band bottom (CBB) at the Γ and Z points are listed in Table S11 (Figures S13–S15). For **Sn_CYS**, we predict an indirect bandgap of 2.11 eV (Z → between Γ and Y), with a direct gap at the Z point of 2.23 eV (Figure 3b). At the Z point, the band extrema comprise mostly intergrowth states. The CBB at the Z point is dominated by states from the intergrowth layer (1% perovskite, 99% intergrowth), predominantly from Sn contributions (76% intergrowth Sn states), whereas the VBT comprises a mix of perovskite and intergrowth states (28% perovskite, 72% intergrowth). This band composition is strikingly different from that of **Pb_CYS,** computed for a model structure with relaxed atomic positions, starting from a room‐temperature SC‐XRD structure (Figure 3a).^[^ ^29^ ^]^

The CBB at the Γ point of **Pb_CYS** has 92% perovskite states, whereas the CBB at the Z point of **Sn_CYS** has 99% intergrowth states. We attribute this difference in CBB composition to the large dispersion of the intergrowth bands in **Sn_CYS**, created by the areal contraction required of the intergrowth layer to share an interface with the perovskite lattice. Conversely, the distortions and tilting in the Sn perovskite layer reduce orbital overlap and thus dispersion of the perovskite bands, raising the empty perovskite states above the CBB.

The VBT of **Pb_CYS** at the Γ point is dominated by intergrowth states (94%), largely from sulfur contributions (65% S, 17% intergrowth Pb). The VBT of **Sn_CYS** at the Z point has less S character (23% S states) and more metal character (46% Sn states in total). We attribute this difference in orbital composition to the higher energy of the Sn 5s states, compared to the relativistically stabilized Pb 6s states. In the band structure of **Sn_SeCYS**, we see increased chalcogen contribution to the VBT (43% Se at the Z point) compared to that of **Sn_CYS** (23% S) due to the higher energy of the Se states compared with the S states (Figure S11). The Se states reduce the perovskite contributions to the VBT (only 5% perovskite) and narrow the direct bandgap (1.97 eV at the Z point).

To understand whether these striking differences in band structure for **Sn_CYS** and **Pb_CYS** were due to changes in structure or composition, we calculated the band structure for the hypothetical **Pb@Sn_CYS**, where we replaced all Sn atoms with Pb in the ordered model of **Sn_CYS** (Figure 3c). We find that simply replacing Sn with Pb does not reproduce the band alignment of **Pb_CYS**. Although **Pb_CYS** shows dominant perovskite and intergrowth states in the CBB and VBT, respectively, **Pb@Sn_CYS** shows a similar band orbital composition as in **Sn_CYS**, with both the VBT and the CBB dominated by intergrowth states (Table S11). Thus, the differences in octahedral distortion, interlayer twist angle, and lattice strain, appear to change the calculated bandgap, from a mostly interlayer transition in **Pb_CYS** to a mixed inter‐ and intra‐layer transition in **Sn_CYS**.

### Optical Properties

**Figure 4:**
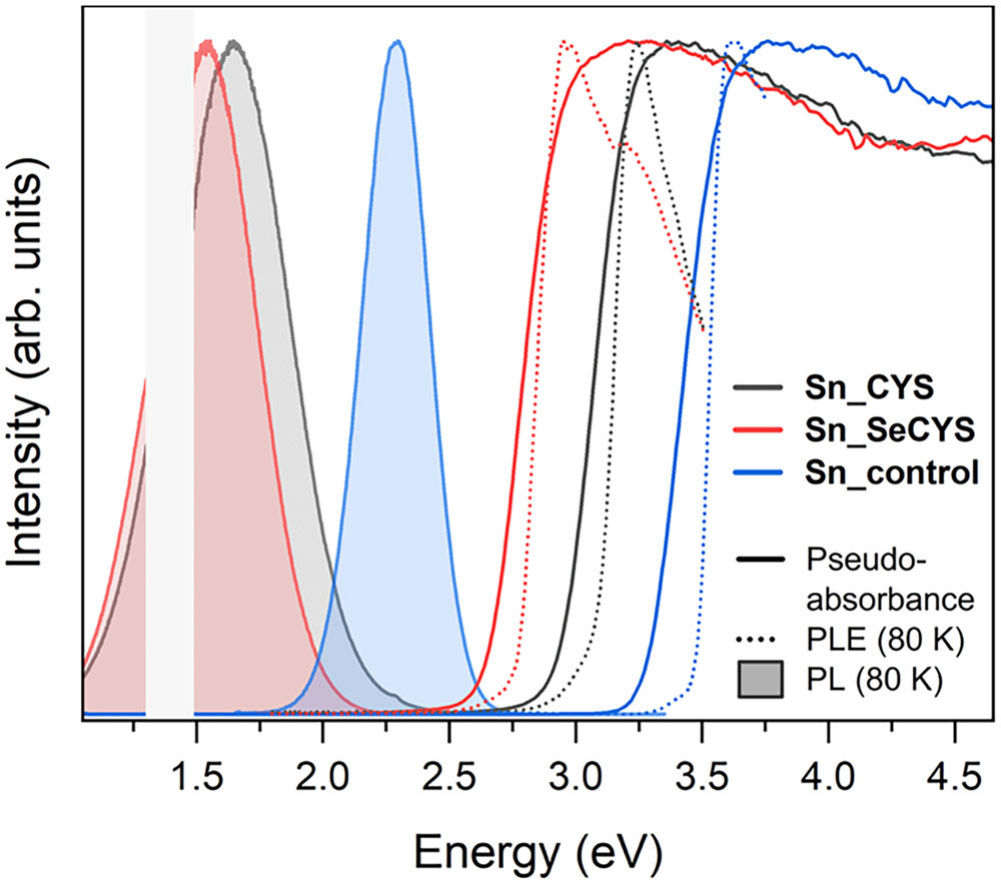
Diffuse reflectance pseudo‐absorbance (room temperature, solid lines), photoluminescence (PL) spectra (80 K, filled lines), and photoluminescence excitation (PLE) spectra (80 K, dotted lines) of powders of **Sn_CYS** (grey), **Sn_SeCYS** (red), and **Sn_control** (blue). The signals measured by visible and infrared detectors were normalized arbitrarily such that the PL signal appears continuous. The shaded gray rectangle indicates the energy window outside the detectors, where the measured intensity is not reliable. For PL spectra, **Sn_RCh** were excited at 377 nm, and **Sn_control** was excited at 340 nm. For PLE spectra, the emission was monitored at 730 nm, 775 nm, and 540 nm for **Sn_CYS**, **Sn_SeCYS**, and **Sn_control**, respectively.

Diffuse reflectance spectra of **Sn_CYS** and **Sn_SeCYS** powders show absorption onsets of 2.94 eV and 2.65 eV, respectively (Figure 4). The diffuse reflectance spectrum of **Sn_control** powder showed a higher absorption onset of 3.28 eV, comparable to those of other 2D tin‐chloride perovskites (ca. 3.2–3.5 eV).^[^ ^42^, ^55^, ^56^ ^]^ The lower‐energy absorption onsets for **Sn_RCh**, compared to those of tin‐chloride perovskites with roughly comparable octahedral distortion (Table S6), as well as the shift of the absorption onset energy based on the chalcogen identity, indicated that this lowest‐energy optical transition involved electronic states from the intergrowth layer. This conclusion agrees with the electronic structure calculations, which showed that both band edges in **Sn_RCh** are mostly derived from the intergrowth layer. Although we expect the calculated bandgaps to be underestimated at the DFT‐PBE level of theory, the experimental difference in absorption onset between **Sn_CYS** and **Sn_SeCYS** (0.29 eV) agrees well with our DFT predictions of the Z‐point direct bandgap difference (0.26 eV), corroborating the inclusion of chalcogen states at the VBT.

Steady‐state photoluminescence (PL) measurements of **Sn_RCh** powders showed a broad emission at 80 K centered at 1.6 eV (**Sn_CYS**) and 1.5 eV (**Sn_SeCYS**). Due to the non‐overlapping ranges of the visible‐ and infrared‐range detectors, the PL spectra of **Sn_RCh** are constructed from two separate measurements and normalized to form a continuous spectrum (Figure 4). The 80‐K PL spectrum of **Sn_control**, excited at 340 nm, shows a broad peak centered at 2.3 eV. Powders of **Sn_CYS** and **Sn_SeCYS** show similar Stokes shifts as that of **Sn_control** (ca. 1.3 eV for **Sn_control**, 1.4–1.6 eV for **Sn_RCh**), and similar emission width (full width at half maximum of ca. 0.3 eV for **Sn_control**; ca. 0.5 eV for **Sn_RCh**). This PL quenches rapidly upon warming (Figure S17), like the PL of **Pb_CYS** centered at 1.8 eV at 80 K (Figure S18).^[^ ^29^ ^]^ Layered tin‐chloride perovskites have been reported to show broad emission centered between 2.1–2.4 eV with a Stokes shift greater than 1 eV at low temperatures (e.g., 12 K).^[^ ^42^, ^56^, ^57^ ^]^

The photoluminescence excitation (PLE) spectra of the broad emission in **Sn_RCh** powders at 80 K (Figure 4) showed similar onsets as those of their room‐temperature diffuse reflectance spectra, suggesting that the emission arises from bandgap excitation, and not from sub‐gap defect states. This conclusion is corroborated by power‐dependent PL measurements of a single crystal of **Sn_CYS** at 80 K (Figure S19). A fit to a power law, *I* ∼ *P^k^* where *P* is the excitation power and *I* is the integrated intensity of the broad emission (from 554–905 nm) gave *k* = 0.98, consistent with PL from exciton recombination (after taking the PL lifetime into account, see the Supporting Information). In contrast, emission from defects shows a sublinear dependence on excitation power as defects are saturated at high excitation power densities.^[^ ^58^ ^]^

The similarity of the spectral shapes (Figure S20) and of the PL decay times (Table S12) of **Sn_RCh** obtained from collections of as‐synthesized crystals and from their pulverized powders also indicates that the PL originates largely from the bulk structure instead of surface sites.^[^ ^59^, ^60^ ^]^ The PL decays of **Sn_RCh** crystals at 77 K with 372 nm excitation can be modeled by a single‐exponential fit, with time constants of ca. 4.4 µs and ca. 3.7 µs for **Sn_CYS** and **Sn_SeCYS**, respectively (Figure S21). These PL lifetimes are slightly longer than the PL lifetime of crystals of **Sn_control** at 77 K with 331 nm excitation, with a time constant of ca. 1.9 µs. The lifetimes of the broad emission of **Sn_RCh** crystals also did not show a significant dependence on the emission wavelength (Table S12), as previously seen in white‐light‐emitting 2D lead‐bromide perovskites.^[^ ^61^, ^62^ ^]^

Overall, the broad emission in **Sn_RCh** appears to be excitonic, arising from the bulk material, and shows features reminiscent of the white‐light‐emitting 2D lead‐chloride and lead‐bromide perovskites, where the PL is attributed primarily to radiative decay of self‐trapped excitons.^[^ ^60^, ^63^, ^64^ ^]^ However, our experimental absorption onsets and calculated band structures point to the lowest‐energy optical absorption in **Sn_RCh** occurring mostly within the intergrowth layer. Thus, we tentatively propose that a similar self‐trapping also occurs in the tin‐halide‐chalcogenide intergrowth layers in **Sn_RCh**. Indeed, the intergrowth layer can be structurally derived from the PbCl_2_ structure, where self‐trapping has been extensively studied, albeit with much higher excitation energies (>4.5 eV).^[^ ^65^, ^66^ ^]^ The localization of the self‐trapped exciton in the intergrowth layer may cause the longer PL lifetimes seen in **Sn_RCh** compared with that of **Sn_control**.

### Photoluminescence Anisotropy in Sn_CYS

**Figure 5:**
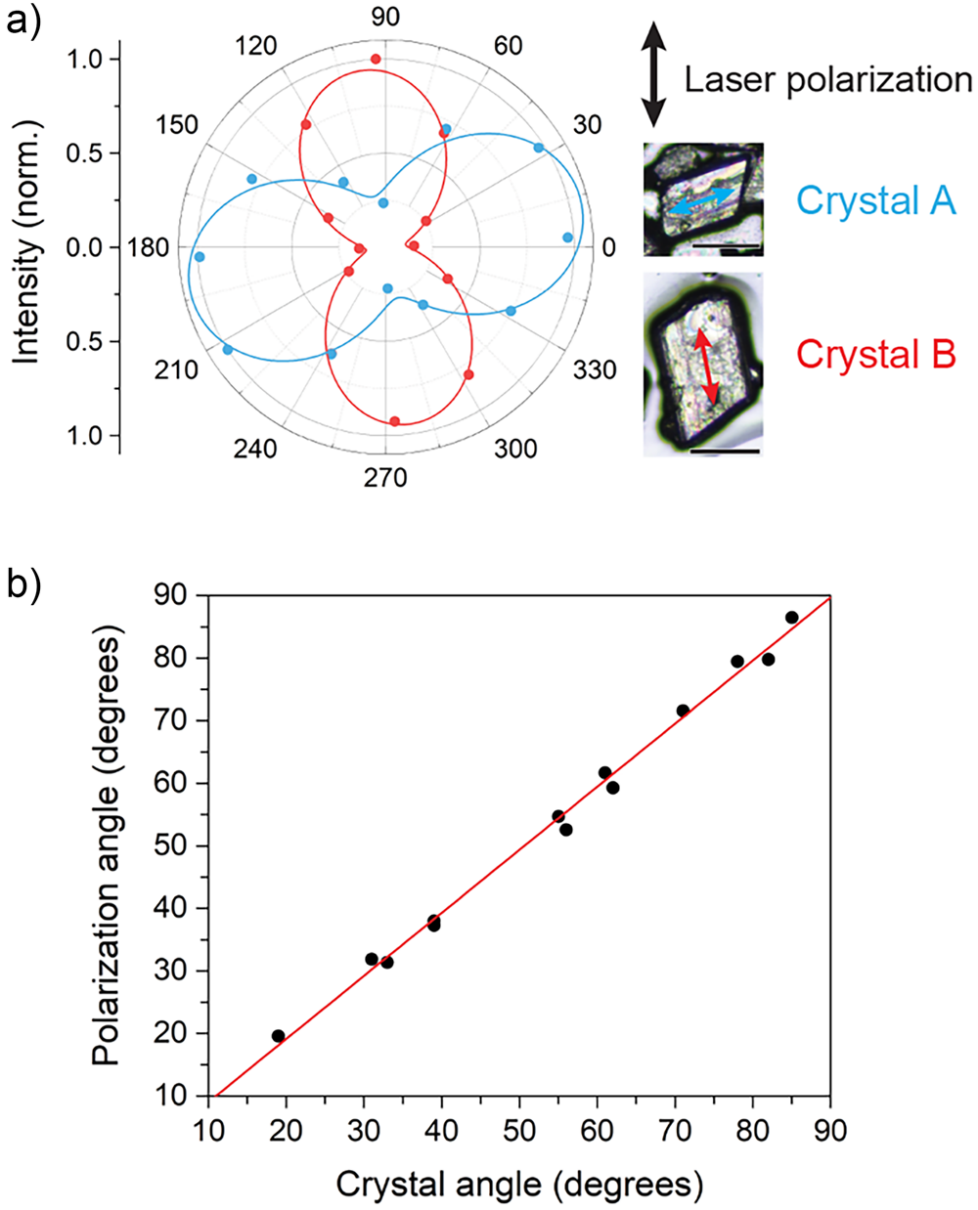
a) Polar plots of PL intensity as a function of the emission polarization angle for single crystals of **Sn_CYS** at 80 K, with 377 nm vertically polarized excitation. Arrows indicate the direction of the crystallographic *a*‐axis in the *P* $\bar{1}$ structures. Scale bars represent 100 µm. b) Linear relationship between the crystal angle, defined as the acute angle of the *a*‐axis with respect to horizontal, and the angle of the major axis of the emission polarization.

The anisotropic structures of **Sn_RCh** motivated us to look for evidence of optical anisotropy. We first examined the in‐plane optical emission anisotropy of **Sn_CYS**. The PL from **Sn_CYS** single crystals was measured at 80 K using linearly polarized 377 nm laser excitation incident perpendicular to the intralayer planes (Figure S1, S22). The direction of the laser polarization was consistent throughout all measurements (Figure 5a, S22). To assess the angle of the linearly polarized PL, we inserted a rotatable linear polarizer in the emission path and measured the total emission intensity as a function of the polarizer angle. The emission was strongly linearly polarized and oriented along the long axis of the blade‐shaped crystals, independent of how the crystal was rotated with respect to the laser polarization (Figure 5a). This long crystal axis, corresponding to the crystallographic *a*‐axis in the *P* $\bar{1}$ SC‐XRD structure, lies perpendicular to the dimer chains in the intergrowth layer (Figure 2, S1).

To rigorously quantify the degree of polarization, we simultaneously measured the vertically polarized (*I*_V_) and horizontally polarized (*I*_H_) components of the PL through a Wollaston prism, and integrated the PL intensities over a spectral range of 554–905 nm. The optical system was calibrated to account for any inherent polarization bias throughout the PL collection optical components. We measured *I*_V_/*I*_H_ for 13 individual crystals, with various *a*‐axis orientations with respect to laser polarization direction, where the crystal *ab* planes were parallel to the substrate and perpendicular to the laser propagation direction (Figure S22; Supporting Discussion 5.2). We define the crystal angle as the acute angle of the *a*‐axis with respect to the horizontal. Fitting the ratio of *I*_V_/*I*_H_ as a function of the crystal angle allowed us to calculate the in‐plane PL anisotropy ratio, AR *= I*_major_/*I*_minor_, defined as the ratio of the intensities of the polarized emission along the major and minor polarization axes (i.e., the maximum possible value of *I*_V_/*I*_H_). We obtain AR = 9.5(3), consistent with the maximum value of *I*_V_/*I*_H_ = 9.2 for a crystal oriented at an angle close to 90°. Our fit model also yielded the angle with respect to the horizontal of the major emission axis for each crystal (the “polarization angle”). The direction of the PL linear polarization correlated well with the crystal longitudinal direction, with a slope of 1.01(2) and an intercept of –1(1)° for a plot of polarization angle vs. crystal angle (Figure 5b), indicating that the major emission axis was consistently aligned with the crystallographic *a*‐axis.

We compared the optical anisotropy of **Sn_CYS** with that of other 2D halide perovskites (Figure S24). Crystals of **Sn_CYS** demonstrated significantly larger in‐plane anisotropy than crystals of (histammonium)PbBr_4_ (maximum AR *=* 1.7 at 80 K) or (phenethylammonium)_2_SnI_4_ (maximum AR = 1.2 at 80 K).^[^ ^59^, ^67^ ^]^ To our knowledge, the maximum in‐plane anisotropy ratio we measured for **Sn_CYS** rivals the highest PL anisotropy ratios previously reported for halide perovskites and low‐dimensional lead‐halide materials^[^ ^68^, ^69^, ^70^, ^71^, ^72^, ^73^, ^74^, ^75^, ^76^ ^]^ (AR up to 11.4 for 1D C_4_N_2_H_14_PbBr_4_ at room temperature),^[^ ^73^ ^]^ as well as those of individual perovskite nanomaterials with 1D morphologies^[^ ^77^, ^78^, ^79^ ^]^ (AR up to 5.9 for a (CH_3_NH_3_)PbI_3_ nanowire at 152 K),^[^ ^77^ ^]^ between 77 and 300 K. For the related perovskite heterostructure (PbBr_2_)_2_(AMTP)_2_PbBr_4_ (AMTP = 4‐ammoniomethyl‐tetrahydropyran),^[^ ^29^ ^]^ an in‐plane absorption anisotropy ratio of 1.6 has been reported for photocurrent with linearly polarized excitation, attributed to unequal absorption coefficients along the two in‐plane axes due to the asymmetry of the intergrowth layer.^[^ ^80^ ^]^ Whereas the maximum photocurrent for (PbBr_2_)_2_(AMTP)_2_PbBr_4_ was obtained with excitation polarized along the *c*‐axis, parallel to the dimer chains in the intergrowth layer, the emission in **Sn_CYS** is polarized along the *a*‐axis, perpendicular to these dimer chains. Notably, the calculated lowest‐energy optical transition in (PbBr_2_)_2_(AMTP)_2_PbBr_4_ occurs solely within the perovskite layer,^[^ ^29^ ^]^ whereas in **Sn_CYS**, we anticipate that this transition has contributions from both layers, allowing the structural anisotropy of each layer to contribute to the optical anisotropy of the “twisted” heterostructure. We propose that the extremely elongated Sn–Cl bond (ca. 3.8 Å) along the *b*‐axis of the perovskite layer and the shorter Sn–(*μ*‐Cl)–Sn bonds in the intergrowth layer, which lie primarily along the *a*‐axis direction, result in a quasi‐1D structure with polarized emission preferentially oriented along the *a*‐axis direction. The anisotropy of the crystal structure could also affect excited‐state distortions that occur with exciton self‐trapping. Thus, anisotropic optical absorption and the coupling of anisotropic distortions to the self‐trapped exciton may both contribute to the observed emission anisotropy.^[^ ^81^ ^]^

To confirm that optical anisotropy in **Sn_CYS** arises from intrinsic structural anisotropy, rather than solely from excited‐state distortions, we also measured the in‐plane birefringence of crystals of **Sn_CYS**. We measured the integrated intensities of the vertically polarized (*R*_V_) and horizontally polarized (*R*_H_) components of the reflectance from **Sn_CYS** crystals, using a sub‐bandgap 730 nm vertically polarized laser source. Reflectance from the substrate alone preserved the vertical polarization state, with *R*_V_/*R*_H_ = 101. By contrast, reflectance from the crystals showed a modulation of the polarization, with *R*_V_/*R*_H_ reaching a minimum of 0.4 (Figure S25), indicating distinct optical axes within the intralayer planes. The birefringence is also corroborated by the appearance of interference colors under cross‐polarized illumination (Figure S26). This in‐plane birefringence further supports the in‐plane optical anisotropy resulting from the anisotropic crystal structure.

### Alloying the Metal Sites

**Figure 6:**
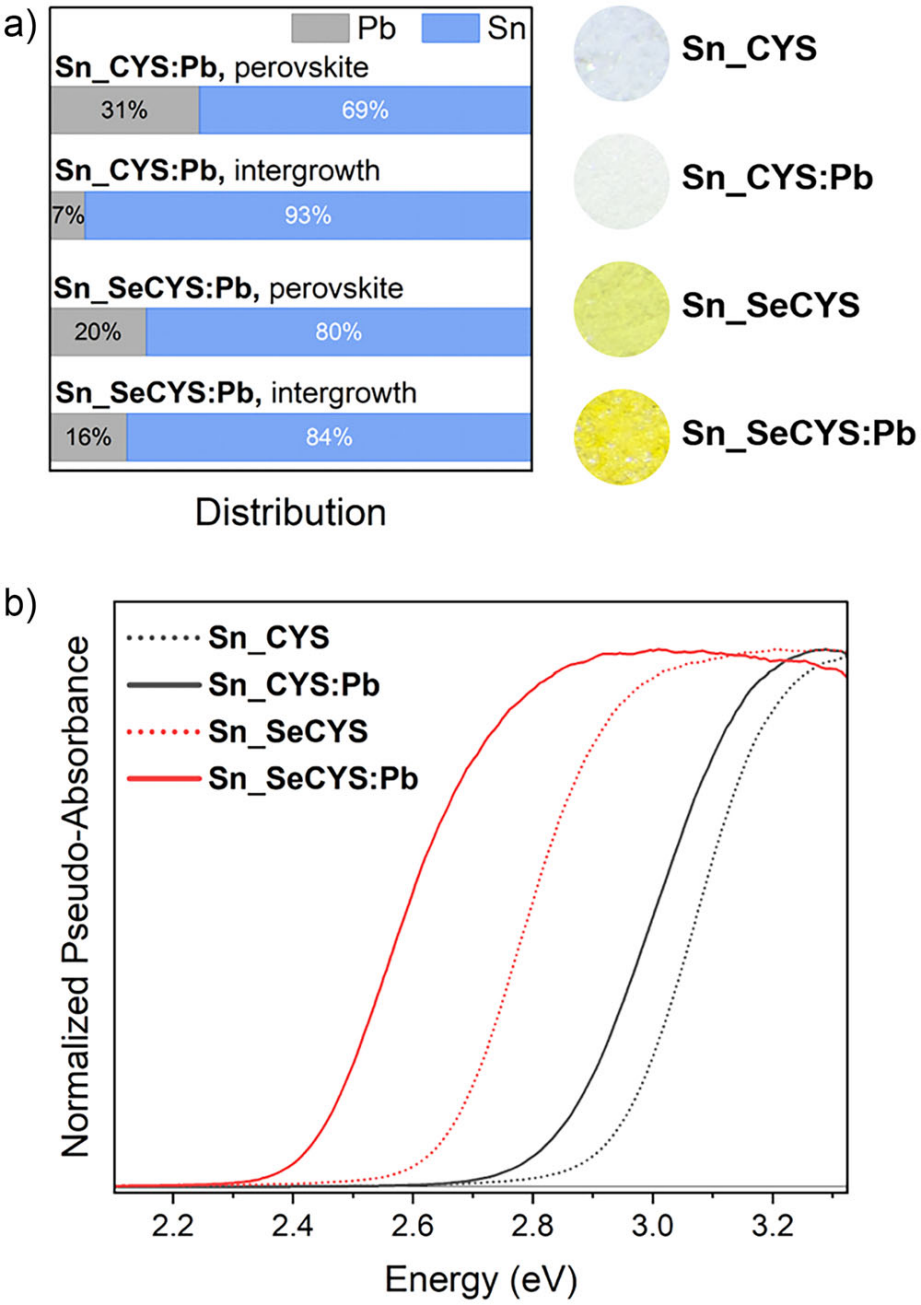
a) Distribution of Pb and Sn occupancies in the perovskite and intergrowth layers of **Sn_RCh:Pb** alloys. Photographs of **Sn_RCh** and **Sn_RCh:Pb** powders show color changes upon incorporation of Pb. b) Diffuse reflectance spectra of powders of **Sn_RCh:Pb** alloys compared with powders of **Sn_RCh**.

The band‐edge orbital compositions in the **Sn_RCh** heterostructures allow the absorption onsets to be further tuned through alloying. We prepared single crystals of the alloyed heterostructures (Sn_1.85_Pb_0.15_Cl_2_)(CYS)_2_Sn_0.69_Pb_0.31_Cl_4_ (**Sn_CYS**:**Pb**) and (Sn_1.68_Pb_0.32_Cl_2_)(SeCYS)_2_Sn_0.80_Pb_0.20_Cl_4_ (**Sn_SeCYS**:**Pb**), which are isostructural to **Sn_RCh**. Relative occupancies of the metal sites of **Sn_RCh:Pb** were estimated from occupancy refinements of SC‐XRD solutions in the *C*2/*c* space group at 100 K. The distribution of Pb and Sn across the perovskite and intergrowth metal sites differed for **Sn_CYS:Pb** and **Sn_SeCYS:Pb** (Figure 6a). For **Sn_SeCYS**:**Pb**, the metal sites in the perovskite and intergrowth layers had occupancies of 20% Pb and 16% Pb, respectively, with 17% Pb in total. For **Sn_CYS**:**Pb**, the total amount of Pb was comparable (15%), but the metal‐site occupancy in the perovskite layer was 31% Pb whereas the metal‐site occupancy in the intergrowth layer was only 7.3% Pb. We attribute these differences in metal‐site occupancies to the larger lattice parameters for **Sn_SeCYS** compared to those for **Sn_CYS**, where the former allows for more substitution of Sn^2+^ with the larger Pb^2+^ cation in the areally compressed intergrowth layer.^[^ ^82^ ^]^ Relative occupancies obtained from SC‐XRD solutions of multiple crystals showed good consistency in the occupancy trends for both **Sn_CYS**:**Pb** (22%–31% Pb in the perovskite layer; 7%–8% Pb in the intergrowth layer over 3 crystals) and **Sn_SeCYS:Pb** (20%–25% Pb in the perovskite layer; 15%–16% Pb in the intergrowth layer over 2 crystals).

Although **Pb_CYS** has a slightly higher‐energy absorption onset than **Sn_CYS**, alloying small amounts of Pb into **Sn_RCh** caused the absorption onset to shift to even lower energies (Figure 6b). A similar redshift upon alloying has been observed when small fractions of Pb are introduced into Sn halide perovskites.^[^ ^32^, ^83^, ^84^, ^85^, ^86^ ^]^ This redshift is due to the energy mismatch of Pb and Sn frontier orbitals: lower‐energy Pb 6p states lower the CBB, whereas the VBT remains dominated by higher‐energy Sn 5s states.^[^ ^32^ ^]^ The absorption onset of **Sn_CYS:Pb** is smaller than the absorption onset of **Sn_CYS** by ca. 0.1 eV. This redshift in the alloy may be attributed to contributions from lower‐energy Pb 6p states to the CBB (which in **Sn_CYS** is dominated by Sn 5p states from the intergrowth layer) whereas the VBT remains comprising mostly Sn states from the intergrowth layer. For **Sn_SeCYS**, Pb alloying yields a larger decrease in absorption onset of ca. 0.2 eV (Figure 6b) due to the larger fraction of Pb in the intergrowth metal site, which is the dominant contributor to the CBB. These trends in absorption onset as a function of the composition of each layer are thus wholly consistent with the calculated band structures. Here, the interfacial lattice‐matching constraints and the consequent distinct Pb‐Sn alloying ratios in each layer offer an additional level of tunability in the band‐edge compositions.

## Conclusion

We present the new single‐crystal heterostructures (Sn_2_Cl_2_)(*RCh*)_2_SnCl_4_ (*RCh* = CYS, SeCYS) with interleaving perovskite and non‐perovskite intergrowth layers. Unlike in manually stacked monolayers, these single‐crystalline solids afford structural resolution of the repeating interfaces. Single‐crystal X‐ray diffraction and pair distribution function analysis revealed large octahedral distortions in the perovskite layer due to stereochemical expression of the Sn 5s^2^ lone pair. Notably, this distortion enables a twist between the layers compared with the structure of the (Pb_2_Cl_2_)(CYS)_2_PbCl_4_ analog. We find large, anisotropic lattice strain in both layers of (Sn_2_Cl_2_)(*RCh*)_2_SnCl_4_, compared to slices of the parent (CH_3_NH_3_)SnCl_3_ and SnCl_2_ structures, due to interfacial lattice‐matching requirements.

The distortions imposed by the lone‐pair expression and lattice matching of the perovskite and intergrowth layers impact the electronic structure, and thus optical properties, of (Sn_2_Cl_2_)(*RCh*)_2_SnCl_4_. The photoluminescence resembles that of 2D Sn and Pb perovskites, with signatures of exciton self‐trapping. However, the mixed perovskite and intergrowth states at the valence band edge in (Sn_2_Cl_2_)(CYS)_2_SnCl_4_ predicted by DFT may induce a different localization of the self‐trapped exciton compared to the trapping sites in typical perovskites, with the possibility of an interlayer exciton, motivating further studies. The structural anisotropy imparted by the twisted structure of (Sn_2_Cl_2_)(CYS)_2_SnCl_4_ also gives rise to in‐plane optical anisotropy. Single crystals of (Sn_2_Cl_2_)(CYS)_2_SnCl_4_ achieve linearly polarized luminescence within the intralayer planes, aligned with the crystallographic *a*‐axis, with a linear anisotropy ratio of up to 9.5.

Overall, we demonstrate that local distortions, and the resulting interfacial strain between two different lattices forced to share a unit cell, can induce a twist between the layers of a self‐assembled heterostructure, potentially setting the stage for deliberate manipulation of twist angle and interfacial strain in bulk materials.

## Supporting Information

Experimental and computational details, crystallographic data, and additional characterization.

The authors have cited additional references within the Supporting Information.^[^ ^87^, ^88^, ^89^, ^90^, ^91^, ^92^, ^93^, ^94^, ^95^, ^96^, ^97^, ^98^, ^99^, ^100^, ^101^, ^102^, ^103^, ^104^, ^105^, ^106^, ^107^, ^108^, ^109^, ^110^, ^111^, ^112^, ^113^, ^114^, ^115^, ^116^, ^117^, ^118^ ^]^

The structural models used for DFT calculations and the model of **Sn_CYS** from PDF analysis are provided as additional CIFs in the Supporting Information.

## Conflict of Interests

The authors declare no conflict of interest.

## Supporting information



Supporting Information

Supporting Information

## Data Availability

The data that support the findings of this study are openly available in the Stanford Digital Repository at https://doi.org/10.25740/vs141dv6379, reference number [120].
