# Supplementary material for: Twisted Tin‐Chloride Perovskite Single‐Crystal Heterostructures
Source: Angew Chem Int Ed Engl. 2025 Dec 19;65(5):e20140. doi: 10.1002/anie.202520140 (PMC12851008; doi:10.1002/anie.202520140)
Supplement: Supplementary file 1 — Supporting Information [file ANIE-65-e20140-s002.pdf]

Supporting information for:

Twisted Tin-Chloride Perovskite Single-Crystal Heterostructures

Jamie L. Cleron,[a] Chih-Yi Chen,[b] Feng Pan,[b] Santanu Saha,[c,d] Frederick P. Marlton,[a,e] Robert M. Stolz,[a] Jiayi Li,[a] Jennifer A. Dionne,[b] Fang Liu,[a] Marina R. Filip,[*,c] Hemamala I. Karunadasa[*,a,f]

[a]*Department of Chemistry, Stanford University, Stanford, California 94305, United States*

[b]*Department of Materials Science and Engineering, Stanford University, Stanford, California 94305, United States*

[c]*Department of Physics, University of Oxford, Clarendon Laboratory, Parks Road, Oxford OX1 3PU, United Kingdom*

[d]*Institut de Recherche sur les Ceramiques (IRCER), UMR CNRS 7315-Université de Limoges, 12 Rue Atlantis, Limoges 87068, France*

[e]*School of Mathematical and Physical Sciences, Faculty of Science, University of Technology Sydney, Sydney, NSW 2007, Australia*

[f]*Stanford Institute for Materials and Energy Sciences (SIMES), SLAC National Accelerator Laboratory, Menlo Park, California 94025, United States*

*hemamala@stanford.edu, marina.filip@physics.ox.ac.uk

Table of Contents

1. Materials

1.1 Synthesis of $(Sn_2Cl_2)(CYS)_2SnCl_4$ (**Sn_CYS**) S3

1.2 Synthesis of $(Sn_2Cl_2)(SeCYS)_2SnCl_4$ (**Sn_SeCYS**) S3

1.3 Synthesis of $(Sn_{1.85}Pb_{0.15}Cl_2)(CYS)_2Sn_{0.69}Pb_{0.31}Cl_4$ (**Sn_CYS:Pb**) S4

1.4 Synthesis of $(Sn_{1.68}Pb_{0.32}Cl_2)(SeCYS)_2Sn_{0.80}Pb_{0.20}Cl_4$ (**Sn_SeCYS:Pb**) S4

1.5 Synthesis of $(T4YMA)_2SnCl_4$ (**Sn_control**) S4

1.6 Synthesis of $(Pb_2Cl_2)(CYS)_2PbCl_4$ (**Pb_CYS**) S4

1.7 Synthesis of $(BZA)_2SnCl_4$ S5

1.8 Synthesis of $(HIS)PbBr_4$ S5

1.9 Synthesis of $(PEA)_2SnI_4$ S5

1.10 Thin-film preparation S6

2. Methods

2.1 Single-crystal X-ray diffraction (SC-XRD) S6

2.2 Powder X-ray diffraction (PXRD) S7

2.3 X-ray pair distribution function (PDF) analysis S7

2.4 Diffuse reflectance spectroscopy S8

2.5 Thin-film absorption S8

2.6 Photoluminescence spectroscopy S8

2.7 Time-resolved photoluminescence S9

2.8 Linearly-polarized photoluminescence spectroscopy S9

2.9 Power-dependent photoluminescence spectroscopy S10

2.10 Polarized microscopy S10

2.11 Raman spectroscopy S10

2.12 Stability tests S10

2.13 Strain calculations S11

2.14 Computational methodology S11

3. Tables S1–S12 S12

4. Figures S1–S35 S21

5. Supplementary Discussion

5.1 PL intensity dependence on excitation power density S45

5.2 Derivation of polarization angle for linearly-polarized PL S46

6. References S48

1. Materials

All reagents were purchased from commercial vendors and used as received. Solvents were of reagent grade or higher purity. Solutions of concentrated aqueous NaCl were prepared by combining 5.26 g of NaCl and 15 mL of deionized water (ca. 5.3 M). Abbreviations used: CYS = cysteamine, SeCYS·HCl = selenocysteamine hydrochloride, T4YMA = thian-4-ylmethanammonium, BZA = benzylammonium, HIS = histammonium, PEA = phenethylammonium, DMF = N,N-dimethylformamide, PMMA = poly(methyl methacrylate) (average Mw ≈ 120,000 by GPC). Samples were stored under inert atmosphere. C, H, N elemental analysis was performed under air-free conditions by Midwest Microlab (Indianapolis, IN). All C, H, N analyses are reported as the average of duplicate measurements.

1.1 Synthesis of $(Sn_2Cl_2)(CYS)_2SnCl_4$ (Sn_CYS)

In a N_2 atmosphere, solid $SnCl_2$ (150 mg, 0.791 mmol) was added to 0.80 mL of aqueous NaCl in a 4 mL scintillation vial. The solution was heated at 95 ºC until the solids fully dissolved. Then CYS (42 mg, 0.54 mmol) was dissolved in 0.65 mL of H_2O in a 4 mL scintillation vial and briefly heated at 95 ºC. The CYS solution was added to the $SnCl_2$ solution, producing a colorless powder. The suspension was kept at 95 ºC for 10–15 minutes followed by hot filtration, producing a clear, colorless solution. The hot plate was turned off and the vial was cooled to room temperature overnight to afford colorless, blade-like crystals of **Sn_CYS**. In ambient conditions, the product was isolated by filtration and dried under reduced pressure. A typical yield was 65 mg of **Sn_CYS** (34% yield with respect to Sn). Elemental analysis. Calculated: C 6.64%, H 1.95%, N 3.87%. Found: C 6.64%, H 1.95%, N 3.83%. Single crystals used for optical measurements were isolated in Paratone-N® oil after filtration, then rinsed with toluene to remove the residual oil.

Crystals used to obtain SC-XRD crystal structures were formed by removing the vial from the hot plate, allowing it to cool naturally to room temperature, and leaving the vial at room temperature overnight.

1.2 Synthesis of $(Sn_2Cl_2)(SeCYS)_2SnCl_4$ (Sn_SeCYS)

The SeCYS·HCl ligand was synthesized based on a reported method.[1,2]

In a N_2 atmosphere, solid $SnCl_2$ (150 mg, 0.791 mmol) was added to 2.4 mL of aqueous NaCl in a 4 mL scintillation vial. The solution was heated at 95 ºC until the solids fully dissolved. Then SeCYS·HCl (42 mg, 0.26 mmol) was dissolved in 0.1 mL of H_2O in a 4 mL scintillation vial at room temperature. To the SeCYS·HCl solution, 0.174 mL of 1.5 M aqueous NaOH was added. To the $SnCl_2$ solution, 0.1 mL of hypophosphorous acid (50% w/v aqueous solution) was added. The SeCYS solution was quickly added to the $SnCl_2$ solution, forming a yellow solution that turned colorless over several minutes at 95 ºC. The vial was sealed in the N_2 atmosphere, then quickly transferred to a hot plate in ambient atmosphere at 95 ºC. Cooling from 95 ºC to 25–35 ºC at a rate of 3 ºC/h yielded yellow, blade-like crystals of **Sn_SeCYS**. The product was isolated by filtration and dried under reduced pressure. A typical yield was 70 mg of **Sn_SeCYS** (65% yield with respect to SeCYS). Elemental analysis. Calculated: C 5.88%, H 1.73%, N 3.43%. Found: C 5.84%, H 1.70%, N 3.42%.

Small amounts of NaCl may be present in samples of **Sn_RCh** used for bulk measurements from the concentrated aqueous NaCl solvent used in the synthesis. The amount of residual NaCl can be reduced to <1 weight percent (based on Rietveld refinement of synchrotron PXRD data) by rinsing with dilute aqueous

NaCl during filtration (Figure S32). Yields were calculated based on rinsed samples. Diffuse reflectance and photoluminescence spectra of rinsed and unrinsed samples showed negligible differences.

1.3 Synthesis of ($Sn_{1.85}Pb_{0.15}Cl_2$)(CYS)$_2$$Sn_{0.69}Pb_{0.31}Cl_4$ (Sn_CYS:Pb)

In a N_2 atmosphere, solid $SnCl_2$ (135 mg, 0.712 mmol) and $PbCl_2$ (22 mg, 0.079 mmol) were added to 0.80 mL of aqueous NaCl in a 4 mL scintillation vial. The solution was heated at 95 ºC until the solids fully dissolved. Then CYS (42 mg, 0.54 mmol) was dissolved in 0.65 mL of H_2O in a 4 mL scintillation vial and briefly heated at 95 ºC. The CYS solution was added to the $SnCl_2$/$PbCl_2$ solution, producing a pale yellow powder. The suspension was kept at 95 ºC for 10–15 minutes followed by hot filtration, producing a clear, pale-yellow solution. After cooling by turning off the hot plate and leaving the vial at room temperature overnight, pale yellow blade-like crystals of **Sn_CYS:Pb** were formed along with a small amount of tree-shaped pale-yellow polycrystalline material. In ambient conditions, the products were then isolated by filtration, and crystals of **Sn_CYS:Pb** were isolated from the side phase. The phase purity of isolated crystals was confirmed by PXRD.

1.4 Synthesis of ($Sn_{1.68}Pb_{0.32}Cl_2$)(SeCYS)$_2$$Sn_{0.80}Pb_{0.20}Cl_4$ (Sn_SeCYS:Pb)

In a N_2 atmosphere, solid $SnCl_2$ (43 mg, 0.23 mmol) and $PbCl_2$ (7.0 mg, 0.025 mmol) were added to 1.5 mL of aqueous NaCl in a 4 mL scintillation vial. The solution was heated at 95 ºC until the solids fully dissolved. Then SeCYS·HCl (13 mg, 0.081 mmol) was dissolved in 0.5 mL of H_2O in a 4 mL scintillation vial at room temperature. The SeCYS solution was added to the $SnCl_2$/$PbCl_2$ solution. The vial was sealed in the N_2 atmosphere, then quickly transferred to a hot plate in ambient atmosphere at 95 ºC. Cooling from 95 ºC to 25–35 ºC at a rate of 2 ºC/h yielded yellow-orange blade-like crystals of **Sn_SeCYS:Pb**, along with a pale yellow needle-shaped side phase. The products were isolated by filtration. The products were then dispersed in Paratone-N® oil, and crystals of **Sn_SeCYS:Pb** were isolated from the side phase and rinsed with hexanes to remove residual oil. The phase purity of isolated crystals was confirmed by PXRD.

1.5 Synthesis of (T4YMA)$_2$$SnCl_4$ (Sn_control)

In a N_2 atmosphere, solid $SnCl_2$ (67 mg, 0.35 mmol) and thian-4-ylmethanamine hydrochloride (120 mg, 0.716 mmol) were added to 1 mL of 6 M HCl in a 4 mL scintillation vial. The solution was heated at 95 ºC until the solids fully dissolved. Then 7 drops of hypophosphorous acid (ca. 120 μL; 50% w/v aqueous solution) were added using a glass pipette. The vial was sealed in the N_2 atmosphere, then transferred to a hot plate in ambient atmosphere at 95 ºC. Cooling from 95 ºC to 25–35 ºC at a rate of 3 ºC/h yielded colorless crystals of (T4YMA)$_2$$SnCl_4$. The product was then isolated by filtration and dried under reduced pressure. A typical yield was 80 mg of **Sn_control** (43% yield with respect to Sn). Elemental analysis. Calculated: C 27.45%, H 5.38%, N 5.34%. Found: C 27.56%, H 5.36%, N 5.27%.

Crystals of (T4YMA)$_2$$SnCl_4$ used for SC-XRD measurements were synthesized using a scaled-down version of the above procedure, in which all reagent quantities are scaled by a factor of 0.15.

1.6 Synthesis of (Pb_2Cl_2)(CYS)$_2$$PbCl_4$ (Pb_CYS)

The reported synthesis for **Pb_CYS** was modified to obtain improved crystals suitable for SC-XRD.[3] Solid lead (II) acetate trihydrate (60 mg, 0.16 mmol) and 2-aminoethanethiol hydrochloride (18 mg, 0.16 mmol) were added to 2 mL of 6 M aqueous LiCl in a 4 mL scintillation vial. To this vial, 1 drop of acetic acid (ca. 5–10 μL) was added. The vial was heated at 115 ºC while stirring until the solids fully dissolved. The hot

plate was turned off and covered with aluminum foil, and the vial was cooled to room temperature overnight to afford colorless, plate-like crystals of **Pb_CYS**.

We obtained a new crystal structure for **Pb_CYS** in the space group *Pnma*. Compared with the reported structure in *Ima*2, this structure is halved along the interlayer axis and all atomic positions are fully ordered, consistent with the "ordered model" previously used for electronic structure calculations.[3]

Phase-pure **Pb_CYS** used for PXRD measurements (Figure S2), was synthesized using a scaled-down version of the above procedure, where the quantities of lead (II) acetate trihydrate, 2-aminoethanethiol hydrochloride, and 6 M aqueous LiCl were scaled by a factor of 0.5. Powders of **Pb_CYS** used for optical measurements (Figure S18) were synthesized according to the previously reported method.[3]

1.7 Synthesis of $(BZA)_2SnCl_4$

The chloride salt of BZA (BZA·Cl) was prepared by adding 2 mL of benzylamine to 5 mL of ethanol. The solution was cooled in an ice bath, and 1.52 mL of 12 M HCl was added dropwise while stirring. The solution was transferred to a petri dish and was heated at 80 ºC. The resulting colorless solid was dried under reduced pressure.

The synthesis of $(BZA)_2SnCl_4$ was adapted from a reported method.[4] In a N_2 atmosphere, solid tin (II) acetate (180 mg, 0.760 mmol) and BZA·Cl (218 mg, 1.52 mmol) were added to 3 mL of 6 M HCl in a 4 mL scintillation vial. The solution was heated at 100 ºC until the solids fully dissolved. Then 0.1 mL of hypophosphorous acid (50% w/v aqueous solution) was added. The vial was sealed in the N_2 atmosphere, then quickly transferred to an ice bath in ambient conditions, leading to the formation of flaky colorless crystals. The product was then isolated by filtration and dried under reduced pressure.

1.8 Synthesis of $(HIS)PbBr_4$

Single crystals of $(HIS)PbBr_4$ were synthesized based on a previously reported method.[5] The solution was cooled to room temperature at a rate of 3 ºC/h to obtain crystals of a size suitable for optical measurements. Crystals were isolated in Paratone-N® oil and rinsed with toluene before securing to a Si substrate with a solution of PMMA in toluene. The identity and (100) orientation of the crystals, with the inorganic planes parallel to the substrate, was confirmed by PXRD (Figures S24, S31).

1.9 Synthesis of $(PEA)_2SnI_4$

The iodide salt of PEA (PEA·I) was prepared by adding 6 mL of phenethylamine to 8 mL of ethanol. The solution was cooled in an ice bath, and 6.36 mL of concentrated HI (47%, with 1.5% hypophosphorous acid) was added dropwise while stirring. The solvent was removed under reduced pressure. The colorless solid was then recrystallized from isopropanol.

Crystals of $(PEA)_2SnI_4$ were synthesized based on a modified reported method.[6] In a N_2 atmosphere, solid SnI_2 (37 mg, 0.099 mmol) and PEA·I (50 mg, 0.20 mmol) were added to 0.25 mL of gamma-butyrolactone in a 4 mL scintillation vial. The solution was heated at 70 ºC briefly until the solids dissolved. The solution was filtered, then transferred to a vapor diffusion chamber with 3 mL of dichloromethane as the antisolvent. In ambient conditions, the dark red plates that formed were isolated in Paratone-N® oil and rinsed with toluene under a stream of flowing N_2 before securing to a Si substrate with a solution of PMMA in toluene.

The identity and (001) orientation of the crystals, with the inorganic planes parallel to the substrate, was confirmed by PXRD (Figures S24, S31).

1.10 Thin-Film preparation

Substrate preparation: All thin films were prepared in a N_2-filled glovebox to exclude moisture. Glass microscope slides were cut, scrubbed with a detergent solution, then sequentially sonicated in detergent solution, DI water, acetone, and isopropanol. The films were then dried with compressed air and treated with UV light and ozone for 15 minutes before being transferred into the glovebox for spinning.

Spincoating: For **Sn_RCh**, two different film preparations were used. Thicker, cloudier films (ca. 60–90 nm) can be prepared from 0.07 M precursor solutions of **Sn_RCh** crystals in DMF. The solution was passed through a 0.22 μm PTFE filter, and 70 μL of the filtered solution was deposited on a substrate spinning at 4500 rpm. The films were spun at 4500 rpm for 1 minute, with 70 μL of dichloromethane deposited on the substrate after 45 s. The films were then annealed at 120 ºC for 5 minutes. Clearer films suitable for optical characterization (ca. 20–40 nm) can be prepared from 0.1 M precursor solutions of **Sn_RCh** crystals in DMF, heated at 60 ºC. The substrates were also heated at 60 ºC. The solution was passed through a 0.22 μm PTFE filter, and 70 μL of the filtered solution was deposited on a substrate spinning at 4500 rpm. The films were spun at 4500 rpm for 1 minute, with 70 μL of diethyl ether deposited on the substrate after 45 s. The films were then annealed at 60 ºC for 15 minutes. Films were stored in a N_2-filled glovebox. PXRD patterns of the **Sn_RCh** films agreed with the simulated patterns from the single-crystal X-ray diffraction structures with (001) preferential orientation (in the $P\bar{1}$ space group).

For films of **Sn_control**, crystals of **Sn_control** were dissolved in DMF at a concentration of 0.1 M. The solution was heated at 80 ºC, then passed through a 0.22 μm PTFE filter. The substrates were also heated at 80 ºC. The solution (70 μL) was deposited on a substrate spinning at 4500 rpm. The films were spun at 4500 rpm for 1 minute, with 70 μL of diethyl ether deposited on the substrate after 45 s. The films were then annealed at 80 ºC for 15 minutes. Films were stored in a N_2-filled glovebox.

Film thickness was measured using a Bruker Dektak XT-A stylus profiler.

2. Methods

2.1 Single-crystal X-ray diffraction (SC-XRD)

Crystals were removed from the mother liquor, coated with Paratone-N® oil or Cargille NVH immersion oil, attached to a Kapton® loop, and transferred to a Bruker D8 diffractometer equipped with a Photon II detector. Fragments of large, thin crystals were used to avoid twin boundaries. The crystals did not show significant decay during data collection. The radiation source was Mo Kα radiation ($\lambda = 0.71073$ Å) at nano@stanford for all structures except the 100 K C2/*c* structure of **Sn_CYS**. For this structure, the radiation source was the synchrotron at the Advanced Light Source (ALS), LBNL ($\lambda = 0.72880$ Å) on beamline 12.2.1. Frames were collected using ω and φ scans and the unit-cell parameters were refined against all data. Frames were integrated and corrected using SAINT V8.38A or V8.40B for Lorentz and polarization effects.[7] The absorption corrections were performed using SADABS-2016/2. Space-group assignments were based upon systematic absences, *E* statistics, agreement factors for equivalent reflections, and successful refinement of the structures. The structures were solved using the intrinsic phasing method implemented in SHELXT.[8] The solutions were refined against all data using the SHELXL-2019/3 software

package[9] and OLEX2.[10] Weighted R factors, R_w, and all goodness-of-fit indicators are based on F^2. A small number of reflections with large errors were omitted from the refinements.

Mixed site occupancies for the metal sites in **Sn_CYS:Pb** and **Sn_SeCYS:Pb** were modeled by using the EADP and EXYZ commands in SHELXL and refining the relative occupancies of Pb and Sn through a free variable. To account for twinning in **Sn_CYS:Pb**, an HKLF5 file was generated in OLEX2 to apply the non-merohedral twin law of [–1.001 0 –1.395 0 –1 0 0.001 0 1.001], BASF = 0.17, corresponding to a twofold rotation about the c axis.[11] A rigid-bond restraint was applied between two carbon atoms to stabilize the anisotropic refinement.

In the $P\bar{1}$ SC-XRD solutions of **Sn_CYS** and **Sn_SeCYS**, occupancies of disordered atoms were first permitted to refine freely. Disordered atoms were sorted into majority- and minority-occupancy parts, and the occupancy of each part was fixed at a value similar to the free occupancies that allowed all disordered atoms to reach a stable refinement. Several DELU, EADP, SIMU, and ISOR commands were used to stabilize the anisotropic refinement of disordered atoms and ensure that all disordered atoms could be refined to the same occupancy, consistent with a model of two superimposed lattices.

2.2 Powder X-ray diffraction (PXRD)

PXRD data were collected using a Bruker D8 Advance Diffractometer equipped with a Cu anode ($K\alpha_1$ = 1.54060 Å, $K\alpha_2$ = 1.54443 Å, $K\alpha_2/K\alpha_1$ = 0.5), fixed divergence slits with a nickel filter and a LYNXEYE 1D detector. The instrument was operated in a Bragg-Brentano geometry with a step size of 0.02° (2θ). Crystals were ground into a powder in a mortar and pestle. The powders were dispersed in toluene and transferred to a clean glass slide or silicon sample holder. For determination of crystal orientations (Figures S1, S22), crystals were secured to a silicon substrate with a solution of PMMA in toluene. The measurements were conducted under ambient atmosphere. Pawley refinements (Figure S2) were performed in the GSAS-II software.[12] Simulated powder patterns were calculated directly from the crystallographic information files (CIFs) using the Mercury software.

High-resolution PXRD patterns of **Sn_CYS** and **Sn_SeCYS** were measured at beamline 2-1 at the Stanford Synchrotron Radiation Lightsource at SLAC National Accelerator Laboratory.[13] Powders were packed into a glass capillary with 0.5 mm outer diameter. Measurements were performed at 295 K, using λ = 0.72947 Å synchrotron radiation with sample spinning to ensure appropriate averaging statistics. 2D Debye-Scherrer diffraction rings from powder measurements were collected on a PILATUS 100K small area detector and were integrated using a custom Python script developed at beamline 2-1. The X-ray wavelength and zero error were calibrated using a refinement of LaB_6 standard in the software package TOPAS v7.[14] Rietveld refinements of the integrated 1D diffraction patterns for **Sn_CYS** and **Sn_SeCYS** (Figure S3) were performed in TOPAS v7.

2.3 X-ray pair distribution function analysis

X-ray total scattering measurements were performed at beamline I15-1 of Diamond Light Source, UK (λ = 0.161669 Å, 76.69 keV). The powder sample was loaded into a glass capillary with an outer diameter of 1 mm. Data were collected under ambient conditions and at 100 K for the sample and an empty capillary. Diffraction data were collected using a Perkin Elmer area detector with an exposure time of 600 seconds. 2D diffraction data were corrected for the polarization, detector transmission, and flat field, and badly

performing pixels were masked prior to integration to 1D using DAWN.[15] The PDFs $G(r)$ functions were calculated using PDFgetX3[16] software from the xPDFsuite[17] with a Q_{max} of 20 $Å^{-1}$.

Refinements against the PDF data were carried out using the software package TOPAS v6.[14] A spherical correlation function was used to model the change in B_{iso} with respect to r in the PDF refinements. Refinements were repeated to obtain the global minimum using the continue_after_convergence and val_on_continue functions in TOPAS.

As an initial model, we constructed an ordered model based on the 100 K $P\bar{1}$ SC-XRD solution of **Sn_CYS**, where we fixed the positions of disordered atoms to occupy only the majority-occupation sites and removed the minority-occupation sites. Hydrogen atoms were excluded as they had minimal impact on the fit quality. Due to the large number of degrees of freedom for the $P\bar{1}$ structure, restraints were implemented with the Distance_Restrain and Angle_Restrain functions in TOPAS to avoid overfitting the data and generating unphysical models. Certain bond distances and angles were restrained to ideal values, based on the ordered model from the SC-XRD solution, with a small tolerance to allow for deviations from the average structure. In the organic component, the ideal values for C–S, C–C, and C–N bond distances were set to 1.829 Å, 1.523 Å, and 1.495 Å, respectively, based on the SC-XRD solution and similar to previously reported values for cysteamine.[18] The ideal values for C–C–N and S–C–C angles were both set to 110º. Additional S–C–N angles were restrained, with ideal values of 98º or 147º, to model the S–C–C–N torsion angles as in the original model. To prevent unphysical distortions in the inorganic component, certain Cl–Sn–Cl, Cl–Sn–S, and S–Sn–S bond angles were restrained with a tolerance of 30º.

2.4 Diffuse reflectance spectroscopy

Diffuse reflectance measurements were conducted using a Shimadzu UV-2600 spectrometer equipped with an integrating sphere. The spectra were collected with a reflective backing of compressed $BaSO_4$ mixed with the samples. The measurements were conducted under ambient atmosphere. The spectra were converted to pseudo-absorbance spectra using the Kubelka–Munk transformation.[19]

2.5 Thin-film absorption

Thin-film absorption measurements were conducted using a Shimadzu UV-2600 spectrometer. The measurements were conducted under ambient atmosphere. A baseline correction was applied to account for light scattering from the films.

2.6 Photoluminescence spectroscopy

Photoluminescence spectra were acquired using a Horiba Jobin-Yvon Nanolog or Horiba Fluorolog-3 fluorimeter equipped with a 450-W xenon lamp and R928S detector (for visible-light range up to 775 nm), PPD-900 detector (for visible-light range up to 900 nm), or H10330B-75 detector (for infrared range). Crystal samples were prepared by securing as-synthesized crystals to AT205 Black Aluminum Foil Tape with a solution of PMMA in toluene. Powder samples were prepared by first grinding crystals in a mortar and pestle, followed by suspension in a solution of PMMA in toluene. This slurry was dropped onto AT205 Black Aluminum Foil Tape, then allowed to dry at room temperature. Samples were cooled using liquid nitrogen with a Janis ST-100 cryostat. A Lakeshore Autotune temperature controller equipped with a PID loop was used to set the temperature. Appropriate long-pass filters were used on the emission monochromator to filter out the scattered excitation. Stokes shifts were determined from the energy

difference between the peak of the emission spectrum and the peak of the excitation spectrum. The Jacobian correction was applied to all spectra while converting from wavelength to an energy scale.[20] Full-width at half-maximum values were calculated by fitting the spectra to a Gaussian.

2.7 Time-resolved photoluminescence

Solid samples for emission lifetime studies were placed in an NMR tube that was immersed into a quartz finger dewar that contained liquid N_2. Transient luminescence lifetime data were collected using an IBH Fluorocube instrument equipped with a Jobin Yvon detector and interfaced with a Horibia Fluorohub+ controller. A pulsed NanoLED N-330 (331 nm) excitation source was used for time-correlated single-photon counting (TCSPC) measurements of **Sn_control** and a SpectraLED S-03 (372 nm) was used for multi-channel scaling (MCS) measurements of **Sn_RCh**. Data analysis was performed using a Horiba DAS6 software package. Typically, data from the first 1–5 microseconds of the transient decay from MCS measurements were excluded from the fits owing to scattering artifacts from the sample.

2.8 Linearly polarized photoluminescence spectroscopy

Single crystals of **Sn_CYS**, (HIS)$PbBr_4$, and $(PEA)_2SnI_4$ were secured to silicon substrates with a solution of PMMA in toluene. Smaller fragments of large crystals of **Sn_CYS** were selected to avoid twin boundaries. Optical microscopy images of crystals were collected using a Nikon LV100ND microscope with a 5X objective and DS-Ri2 camera. Linearly polarized photoluminescence measurements were conducted at 80 K using a Coherent® OBIS 377 nm LX 50 mW laser. Similar to a setup reported by Pan et al.,[21] the laser beam propagated through a neutral-density filter (Thorlabs NDC-50C-4M), a half-wave plate (Newport 10RP42-2), a linear polarizer (Union Optic PGT5012), and a quarter-wave plate (Union Optic WSA4220-380-1100-M25.4) to generate vertically polarized light. The beam was then focused through an objective (Olympus MPLFLN50x NA 0.8) installed in a CryoAdvance 100 (Montana Instruments). The PL was collected through the same objective and passed through a 50:50 beamsplitter (Thorlabs BPD254), a long-pass filter (Edmund Optics 550 nm, #62-984), two different achromatic doublet lenses (f1 = 200 mm and f2 = 150 mm), then a Wollaston prism (Thorlabs WPM10). The horizontally and vertically polarized emissions were then spatially separated, and the separated emissions were focused through a third achromatic doublet lens (f3 = 100 mm) onto the entrance slit of a spectrophotometer (Princeton Instruments Acton SP2500) and then detected simultaneously by a liquid-N_2 cooled Si CCD camera (Princeton Instruments PyLon 400B eXcelon). The system was calibrated using an incandescent lightbulb as an unpolarized light source to account for the intrinsic polarization bias of the optical components in the emission path (see Figure S27). Spectra were integrated between 554–905 nm to measure the total photoluminescence intensity. Artifacts from cosmic rays and from a dead pixel in the detector at 873.6 nm were removed from the spectra.

For measurements of the emission intensity as a function of emission polarization angle, a rotatable linear polarizer (LPNIR100-MP2) was inserted in the emission path after the long-pass filter, and the total emission intensity was recorded at each angle.

Reflectance measurements were conducted at 80 K using a vertically polarized 730 nm laser (CNI Lasers MDL-III-730L-30mW) propagating through the same path as the photoluminescence measurements. A 730 nm notch filter (Chroma ZET730NF) was added to avoid saturating the CCD camera.

2.9 Power-dependent photoluminescence spectroscopy

Power-dependent photoluminescence was measured at 80 K on the same home-built setup used for the linearly polarized measurements. A crystal of **Sn_CYS** was secured to a fused silica substrate with a solution of PMMA in toluene. The beam was focused to a ca. 2 μm spot on a uniform section of the crystal to avoid twin boundaries. The incident power of the excitation was measured prior to entering the objective with a power meter (Thorlabs S130C), then scaled to account for the transmittance of the laser through the objective. The total photoluminescence intensity (integrated between 554–905 nm) was measured at each incident power.

2.10 Polarized microscopy

Images of single crystals under plane-polarized and cross-polarized light were collected using an Olympus SZX7 microscope equipped with a polarizer (Olympus SZX-PO), rotating analyzer (Olympus SZX2-AN), and camera (UCMOS03100KPA-U-NA-N-C-SQ-NA).

2.11 Raman spectroscopy

Raman spectra were collected using a Renishaw RM1000 Raman microscope equipped with a 633 nm laser. The samples were ground into powder and loaded on a clean glass substrate. The measurements were conducted under ambient atmosphere.

2.12 Stability tests

Thermogravimetric analyses (TGA) were conducted with a Netzsch TG 209 F1 Libra ThermoMicrobalance at a heating rate of 1 °C/minute from room temperature to 900 ºC under N_2 flow, using ca. 10–20 mg samples in alumina crucibles. The decomposition temperature (T_d) is defined as the temperature corresponding to 5% mass loss. TGA analyses (Figure S9) indicated decomposition temperatures of 214 ºC and 197 ºC for **Sn_CYS** and **Sn_SeCYS**, respectively, comparable to the decomposition temperature of **Sn_control** (202 ºC).

To study oxygen stability, powders were placed on clean glass slides and transferred to a glass jar containing Drierite desiccant. The jar was put inside a drawer to minimize light exposure. The stability of the powders was monitored intermittently by PXRD.

To study humidity stability, powders were placed on clean glass slides and transferred to a platform inside a desiccator cabinet with gas ports. To maintain the relative humidity (RH) at approximately 60% in the absence of oxygen, a constant flow of N_2 gas through a bubbler containing saturated NaBr aqueous solution was maintained.[22] The chamber was kept in the dark to minimize light exposure. The stability of the powders was monitored intermittently by PXRD.

The **Sn_RCh** heterostructures could be handled under ambient conditions. PXRD patterns of **Sn_CYS** and **Sn_SeCYS** showed no changes after 60 days of exposure to dry air (Figure S8), matching the stability of **Sn_control**. After 3–5 days exposure to N_2 at ca. 60% relative humidity, PXRD patterns of **Sn_CYS** and **Sn_SeCYS** showed small additional peaks, but the structures remained largely unchanged after 60 days of humidity exposure. Likewise, PXRD patterns of **Sn_control** showed no evidence of decomposition after 60 days of exposure to N_2 at ca. 60% relative humidity.

2.13 Strain calculations

Strain calculations were performed according to a previously reported procedure from our group,[3] using the online software suite ISOTROPY and component programs ISOCIF and ISODISTORT.[23,24] Sublattice strain tensors for both the perovskite and the intergrowth sheets in the single-crystal structures were determined relative to previously reported structures of the parent sublattices in materials with three-dimensional connectivity.[3] For each perovskite sublattice, a primitive cell was constructed with ISOCIF using terminal halides of the perovskite slab as lattice points (Figure S10). For each intergrowth sublattice, a primitive cell was constructed with ISOCIF using metal atoms in the intergrowth slab (Figure S10). Engineering strain tensors were calculated using the derived primitive cells and the ISODISTORT program. Relative linear, areal, and volumetric strains were calculated from the strain tensor, ε, as:

$$\varepsilon = \begin{bmatrix} \varepsilon_{xx} & \frac{\gamma_{xy}}{2} & \frac{\gamma_{xz}}{2} \\ \frac{\gamma_{xy}}{2} & \varepsilon_{yy} & \frac{\gamma_{yz}}{2} \\ \frac{\gamma_{xz}}{2} & \frac{\gamma_{yz}}{2} & \varepsilon_{zz} \end{bmatrix}$$

where x, y and z correspond to the axes of the primitive cell, and γ_{xy}, γ_{xz}, and γ_{yz} are the respective engineering shear strains. The fractional change in dimension is reported as $|\varepsilon + I|$ for the corresponding submatrix where I is the identity matrix.

2.14 Computational methodology

Density functional theory (DFT) calculations[25,26] were carried out using the Quantum Espresso-6.5 package.[27,28] Within this implementation, the core electron contributions of different chemical elements are described by the Optimised Norm Conserving Vanderbilt pseudopotentials (ONCV),[29] for the Perdew-Burke-Ernzerhof GGA exchange correlation functional[30] in the Pseudo-Dojo database.[31] We used a kinetic energy cut-off of 70 Ry with *k*-mesh of 6 × 4 × 4 for all structural relaxations and calculations.

We used fully relativistic pseudopotentials to include spin-orbit coupling (SOC) effects in all calculations of the electronic band structure. The elemental contributions are grouped into two categories: (a) perovskite: atoms spatially localized within the perovskite layer, and (b) non-perovskite: atoms spatially localized within the non-perovskite (intergrowth) layer. Contributions from halogens situated at the interface of perovskite and non-perovskite layer are shared with a ratio of 50:50 between the two layers.

To obtain the initial structural models suitable for electronic structure calculations, we constructed simplified ordered models of **Sn_CYS** and **Sn_SeCYS** based on the $P\bar{1}$ structures obtained at 100 K from SC-XRD. We note that the SC-XRD structures of **Sn_RCh** obtained at 100 K and at 300 K are very similar, with only slight differences in bond distances and angles at different temperatures. These solutions showed disordered occupation of sites in the perovskite layer and in the organic ligand, with a majority occupation of 76–80% and a minority occupation of 20–24%. The disordered Sn, Cl, C, and N atoms were fixed to occupy only the majority occupation sites, and the minority occupation sites were removed. Further, only the H atoms in the lattice were relaxed to a minimal force of 0.0001 Ry/Bohr, keeping the lattice constants and atomic/Wyckoff positions of the rest of the atoms fixed. To create the hypothetical structure **Pb@Sn_CYS**, all Sn atoms in the ordered model of **Sn_CYS** (with only H atoms relaxed) were replaced by Pb, and all atomic positions were unchanged. The structures thus obtained are reported as supplementary data for reproducibility.

Table S1. Crystallographic Data for $(Sn_2Cl_2)(CYS)_2SnCl_4$ (**Sn_CYS**)

Empirical formula	$C_4H_{14}Cl_6N_2S_2Sn_3$	$C_4H_{14}Cl_6N_2S_2Sn_3$	$C_4H_{14}Cl_6N_2S_2Sn_3$	$C_4H_{14}Cl_6N_2S_2Sn_3$
Formula weight (g/mol)	723.06	723.06	723.06	723.06
Temperature (K)	100	80	100	300
Crystal System	monoclinic	monoclinic	triclinic	triclinic
Space Group	$C2/c$	$C2/c$	$P\bar{1}$	$P\bar{1}$
a (Å)	28.000(4)	27.991(1)	10.6756(4)	10.7568(3)
b (Å)	6.1906(9)	6.1783(3)	12.3621(6)	12.4632(3)
c (Å)	10.674(2)	10.6622(5)	14.3245(7)	14.3926(5)
α (°)	90	90	102.428(2)	102.503(1)
β (°)	105.578(4)	105.497(2)	105.161(2)	105.037(1)
γ (°)	90	90	90.031(2)	90.006(1)
Volume (Å^3)	1782.2(5)	1776.9(2)	1778.7(1)	1816.03(9)
Z	4	4	4	4
Density, calc. (g/cm^3)	2.695	2.703	2.700	2.645
Absorption coeff. (mm^{-1})	5.588	5.231	5.226	5.118
F(000)	1344	1344	1344	1344
Crystal size (mm^3)	0.10 × 0.07 × 0.01	0.09 × 0.09 × 0.01	0.15 × 0.09 × 0.01	0.11 × 0.09 × 0.01
Radiation	Synchrotron (λ = 0.7288 Å)	Mo Kα (λ = 0.71073 Å)	Mo Kα (λ = 0.71073 Å)	Mo Kα (λ = 0.71073 Å)
2θ range (°)	6.196 to 55.720	6.042 to 54.248	3.960 to 52.824	3.970 to 52.818
Index ranges	$-35 \le h \le 35$, $-7 \le k \le 7$, $-13 \le l \le 13$	$-35 \le h \le 35$, $-7 \le k \le 6$, $-13 \le l \le 13$	$-13 \le h \le 13$, $-15 \le k \le 15$, $-17 \le l \le 17$	$-13 \le h \le 13$, $-15 \le k \le 15$, $-17 \le l \le 17$
Reflections collected/unique	14467/1960	8895/1958	58105/7297	47112/7433
Completeness to θ_{max}	0.999	0.999	0.999	0.996
Max. and min. transmission	0.946/0.512	0.950/0.662	0.950/0.720	0.951/0.668
Data/restraints/parameters	1960/0/120	1958/0/120	7297/4/459	7433/9/477
Goodness-of-fit on F^2	1.045	1.030	1.107	1.077
Final R indices [$I > 2\sigma(I)$]^a	R_1 = 0.0193 wR_2 = 0.0473	R_1 = 0.0218 wR_2 = 0.0502	R_1 = 0.0259 wR_2 = 0.0555	R_1 = 0.0335 wR_2 = 0.0661
R indices (all data)^a	R_1 = 0.0216 wR_2 = 0.0485	R_1 = 0.0247 wR_2 = 0.0518	R_1 = 0.0321 wR_2 = 0.0586	R_1 = 0.0471 wR_2 = 0.0734
Largest diff. peak/hole (e/Å^3)	0.446/–0.717	0.705/–0.651	0.830/–1.090	0.658/–0.883

$^aR_1 = \Sigma||F_o| - |F_c||/\Sigma|F_o|$, w$R_2 = [\Sigma w(F_o^2 - F_c^2)^2/\Sigma(F_o^2)^2]^{1/2}$

Table S2. Crystallographic Data for $(Sn_2Cl_2)(SeCYS)_2SnCl_4$ (**Sn_SeCYS**)

Empirical formula	$C_4H_{14}Cl_6N_2Se_2Sn_3$	$C_4H_{14}Cl_6N_2Se_2Sn_3$	$C_4H_{14}Cl_6N_2Se_2Sn_3$
Formula weight (g/mol)	816.86	816.86	816.86
Temperature (K)	100	100	300
Crystal system	monoclinic	triclinic	triclinic
Space group	*C2/c*	$P\bar{1}$	$P\bar{1}$
a (Å)	28.347(3)	10.7085(4)	10.7869(4)
b (Å)	6.2482(5)	12.4728(6)	12.5760(4)
c (Å)	10.7189(8)	14.4943(7)	14.5438(5)
α (°)	90	77.595(2)	77.520(1)
β (°)	105.819(2)	74.603(2)	74.865(1)
γ (°)	90	89.996(2)	89.989(1)
Volume (Å^3)	1826.6(3)	1819.5(1)	1856.2(1)
Z	4	4	4
Density, calc. (g/cm^3)	2.970	2.982	2.923
Absorption coeff. (mm^{-1})	8.841	8.875	8.700
F(000)	1488	1488	1488
Crystal size (mm^3)	0.13 × 0.06 × 0.02	0.09 × 0.07 × 0.01	0.13 × 0.11 × 0.01
Radiation	Mo Kα (λ = 0.71073 Å)	Mo Kα (λ = 0.71073 Å)	Mo Kα (λ = 0.71073 Å)
2θ range (°)	5.976 to 52.756	3.952 to 52.848	3.918 to 52.824
Index ranges	$-35 \le h \le 35$, $-7 \le k \le 7$, $-13 \le l \le 13$	$-13 \le h \le 13$, $-15 \le k \le 15$, $-18 \le l \le 18$	$-13 \le h \le 13$, $-15 \le k \le 15$, $-18 \le l \le 18$
Reflections collected/unique	16870/1865	65071/7472	35036/7635
Completeness to θ_{max}	0.995	0.998	0.998
Max. and min. transmission	0.843/0.647	0.916/0.695	0.918/0.585
Data/restraints/parameters	1865/0/120	7472/366/435	7635/0/477
Goodness-of-fit on F^2	1.174	1.178	1.064
Final R indices [$I > 2\sigma(I)$]^a	R_1 = 0.0102, wR_2 = 0.0221	R_1 = 0.0385, wR_2 = 0.0797	R_1 = 0.0346, wR_2 = 0.0725
R indices (all data)^a	R_1 = 0.0103, wR_2 = 0.0221	R_1 = 0.0448, wR_2 = 0.0826	R_1 = 0.0595, wR_2 = 0.0845
Largest diff. peak/hole (e/Å^3)	0.281/–0.495	1.758/–1.791	0.675/–0.722

$^aR_1 = \Sigma||F_o| - |F_c||/\Sigma|F_o|$, $wR_2 = [\Sigma w(F_o^2 - F_c^2)^2/\Sigma(F_o^2)^2]^{1/2}$

Table S3. Crystallographic Data for $(T4YMA)_2SnCl_4$ (**Sn_control**)

Empirical formula	$C_{12}H_{28}Cl_4N_2S_2Sn$	$C_{12}H_{28}Cl_4N_2S_2Sn$
Formula weight (g/mol)	524.97	524.97
Temperature (K)	100	300
Crystal system	triclinic	triclinic
Space group	$P\bar{1}$	$P\bar{1}$
a (Å)	11.1092(6)	11.2163(3)
b (Å)	11.1432(6)	11.2455(3)
c (Å)	16.554(1)	16.7472(5)
α (°)	95.364(2)	95.713(1)
β (°)	94.388(2)	94.889(1)
γ (°)	90.413(2)	90.587(1)
Volume (Å^3)	2034.1(2)	2093.9(1)
Z	4	4
Density, calc. (g/cm^3)	1.714	1.665
Absorption coeff. (mm^{-1})	1.956	1.900
F(000)	1056	1056
Crystal size (mm^3)	0.13 × 0.11 × 0.01	0.13 × 0.11 × 0.01
Radiation	Mo Kα (λ = 0.71073 Å)	Mo Kα (λ = 0.71073 Å)
2θ range (°)	4.232 to 54.274	4.180 to 54.282
Index ranges	$-14 \leq h \leq 14$, $-14 \leq k \leq 14$, $-21 \leq l \leq 21$	$-14 \leq h \leq 14$, $-14 \leq k \leq 14$, $-21 \leq l \leq 21$
Reflections collected/unique	73780/8992	55480/9288
Completeness to θ_{max}	0.998	0.998
Max. and min. transmission	0.981/0.699	0.981/0.672
Data/restraints/parameters	8992/0/411	9288/0/411
Goodness-of-fit on F^2	1.061	1.046
Final R indices [$I > 2\sigma(I)$][a]	R_1 = 0.0214, wR_2 = 0.0530	R_1 = 0.0275, wR_2 = 0.0654
R indices (all data)[a]	R_1 = 0.0238, wR_2 = 0.0545	R_1 = 0.0362, wR_2 = 0.0716
Largest diff. peak/hole (e/Å^3)	0.457/–0.592	0.715/–0.578

[a]$R_1 = \Sigma||F_o| - |F_c||/\Sigma|F_o|$, $wR_2 = [\Sigma w(F_o^2 - F_c^2)^2/\Sigma(F_o^2)^2]^{1/2}$

Table S4. Crystallographic Data for $(Sn_{1.85}Pb_{0.15}Cl_2)(CYS)_2Sn_{0.69}Pb_{0.31}Cl_4$ (**Sn_CYS**:**Pb**) and $(Sn_{1.68}Pb_{0.32}Cl_2)(SeCYS)_2Sn_{0.80}Pb_{0.20}Cl_4$ (**Sn_SeCYS**:**Pb**)

Empirical formula	$C_4H_{14}Cl_6N_2S_2Sn_{2.54}Pb_{0.46}$	$C_4H_{14}Cl_6N_2Se_2Sn_{2.48}Pb_{0.52}$
Formula weight (g/mol)	763.55	862.66
Temperature (K)	100	100
Crystal system	monoclinic	monoclinic
Space group	*C*2/*c*	*C*2/*c*
a (Å)	28.082(2)	28.418(1)
b (Å)	6.1683(3)	6.2338(2)
c (Å)	10.6800(7)	10.7232(4)
α (°)	90	90
β (°)	105.457(2)	105.699(1)
γ (°)	90	90
Volume (Å^3)	1783.1(2)	1828.8(1)
Z	4	4
Density, calc. (g/cm^3)	2.844	3.133
Absorption coeff. (mm^{-1})	8.865	12.859
F(000)	1403	1554
Crystal size (mm^3)	0.09 × 0.08 × 0.02	0.07 × 0.05 × 0.02
Radiation	Mo Kα (λ = 0.71073 Å)	Mo Kα (λ = 0.71073 Å)
2θ range (°)	6.776 to 54.220	6.704 to 52.806
Index ranges	$-35 \le h \le 34$,<br>$0 \le k \le 7$,<br>$0 \le l \le 13$	$-35 \le h \le 35$,<br>$-7 \le k \le 7$,<br>$-13 \le l \le 13$
Reflections collected/unique	13163/1955	15764/1877
Completeness to θ_{max}	0.995	0.998
Max. and min. transmission	0.843/0.408	0.783/0.531
Data/restraints/parameters	1955/1/123	1877/0/122
Goodness-of-fit on F^2	1.185	1.347
Final *R* indices [$I > 2\sigma(I)$][a]	R_1 = 0.0345<br>wR_2 = 0.0905	R_1 = 0.0195<br>wR_2 = 0.0508
R indices (all data)[a]	R_1 = 0.0349<br>wR_2 = 0.0907	R_1 = 0.0199<br>wR_2 = 0.0509
Largest diff. peak/hole (e/Å^3)	1.561/–1.677	0.585/–0.722

[a] $R_1 = \Sigma||F_o| - |F_c||/\Sigma|F_o|$, $wR_2 = [\Sigma w(F_o^2 - F_c^2)^2/\Sigma(F_o^2)^2]^{1/2}$

Table S5. Crystallographic Data for $(Pb_2Cl_2)(CYS)_2PbCl_4$ (**Pb_CYS**)

Empirical formula	$C_4H_{14}Cl_6N_2S_2Pb_3$
Formula weight (g/mol)	988.56
Temperature (K)	300
Crystal system	orthorhombic
Space group	*Pnma*
a (Å)	7.4999(2)
b (Å)	26.7946(5)
c (Å)	8.8262(2)
α (°)	90
β (°)	90
γ (°)	90
Volume (Å^3)	1773.69(7)
Z	4
Density, calc. (g/cm^3)	3.702
Absorption coeff. (mm^{-1})	29.316
F(000)	1728
Crystal size (mm^3)	0.14 × 0.10 × 0.04
Radiation	Mo Kα (λ = 0.71073 Å)
2θ range (°)	4.860 to 54.220
Index ranges	$-9 \leq h \leq 8$, $-33 \leq k \leq 34$, $-11 \leq l \leq 10$
Reflections collected/unique	20229/1996
Completeness to θ_{max}	0.998
Max. and min. transmission	0.387/0.189
Data/restraints/parameters	1996/0/84
Goodness-of-fit on F^2	1.091
Final R indices [$I > 2\sigma(I)$]^a	$R_1 = 0.0196$ w$R_2 = 0.0473$
R indices (all data)^a	$R_1 = 0.0227$ w$R_2 = 0.0496$
Largest diff. peak/hole (e/Å^3)	0.802/–0.889

$^aR_1 = \Sigma||F_o| - |F_c||/\Sigma|F_o|$, $wR_2 = [\Sigma w(F_o^2 - F_c^2)^2/\Sigma(F_o^2)^2]^{1/2}$

Table S6. Octahedral distortion parameters and absorption onsets or bandgaps for **Sn_CYS**, **Sn_SeCYS**, and **Sn_control** at room temperature compared with previously reported 2D and 3D tin-chloride perovskites. For **Sn_control**, the position of the Sn atom at the center of the octahedra was averaged across two disordered positions. For structures with two crystallographically distinct octahedra, distortion parameters for both octahedra are shown. TMPDA = N,N,*N'*,*N'*-tetramethyl-1,4-phenylenediamine. The octahedral distortion parameters[32,33] are defined as:

$$\Delta_{oct} = \frac{1}{6}\sum_{n=1}^{6}\left[\frac{d_n - d_{avg}}{d_{avg}}\right]^2 \qquad \sigma^2_{oct} = \frac{1}{11}\sum_{m=1}^{12}(\theta_m - 90)^2$$

where n indexes each Sn–Cl bond in the octahedron, d_n is the distance of an individual Sn–Cl bond, d_{avg} is the average value of the Sn–Cl bond distances in the octahedron; m indexes each individual cis Cl–Sn–Cl bond angle, and θ_m is the value of the angle in degrees.

Composition	Δ_{oct} ($\times 10^{-2}$)[32]	σ^2_{oct}[33]	Absorption onset (eV)
**Sn_CYS**	2.23, 2.33	63.4, 62.5	2.94
**Sn_SeCYS**	2.46, 2.48	65.1, 65.1	2.65
**Sn_control**	0.881, 0.824	27.6, 26.2	3.28
(TMPDA)$SnCl_4$[34]	2.12	83.3	3.5 (indirect bandgap)[34]
$(CH_3NH_3)SnCl_3$, *P*1[35]	0.938, 1.04	72.3, 139.5	3.61 (direct bandgap)[36]

Table S7. Lattice strains estimated for the perovskite slabs of **Sn_CYS** and **Sn_SeCYS** relative to a 3D $(CH_3NH_3)SnCl_3$ parent structure in the *P*1 space group.[35] Here, *x* and *y* are in-plane axes (see Figure S10).

Heterostructure	Perovskite Parent	Strain ‖ *x*	Strain ‖ *y*	Strain ‖ *z*	Areal Strain	Volumetric Strain
**Sn_CYS**	$(CH_3NH_3)SnCl_3$	8.8%	–5.3%	–2.2%	3.0%	0.57%
**Sn_SeCYS**	$(CH_3NH_3)SnCl_3$	9.8%	–5.0%	–2.3%	4.2%	1.7%

Table S8. Lattice strains estimated for the intergrowth slabs of **Sn_CYS** and **Sn_SeCYS** relative to a 3D $SnCl_2$ parent structure.[37] Here, *x* and *y* are in-plane axes (see Figure S10).

Heterostructure	Intergrowth Parent	Strain ‖ *x*	Strain ‖ *y*	Strain ‖ *z*	Areal Strain	Volumetric Strain
**Sn_CYS**	$SnCl_2$	–20.1%	16.5%	–11.9%	–6.9%	–18.6%
**Sn_SeCYS**	$SnCl_2$	–19.3%	16.9%	–8.5%	–5.7%	–14.1%

Table S9. Lattice strains estimated for the perovskite and intergrowth slabs of **Pb_CYS** relative to a 3D $(CH_3NH_3)PbCl_3$ parent structure[38] and a 3D $PbCl_2$ parent structure,[39] respectively. Here, *x* and *y* are in-plane axes (see Figure S10).

Sublattice	Parent	Strain ‖ *x*	Strain ‖ *y*	Strain ‖ *z*	Areal Strain	Volumetric Strain
Perovskite	$(CH_3NH_3)PbCl_3$	–7.2%	9.2%	1.3%	1.4%	2.7%
Intergrowth	$PbCl_2$	–2.3%	–2.4%	–11.3%	–4.7%	–17.0%

Table S10. Relative strain tensors for the perovskite and intergrowth slabs of **Sn_RCh** and **Pb_CYS**.

Sublattice	ε_{xx}	ε_{yy}	ε_{zz}	ε_{yz}	ε_{xz}	ε_{xy}
**Sn_CYS**, perovskite	0.08763	–0.05284	–0.02246	0.01321	–0.026165	0.0231
**Sn_CYS**, intergrowth	–0.20072	0.16496	–0.11877	–0.088945	–0.023435	–0.00101
**Sn_SeCYS**, perovskite	0.09752	–0.05019	–0.02275	0.012935	–0.02514	0.023205
**Sn_SeCYS**, intergrowth	–0.19318	0.16935	–0.08455	–0.072785	–0.00961	–0.000445
**Pb_CYS**, perovskite	–0.0719	0.09219	0.01348	0	0	0
**Pb_CYS**, intergrowth	–0.02337	–0.02401	–0.11318	0.00543	–0.123305	0.00034

Table S11. Orbital contributions to the band edges for calculated band structures of **Sn_RCh**, **Pb_CYS**,[3] and the model structure **Pb@Sn_CYS**. The contributions of different elements to the VBT and CBB (in fraction) are evaluated at the Γ-point and the Z-point.

Band	Perovskite Layer		Intergrowth Layer				Bridging Cl
	Sn/Pb	Cl	Sn/Pb	Cl	S/Se	CNH	
**Sn_CYS**, VBT Γ-point	0.148	0.067	0.320	0.081	0.242	0.000	0.143
**Sn_CYS**, CBB Γ-point	0.005	0.000	0.763	0.099	0.085	0.047	0.000
**Sn_CYS**, VBT Z-point	0.142	0.065	0.315	0.099	0.233	0.004	0.142
**Sn_CYS**, CBB Z-point	0.014	0.000	0.760	0.105	0.076	0.044	0.000
**Sn_SeCYS**, VBT Γ-point	0.027	0.000	0.384	0.085	0.449	0.014	0.041
**Sn_SeCYS**, CBB Γ-point	0.009	0.000	0.764	0.074	0.116	0.037	0.000
**Sn_SeCYS**, VBT Z-point	0.027	0.000	0.387	0.086	0.432	0.014	0.054
**Sn_SeCYS**, CBB Z-point	0.014	0.000	0.755	0.076	0.109	0.045	0.000
**Pb_CYS**, VBT Γ-point	0.027	0.029	0.170	0.119	0.654	0.000	0.000
**Pb_CYS**, CBB Γ-point	0.802	0.092	0.053	0.000	0.000	0.000	0.053
**Pb_CYS**, VBT Z-point	0.032	0.068	0.163	0.099	0.615	0.004	0.018
**Pb_CYS**, CBB Z-point	0.795	0.100	0.052	0.000	0.000	0.000	0.052
**Pb@Sn_CYS**, VBT Γ-point	0.027	0.009	0.276	0.093	0.493	0.022	0.080
**Pb@Sn_CYS**, CBB Γ-point	0.108	0.000	0.679	0.066	0.141	0.000	0.005
**Pb@Sn_CYS**, VBT Z-point	0.031	0.013	0.269	0.097	0.476	0.022	0.092
**Pb@Sn_CYS**, CBB Z-point	0.104	0.000	0.692	0.062	0.142	0.000	0.000

Table S12. Time constants (τ) for the photoluminescence decay of **Sn_CYS**, **Sn_SeCYS** (372 nm excitation), and **Sn_control** (331 nm excitation) at 77 K.

Composition	Morphology	Emission wavelength (nm)	τ (μs)
**Sn_CYS**	Crystals	650	4.34(3)
**Sn_CYS**	Crystals	690	4.49(3)
**Sn_CYS**	Crystals	730	4.43(3)
**Sn_CYS**	Crystals	770	4.48(4)
**Sn_CYS**	Crystals	810	4.16(4)
**Sn_CYS**	Powder	730	4.78(1)
**Sn_SeCYS**	Crystals	700	3.73(4)
**Sn_SeCYS**	Crystals	740	3.69(4)
**Sn_SeCYS**	Crystals	780	3.71(7)
**Sn_SeCYS**	Crystals	820	3.72(5)
**Sn_SeCYS**	Powder	780	3.77(6)
**Sn_control**	Crystals	500	1.812(9)
**Sn_control**	Crystals	520	1.848(9)
**Sn_control**	Crystals	540	1.845(8)
**Sn_control**	Crystals	560	1.895(9)
**Sn_control**	Crystals	580	1.92(1)
**Sn_control**	Powder	540	1.887(9)

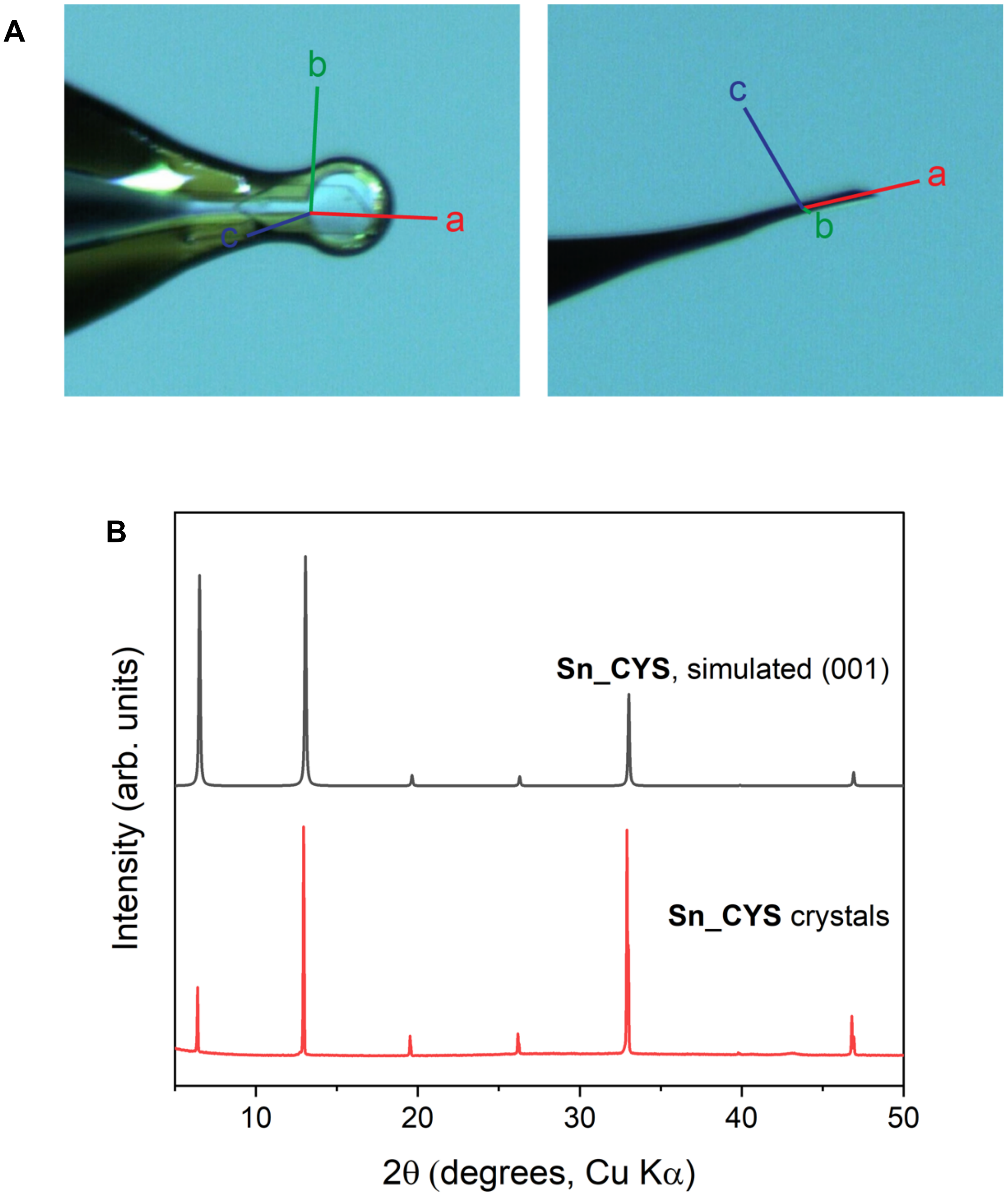

Figure S1. (A) Crystallographic axes of a single crystal of **Sn_CYS**, based on SC-XRD analysis of the unit cell. The *a*-axis in the $P\bar{1}$ structure aligns with the long axis of the crystal, and the *ab* plane is parallel to the flat surface of the plate-like crystals. (B) PXRD pattern of crystals of **Sn_CYS** mounted on a silicon substrate, confirming the (001) orientation of the crystals with the inorganic planes parallel to the substrate.

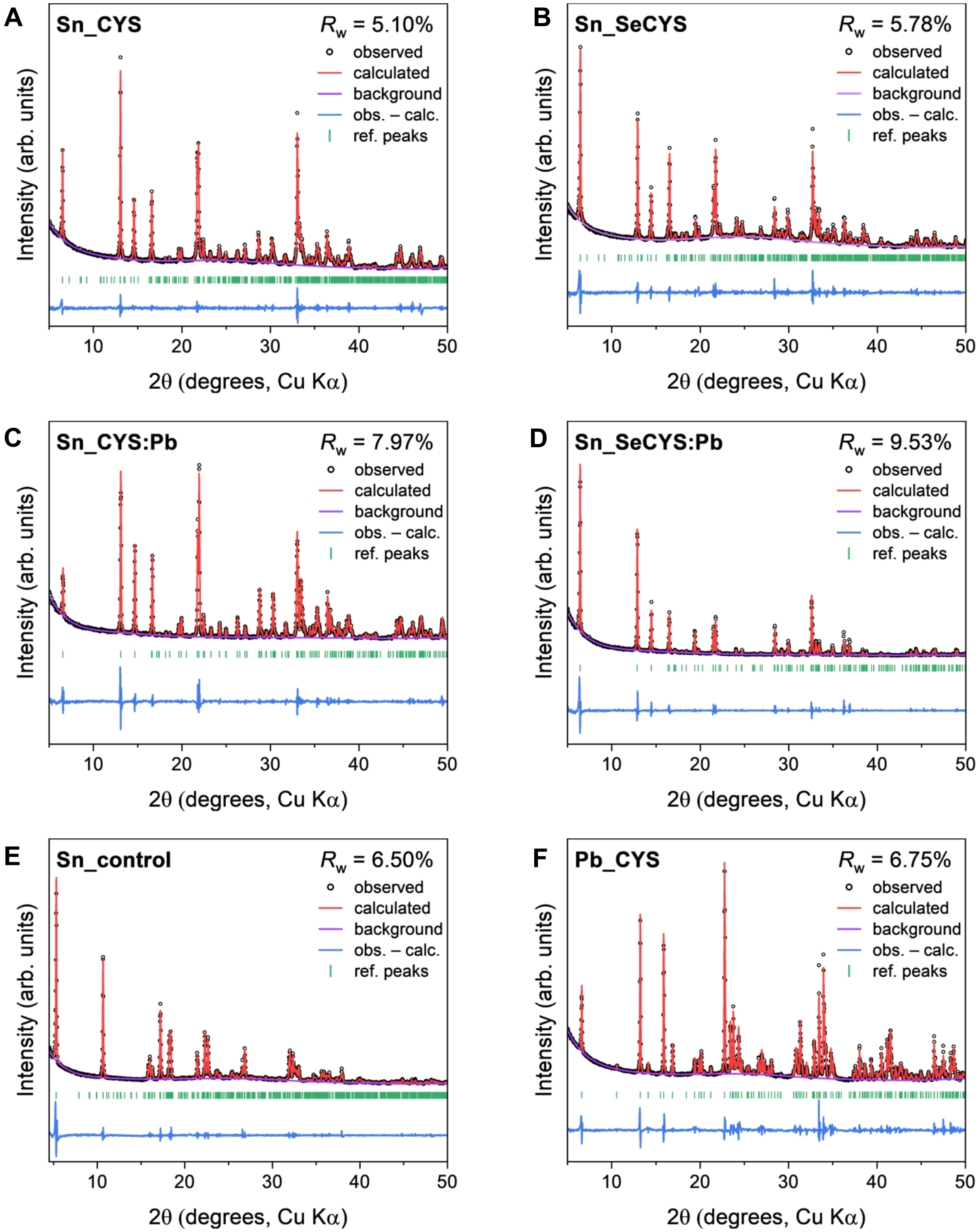

Figure S2. Pawley refinements for the powder X-ray diffraction patterns of (A) **Sn_CYS** (in $P\bar{1}$), (B) **Sn_SeCYS** (in $P\bar{1}$), (C) **Sn_CYS:Pb** (in $C2/c$), (D) **Sn_SeCYS:Pb** (in $C2/c$), (E) **Sn_control** (in $P\bar{1}$), and (F) **Pb_CYS** (in *Pnma*). Reflection positions are shown as green tick marks.

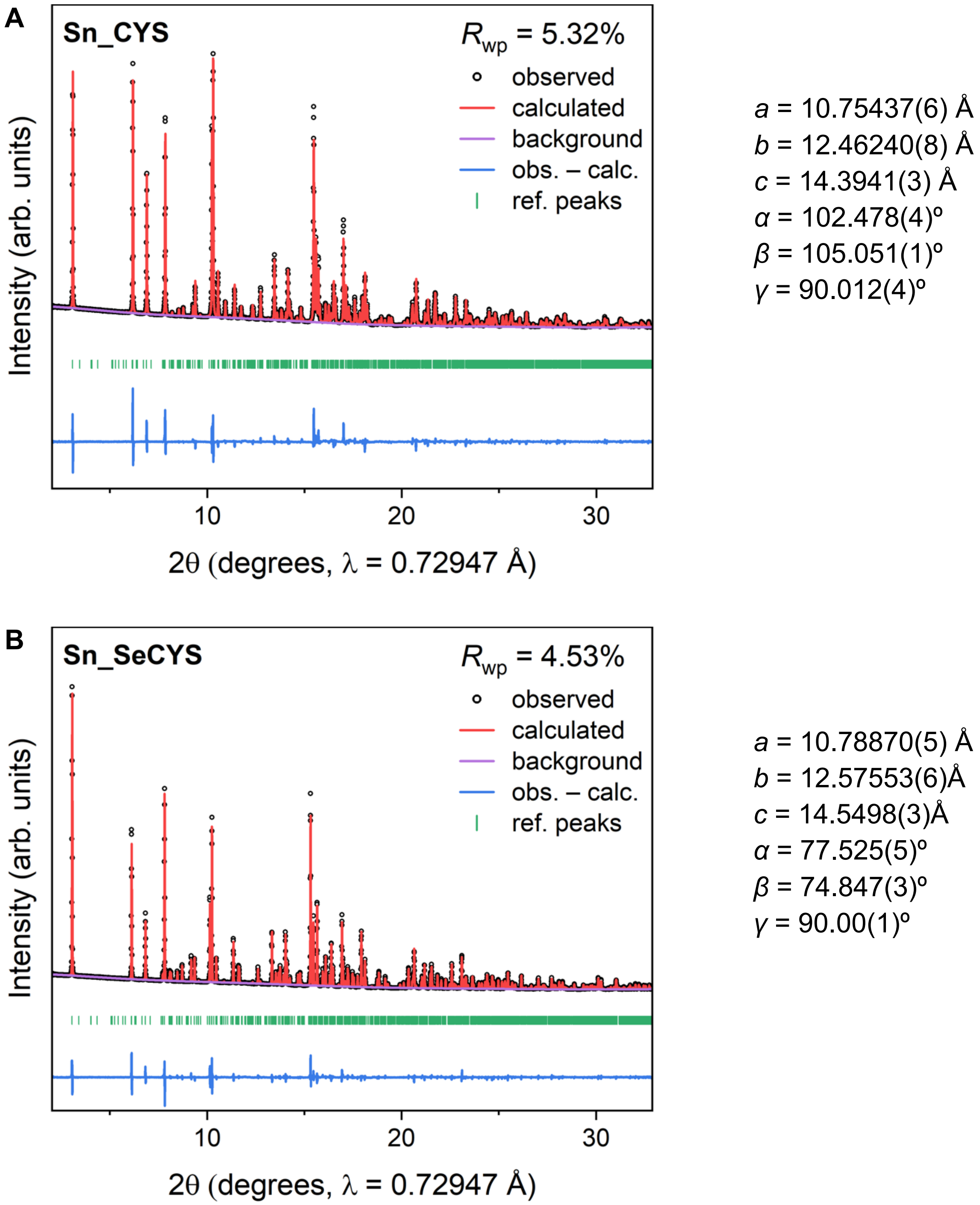

Figure S3. Rietveld refinements for synchrotron PXRD (λ = 0.72947 Å) patterns of (A) **Sn_CYS** and (B) **Sn_SeCYS**, both rinsed with dilute aqueous NaCl. Reflection positions are shown as green tick marks. The experimental powder patterns were refined against the $P\bar{1}$ structures from SC-XRD at 300 K. To confirm that the disordered structures obtained from SC-XRD are reasonable, only the lattice parameters were refined, and the atomic positions and thermal parameters were fixed. The good agreement of the SC-XRD atomic positions with the experimental data provides support for the reliability of the SC-XRD structures.

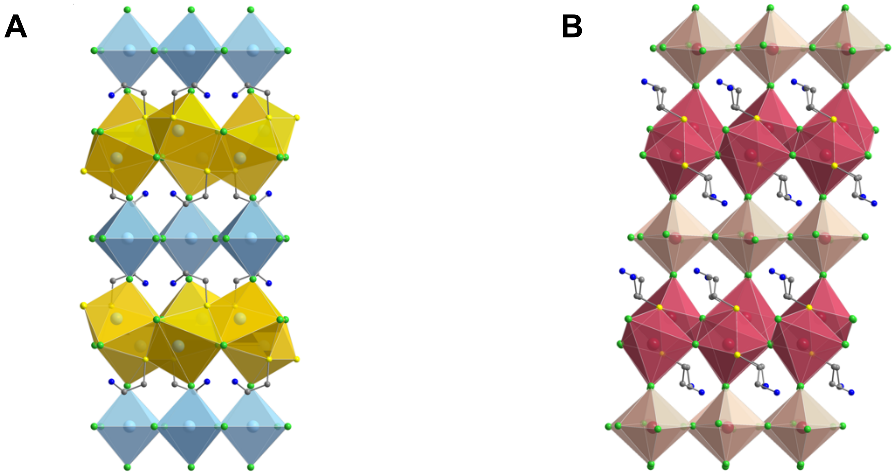

Figure S4. Comparison of the structures of (A) **Pb_CYS** and (B) **Sn_CYS**. Pb-coordination polyhedra in the perovskite and intergrowth layers are depicted in blue and yellow respectively, and Sn-coordination polyhedra in the perovskite and intergrowth layers are depicted in cream and dark red, respectively. Atom colors: Cl: green; S: yellow; N: blue; C: grey. Hydrogen atoms are omitted for clarity.

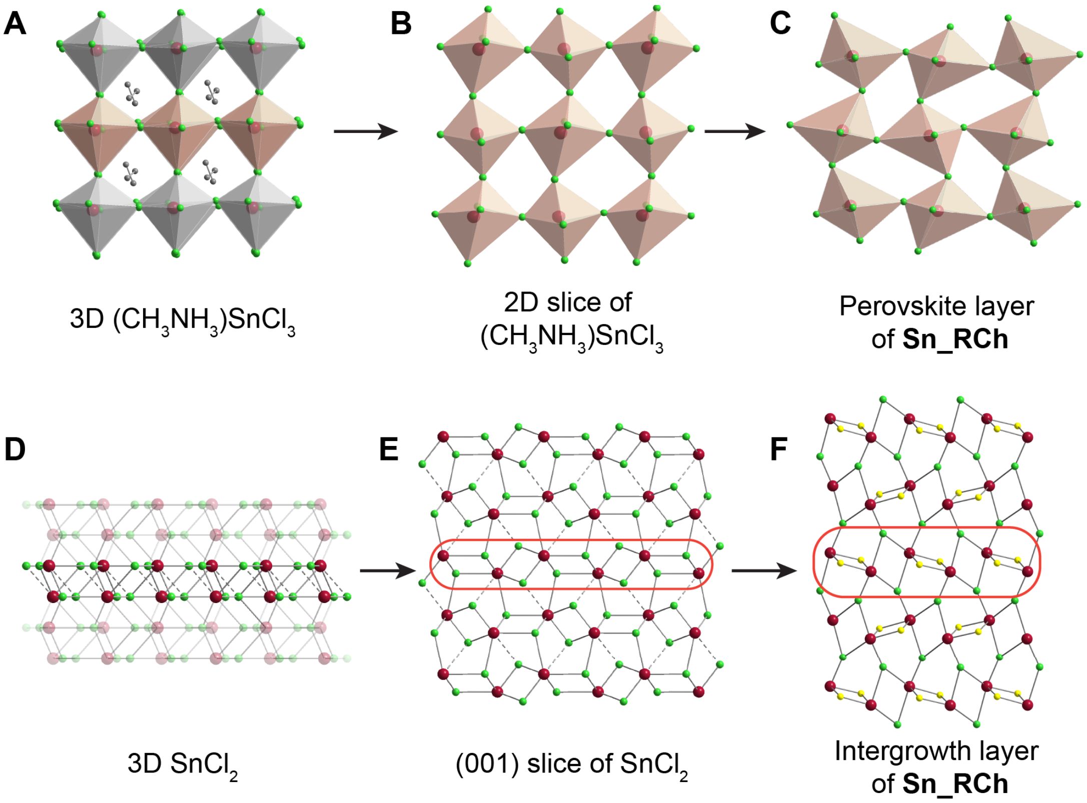

Figure S5. Schematic illustrating the conceptual derivation of the (C) perovskite and (F) intergrowth layers of **Sn_RCh** from the 3D parent structures (A, B) $CH_3NH_3SnCl_3$[35] and (D, E) $SnCl_2$,[37] respectively. The left panels (A, D) depict the 3D parent structures, with relevant 2D slices highlighted. The middle panels (B, E) depict a top-down view of each 2D slice. The right panels (C, F) depict the corresponding layers of the **Sn_RCh** heterostructure. The 1D chains of $[Sn_2Cl_2]$ dimers (in $SnCl_2$, E) and alternating $[Sn_2Cl_2]$ and $[Sn_2\mathit{Ch}_2]$ dimers (in the **Sn_RCh** intergrowth layer, F) are outlined in red. Atom colors: Sn: maroon; Cl: green; *Ch*: yellow. For $(CH_3NH_3)SnCl_3$, C and N atoms are shown in grey, and H atoms are omitted.

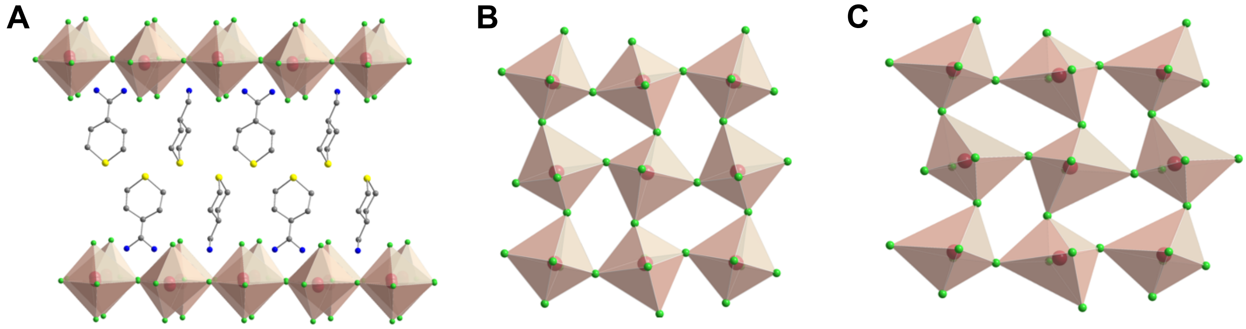

Figure S6. (A) Single-crystal X-ray diffraction structure of $(T4YMA)_2SnCl_4$ (**Sn_control**) at room temperature. In some of the organic molecules, the N atom is disordered across two positions. Hydrogen atoms are omitted for clarity. Comparison of (B) the perovskite layer of **Sn_control** and (C) the perovskite layer of an ordered model of **Sn_CYS**. Tin-chloride octahedra are shown in cream. Atom colors: Cl: green; S: yellow; N: blue; C: grey.

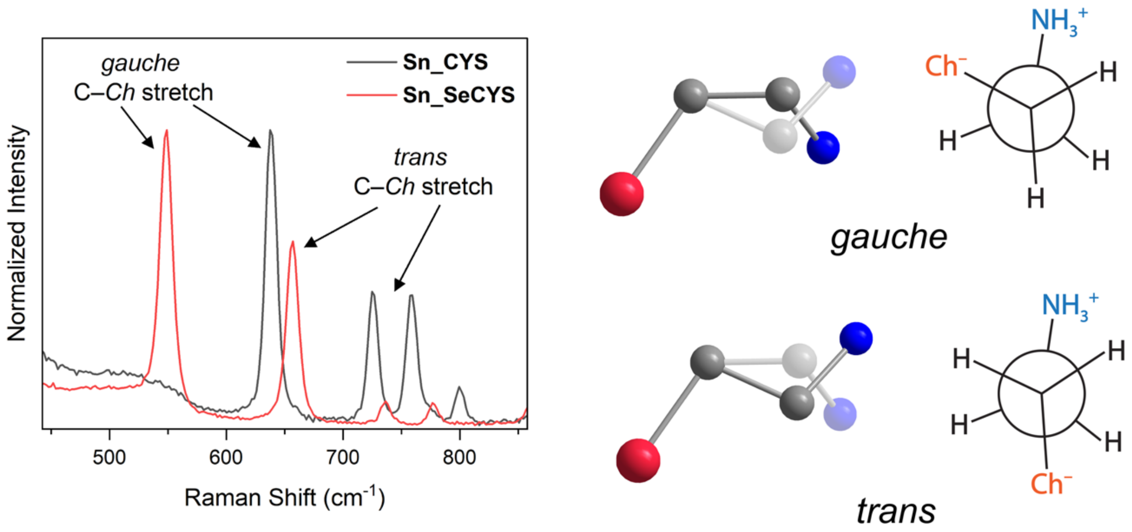

Figure S7. Raman signals (left) of *gauche* (top right) and *trans* (bottom right) C–S and C–Se stretches in **Sn_CYS** and **Sn_SeCYS**. Atom colors: *Ch*: red; N: blue; C: grey.

In **Sn_CYS**, we observed two distinct Raman bands at 638 and 725 cm^{-1}, which we assigned to *gauche* and *trans* C–S stretching modes, respectively. These assignments are consistent with reported stretches at 640 and 725 cm^{-1} for a CYS monolayer on silver.[40] The additional band at 759 cm^{-1} may correspond to an additional *trans* conformation with a slightly different dihedral angle that could not be distinguished by SC-XRD. The 3D perovskite $(CYS)PbCl_2$ similarly showed two *trans* C–S modes at 736 and 749 cm^{-1}, as well as a *gauche* C–S mode at 650 cm^{-1}.[1] In **Sn_SeCYS**, bands at 549 and 657 cm^{-1} were assigned to *gauche* and *trans* C–Se stretching modes, respectively, consistent with the C–Se stretches of the 3D perovskite $(SeCYS)PbCl_2$.[1] We estimated the relative ratios of the *gauche* and *trans* conformations (I_G/I_T) in **Sn_CYS** and **Sn_SeCYS** by integrating these peaks. The relative ratios of 1.2 (**Sn_CYS**) and 1.4 (**Sn_SeCYS**) corroborate the equal proportion of *gauche* and *trans* conformations indicated by the SC-XRD solutions.

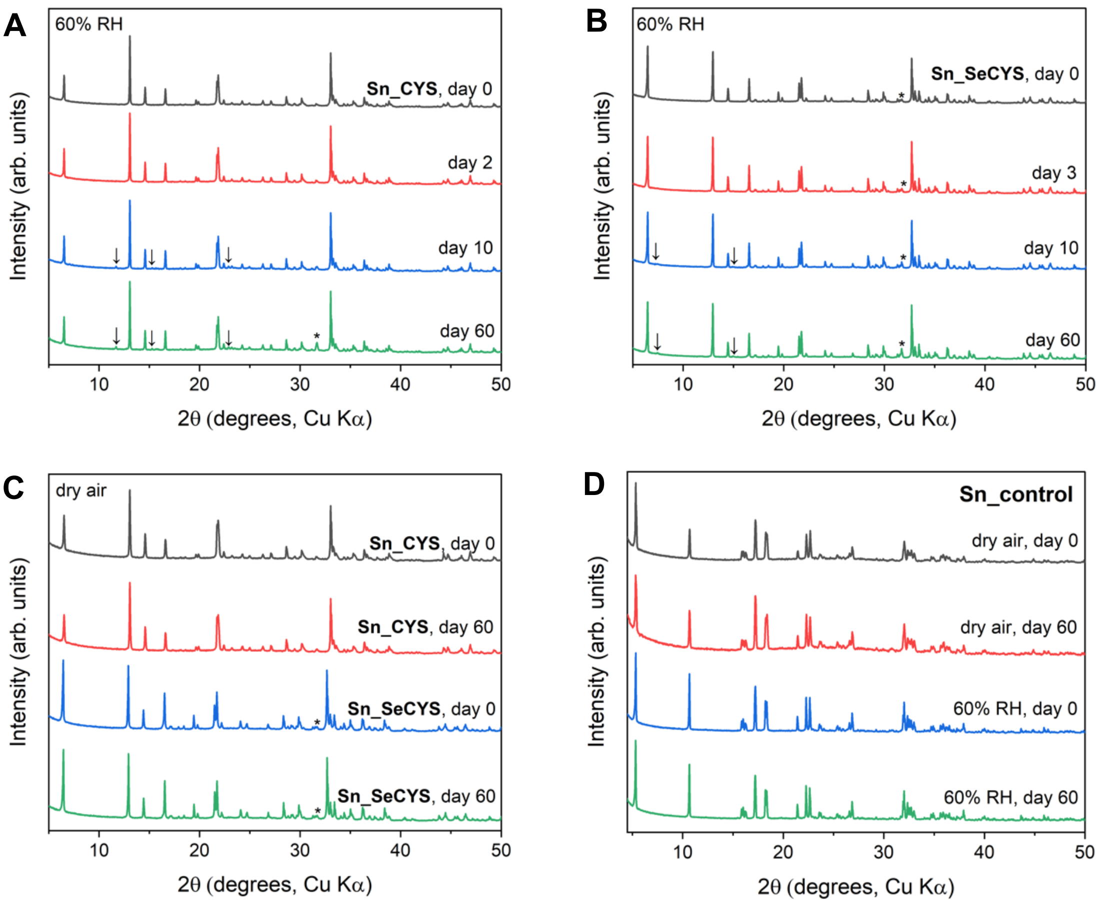

Figure S8. PXRD patterns of pulverized crystals of (A) **Sn_CYS** and (B) **Sn_SeCYS** exposed to N_2 at ca. 60% relative humidity. Downward arrows indicate small additional peaks indicating slight degradation after several days. The peak marked with an asterisk is from residual NaCl. (C) PXRD patterns of pulverized crystals of **Sn_CYS** and **Sn_SeCYS** exposed to oxygen in a jar containing desiccant. (D) PXRD patterns of pulverized crystals of **Sn_control** exposed to N_2 at ca. 60% relative humidity and exposed to oxygen in a jar containing desiccant.

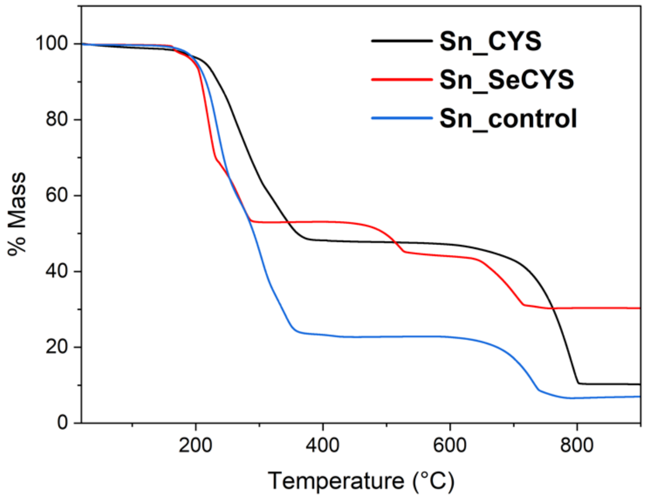

Figure S9. Thermogravimetric analysis of **Sn_CYS**, **Sn_SeCYS**, and **Sn_control** at a scan rate of 1 °C/minute, measuring from room temperature to 900 °C.

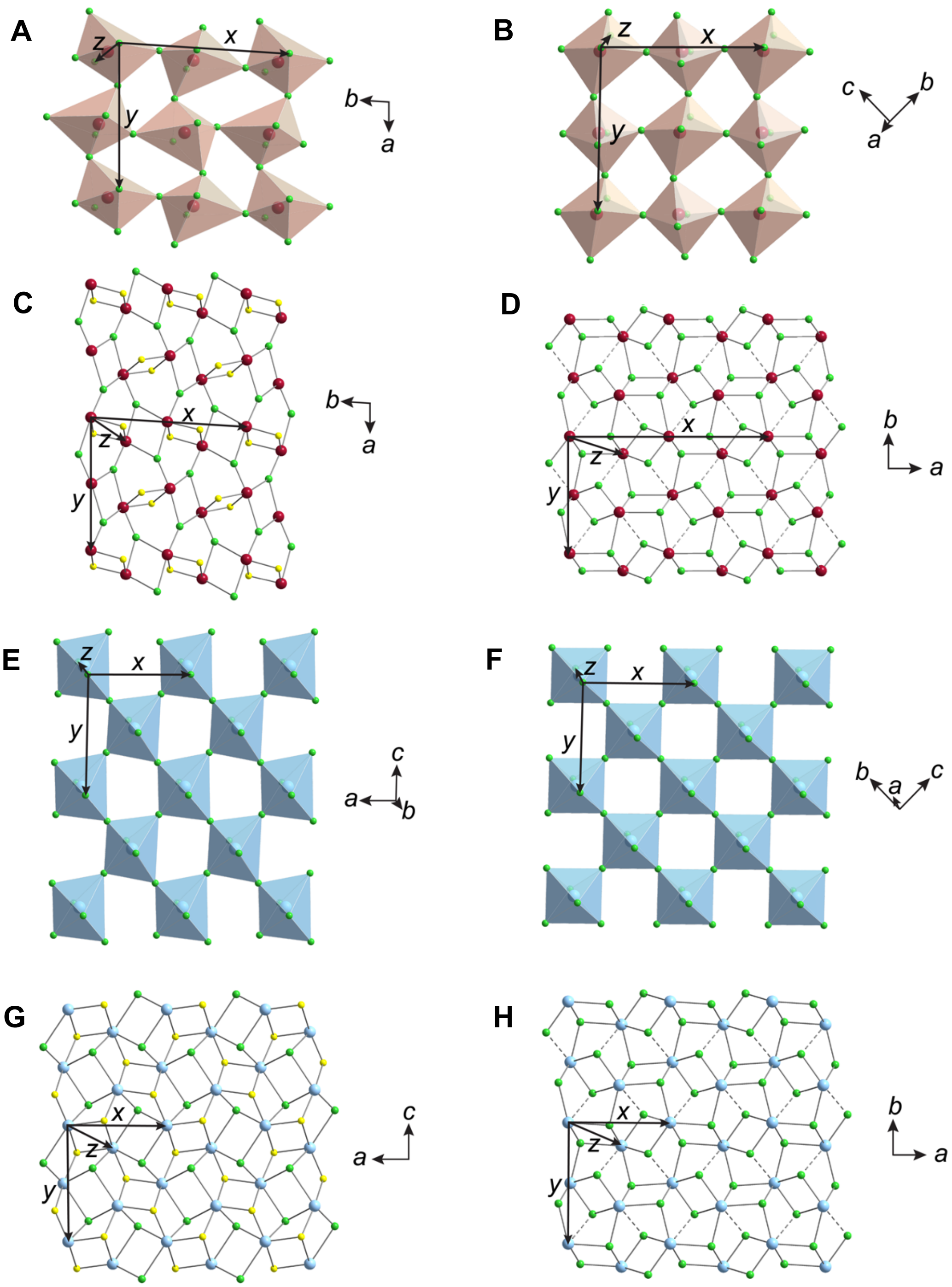

Figure S10. Cells used for estimation of lattice strain in **Sn_RCh** ($P\bar{1}$ model**)** and **Pb_CYS**. Crystallographic axes are specified as *a, b,* and *c*, and arbitrary axes for the cells of the sublattices are specified as *x, y,* and *z*. (A) Perovskite sublattice of **Sn_CYS**; (B) sublattice of $(CH_3NH_3)SnCl_3$;[35] (C) intergrowth sublattice of **Sn_CYS**; (D) sublattice of $SnCl_2$;[37] (E) Perovskite sublattice of **Pb_CYS**; (F) sublattice of $(CH_3NH_3)PbCl_3$;[38] (G) intergrowth sublattice of **Pb_CYS**; (H) sublattice of $PbCl_2$.[39] Whereas the intergrowth sublattice of **Pb_CYS** is isostructural to a (001) slice of $PbCl_2$, the difference in twist angle and the lattice-matching condition imposes an additional distortion in the intergrowth sublattice of **Sn_CYS**, where every other chain of corner-sharing dimers is "flipped" (see Figure 2). Atom colors: Pb: turquoise; Sn: maroon; Cl: green; S: yellow.

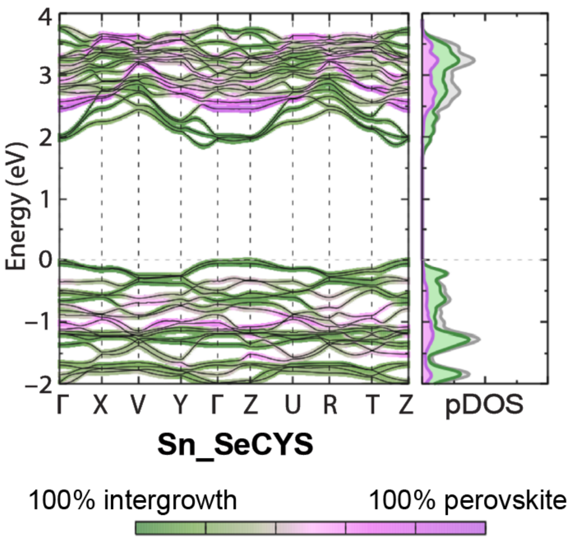

Figure S11. Electronic band structure (left) and projected density of states (pDOS, right) of **Sn_SeCYS**, along the high symmetry path Γ(0,0,0) – X(0.5,0,0) – V(0.5,0.5,0) – Y(0,0.5,0) – Γ(0,0,0) – Z(0,0,0.5) – U(0.5,0,0.5) – R(0.5,0.5,0.5) – T(0,0.5,0.5) – Z(0,0,0.5). The color gradient shows the relative perovskite (purple) and intergrowth (green) contributions to the electronic band structures. The energy of the valence band maxima has been arbitrarily set to 0 eV. The Γ→Z direction corresponds to the interlayer stacking direction. Color scheme for the pDOS: perovskite: purple; intergrowth: green; total: grey.

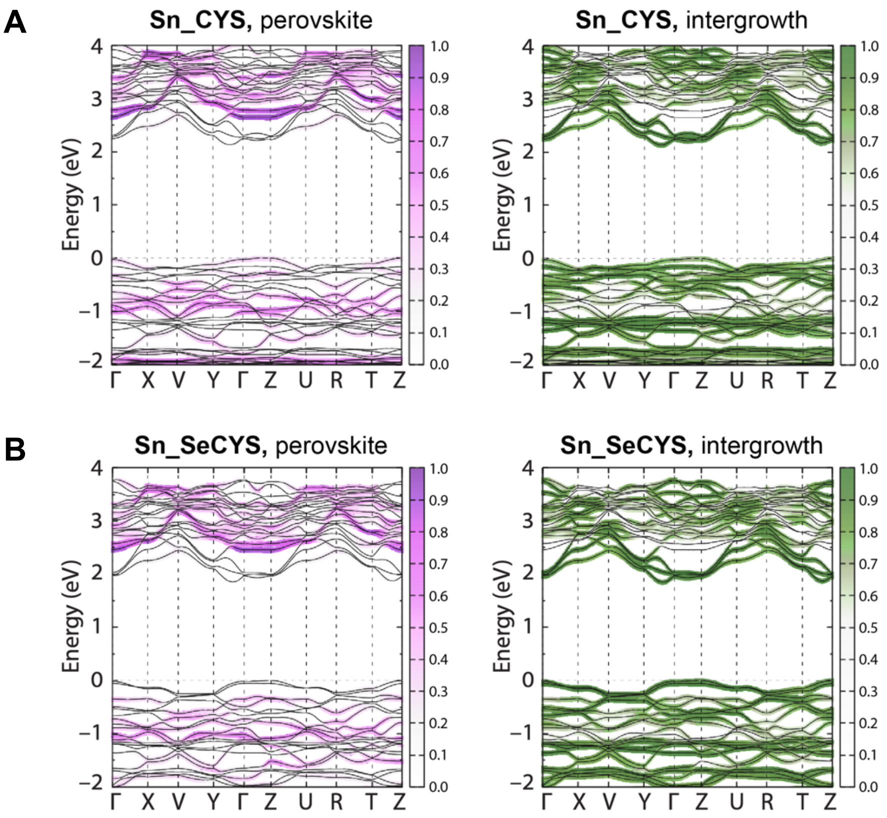

Figure S12. Contributions of the intergrowth layer (green) and the perovskite layer (purple) to the electronic band structures of (A) **Sn_CYS** and (B) **Sn_SeCYS**. The color gradient indicates the relative contribution of each layer to the band composition.

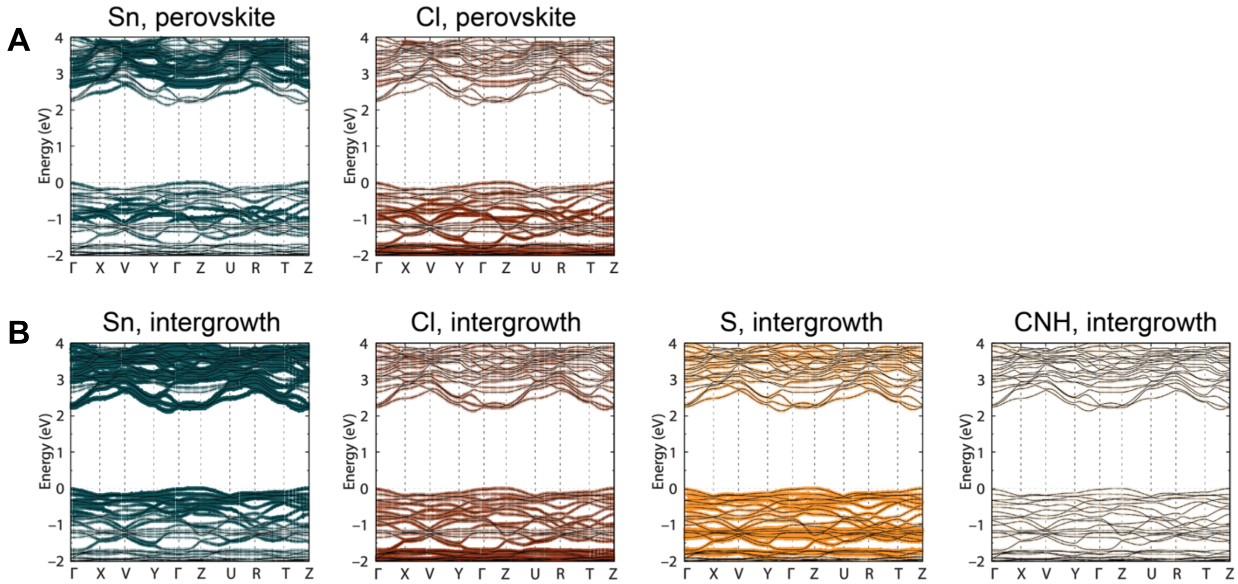

Figure S13. Elemental contributions from (A) the perovskite layer and (B) the intergrowth layer to the electronic band structure of **Sn_CYS**. The dot size is proportional to the elemental contribution.

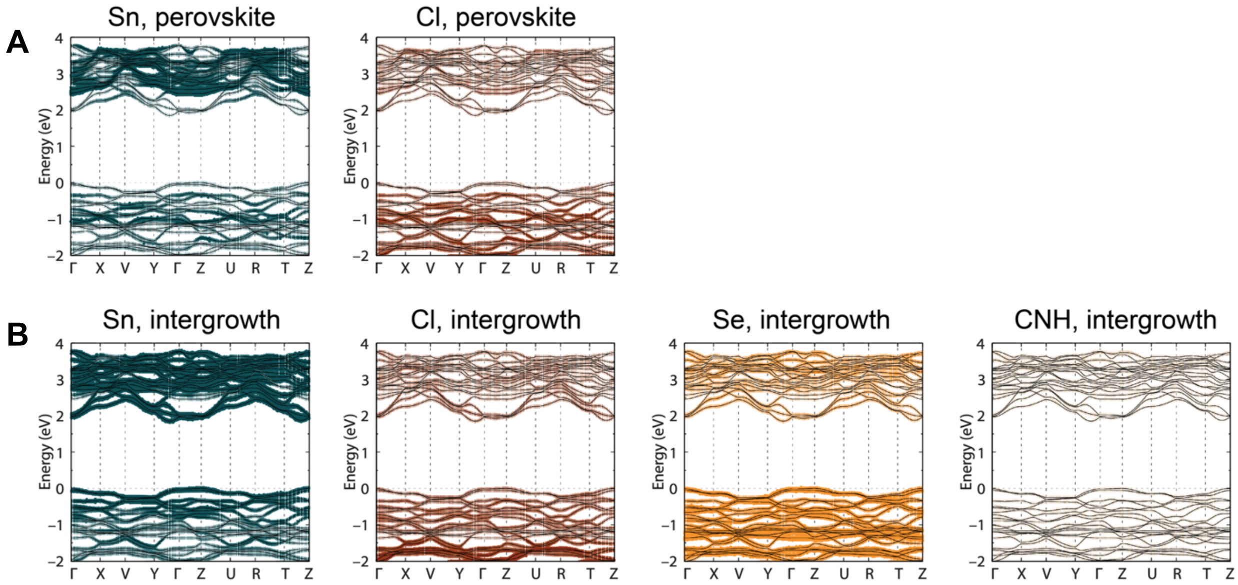

Figure S14. Elemental contributions from (A) the perovskite layer and (B) the intergrowth layer to the electronic band structure of **Sn_SeCYS**. The dot size is proportional to the elemental contribution.

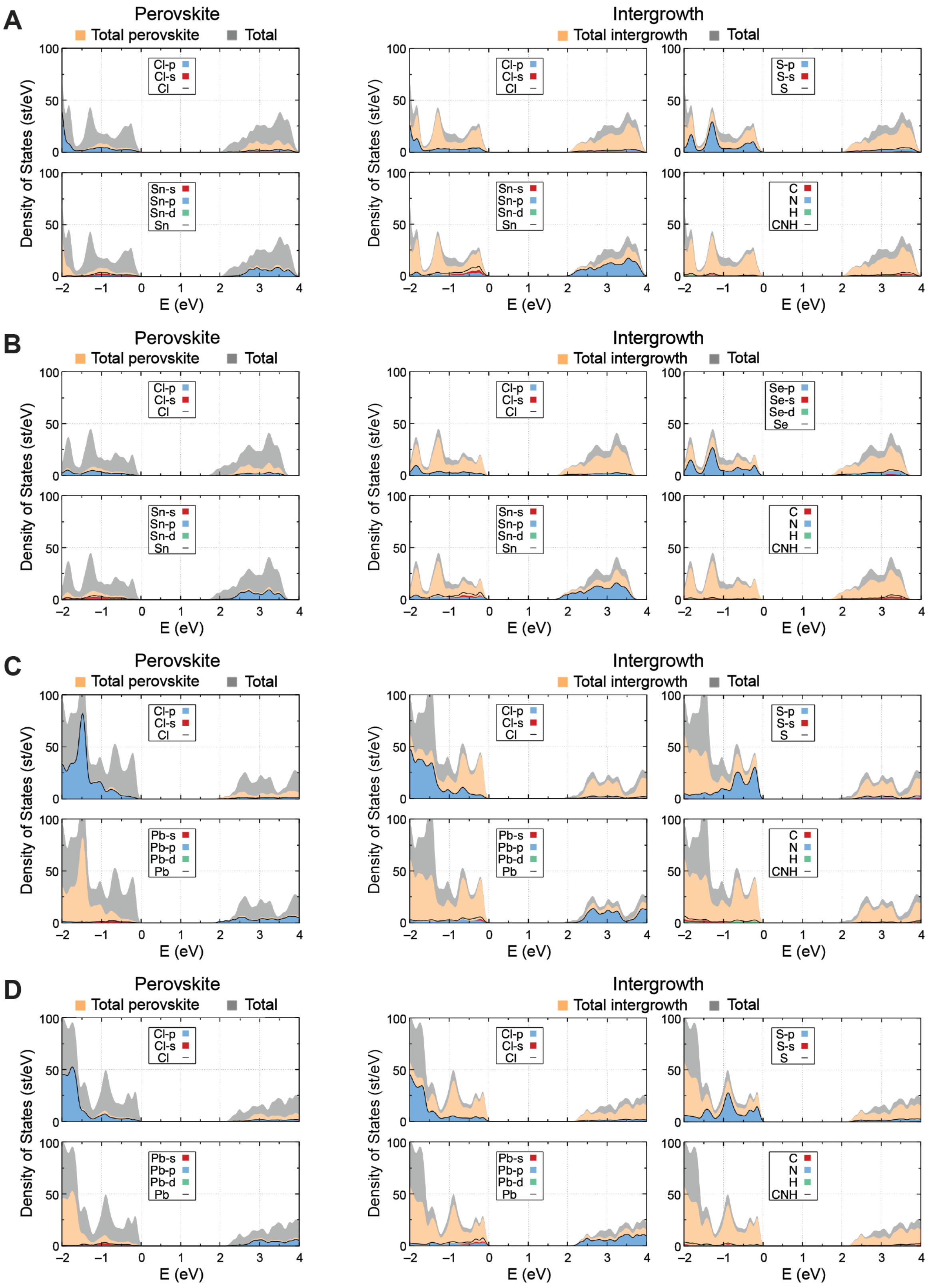

Figure S15. Projected density of states from the perovskite layer and intergrowth layer of (A) **Sn_CYS**, (B) **Sn_SeCYS**, (C) **Pb_CYS**, and (D) **Pb@Sn_CYS**.

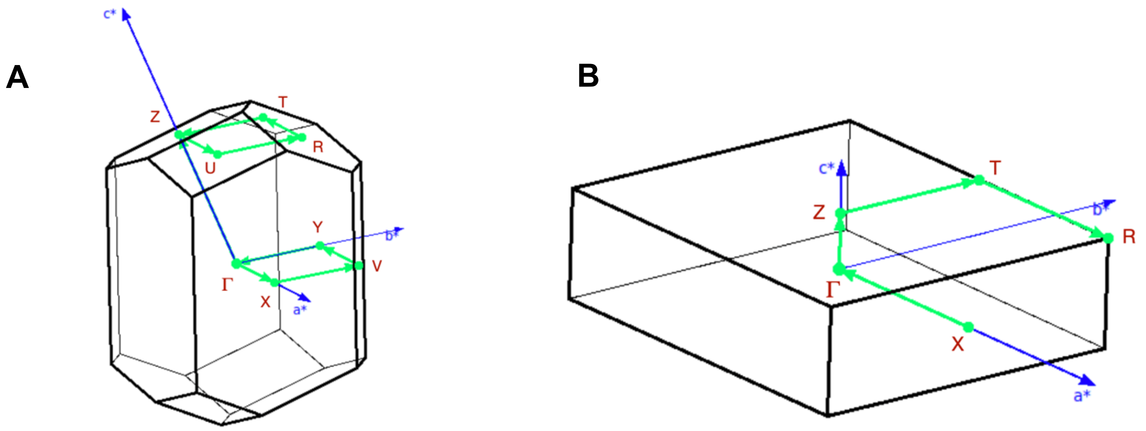

Figure S16. (A) Schematic of the Brillouin Zone and high symmetry path Γ(0,0,0) – X(0.5,0,0) – V(0.5,0.5,0) – Y(0,0.5,0) – Γ(0,0,0) – Z(0,0,0.5) – U(0.5,0,0.5) – R(0.5,0.5,0.5) – T(0,0.5,0.5) – Z(0,0,0.5) for triclinic symmetry. (B) Schematic of the Brillouin Zone and high symmetry path X(0.5,0,0) – Γ(0,0,0) – Z(0,0.5,0) – T(0,0.5,0.5) – R(0.5,0.5,0.5) for orthorhombic symmetry.[41,42]

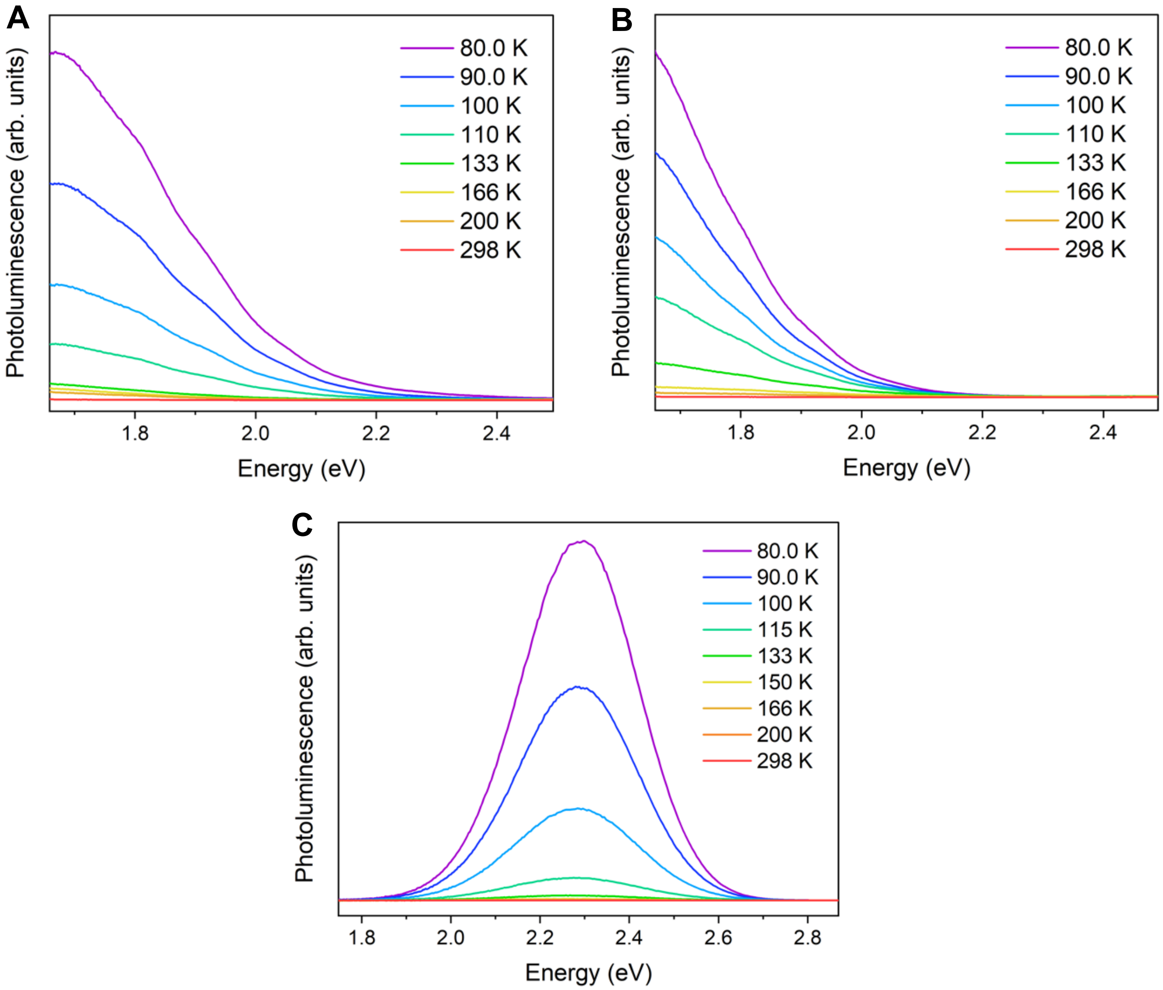

Figure S17. Temperature-dependent photoluminescence of (A) **Sn_CYS**, with 377 nm excitation; (B) **Sn_SeCYS**, with 377 nm excitation; and (C) **Sn_control**, with 340 nm excitation.

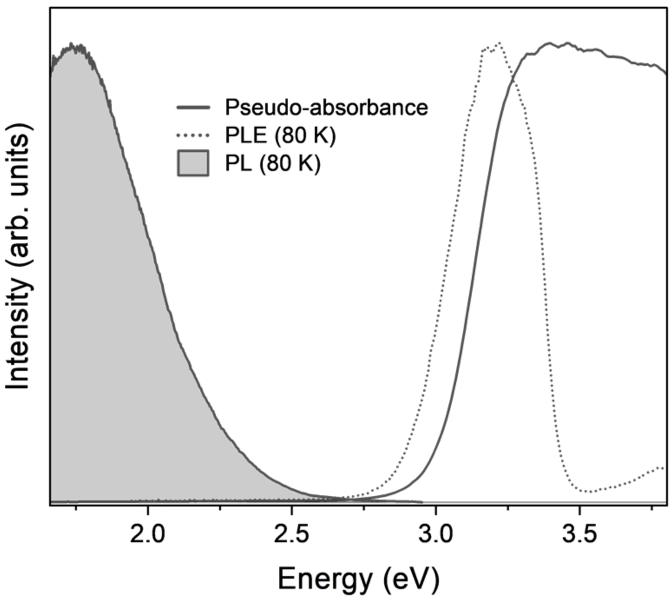

Figure S18. Diffuse reflectance pseudo-absorbance (room temperature), photoluminescence spectra (80 K, excitation at 377 nm), and photoluminescence excitation spectra (80 K, monitoring emission at 670 nm) of powders of **Pb_CYS**.

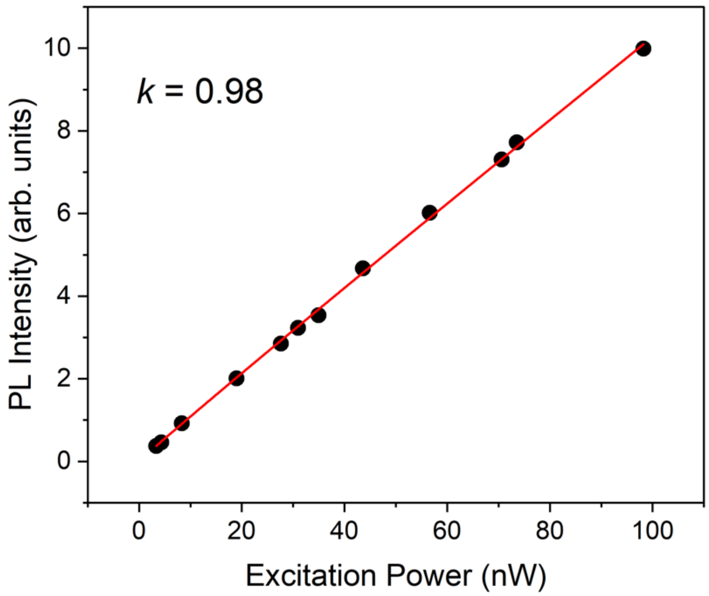

Figure S19. Linear relationship between excitation power and emission intensity for a crystal of **Sn_CYS** . The emission intensities were integrated between 554–905 nm. The red curve shows a fit to $I = aP^k$, where $k = 0.98(1)$.

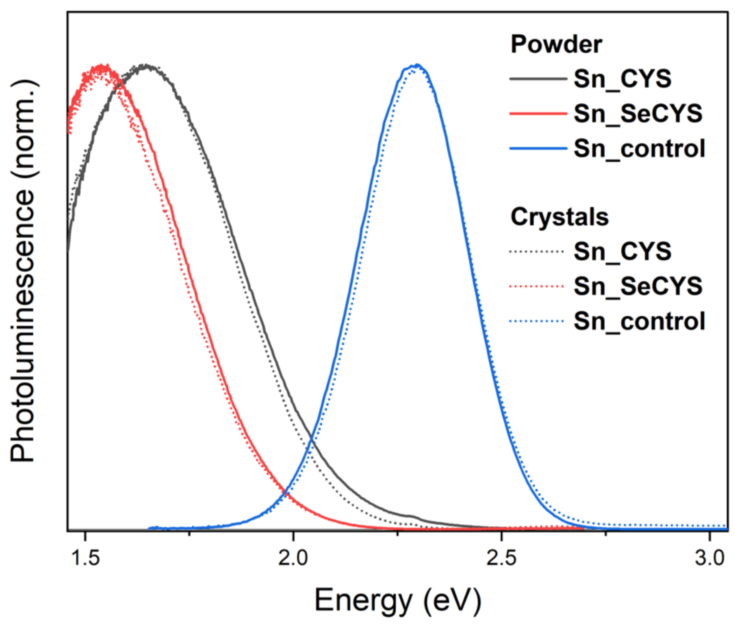

Figure S20. Photoluminescence spectra at 80 K of collections of as-synthesized crystals (dashed lines) and pulverized powders (solid lines) of **Sn_CYS**, **Sn_SeCYS** (377 nm excitation), and **Sn_control** (340 nm excitation).

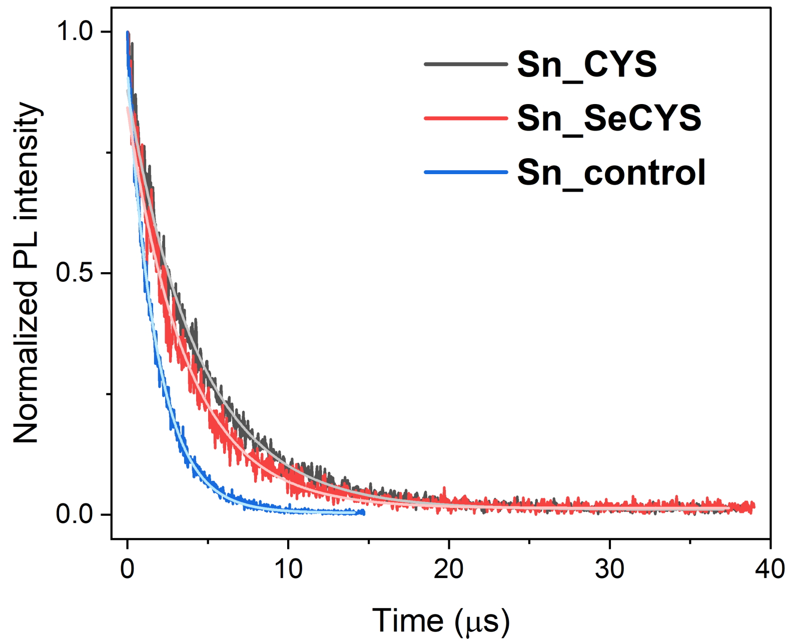

Figure S21. Time-resolved photoluminescence data and single-exponential fits for collections of crystals of **Sn_RCh** (372 nm excitation) and **Sn_control** (331 nm excitation). For **Sn_RCh**, the *x*-axis was shifted to omit scattering artifacts from the samples in the first 3–4 μs. For **Sn_control**, the peak of the decay curve was shifted to 0 μs to normalize the *x*-axis. Emission wavelengths: 730 nm (**Sn_CYS**), 740 nm (**Sn_SeCYS**), 540 nm (**Sn_control**).

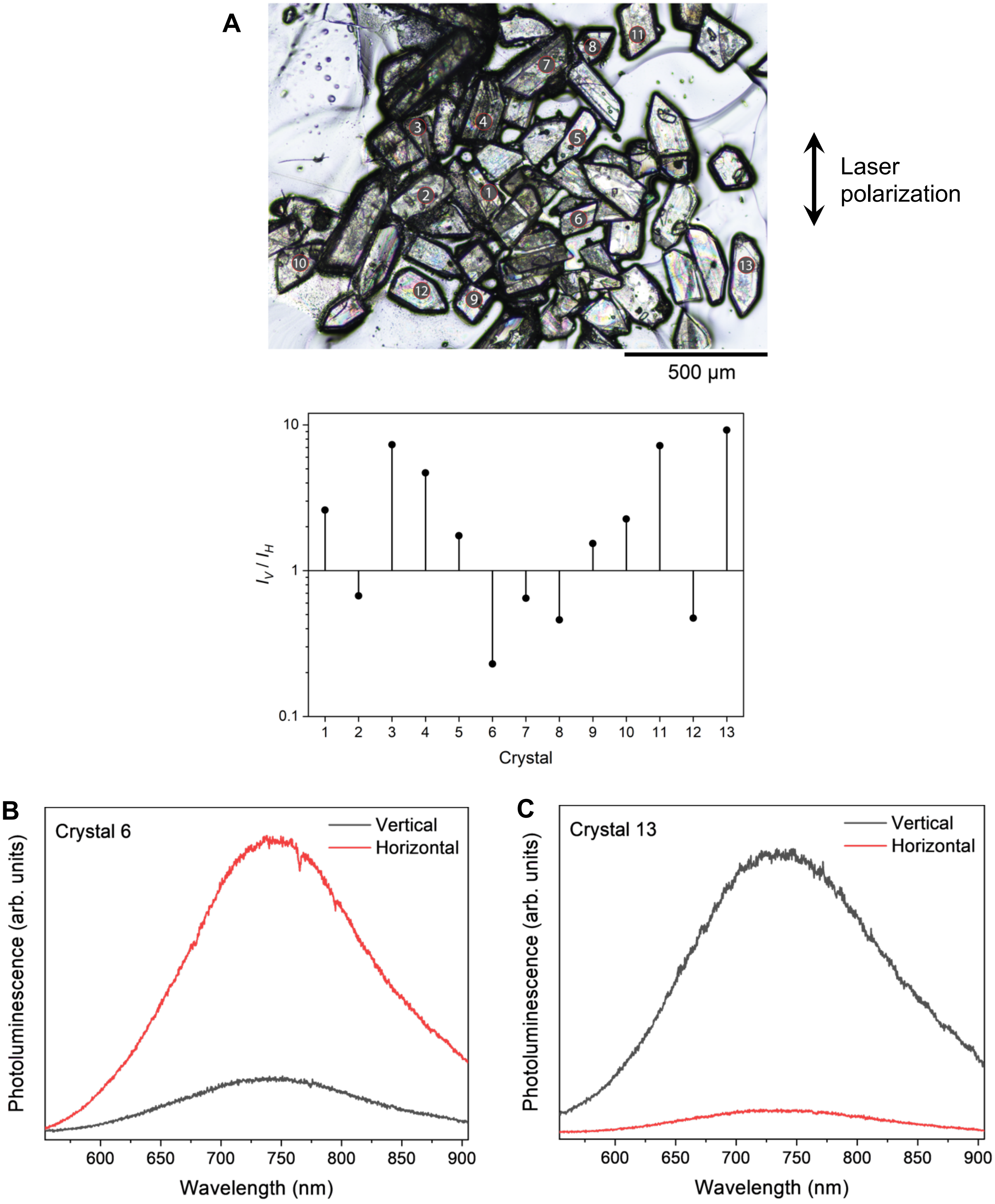

Figure S22. (A) Relative intensity of horizontally and vertically polarized emission for a collection of crystals with various in-plane orientations. (B,C) Vertically and horizontally polarized emission for (B) crystal 6; (C) crystal 13.

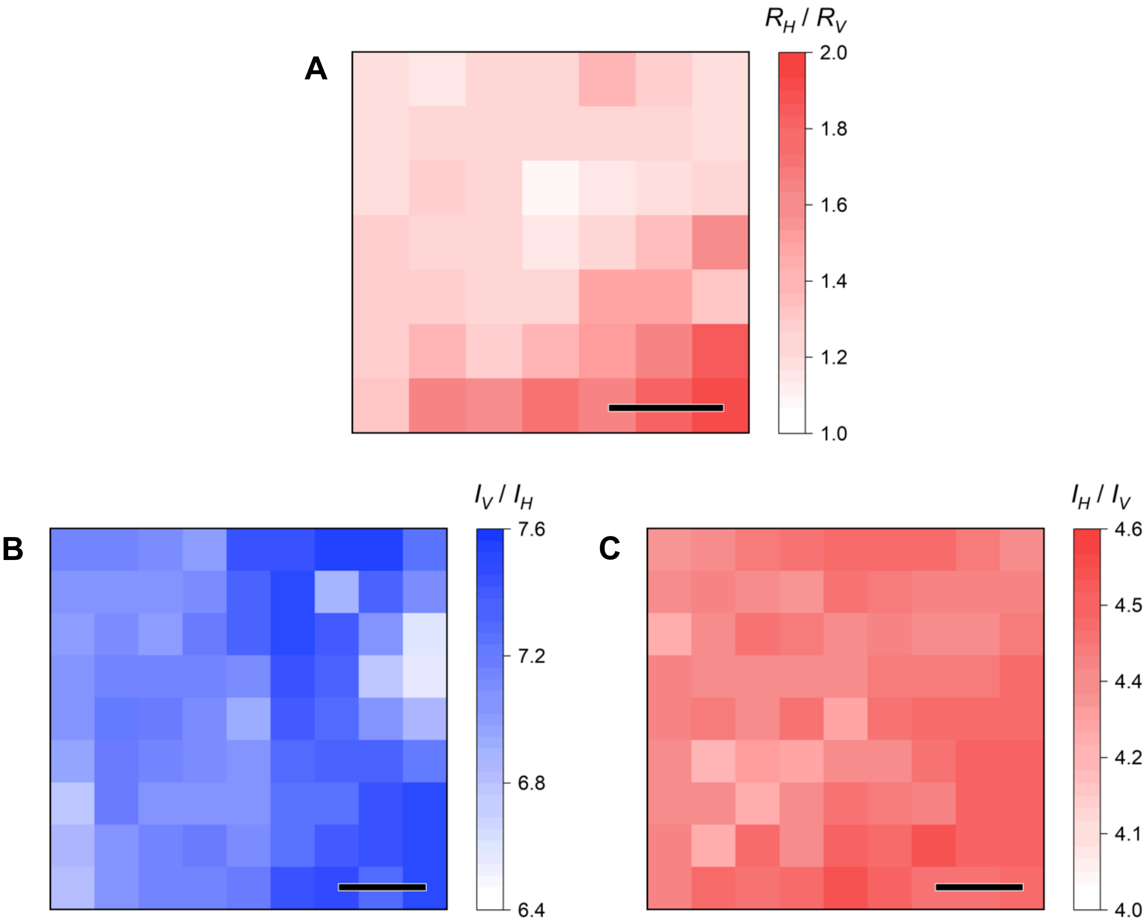

Figure S23. (A) Spatial map of R_H/R_V for a crystal oriented with the *a*-axis at an angle of ca. 62º (0º is horizontal), where R_H and R_V are the integrated intensities for the horizontally polarized and vertically polarized components of the reflectance of a 730 nm vertically polarized laser. (B) Spatial map of I_V/I_H, where I_V and I_H are the integrated intensities of the vertically polarized and horizontally polarized components of the emission from a crystal oriented with the *a*-axis at an angle of ca. 78º, showing small variation of I_V/I_H between 6.6–7.6. (C) Spatial map of I_H/I_V for a crystal with the *a*-axis oriented at an angle of ca. 19º, showing small variation of I_H/I_V between 4.3–4.5.The homogeneity of I_V/I_H across each crystal suggests that the measured emission anisotropy is a property of the bulk crystal. Slight variations may be due to differences in defects, surface quality, and domains across a large area of the crystal. Scale bars represent 10 µm.

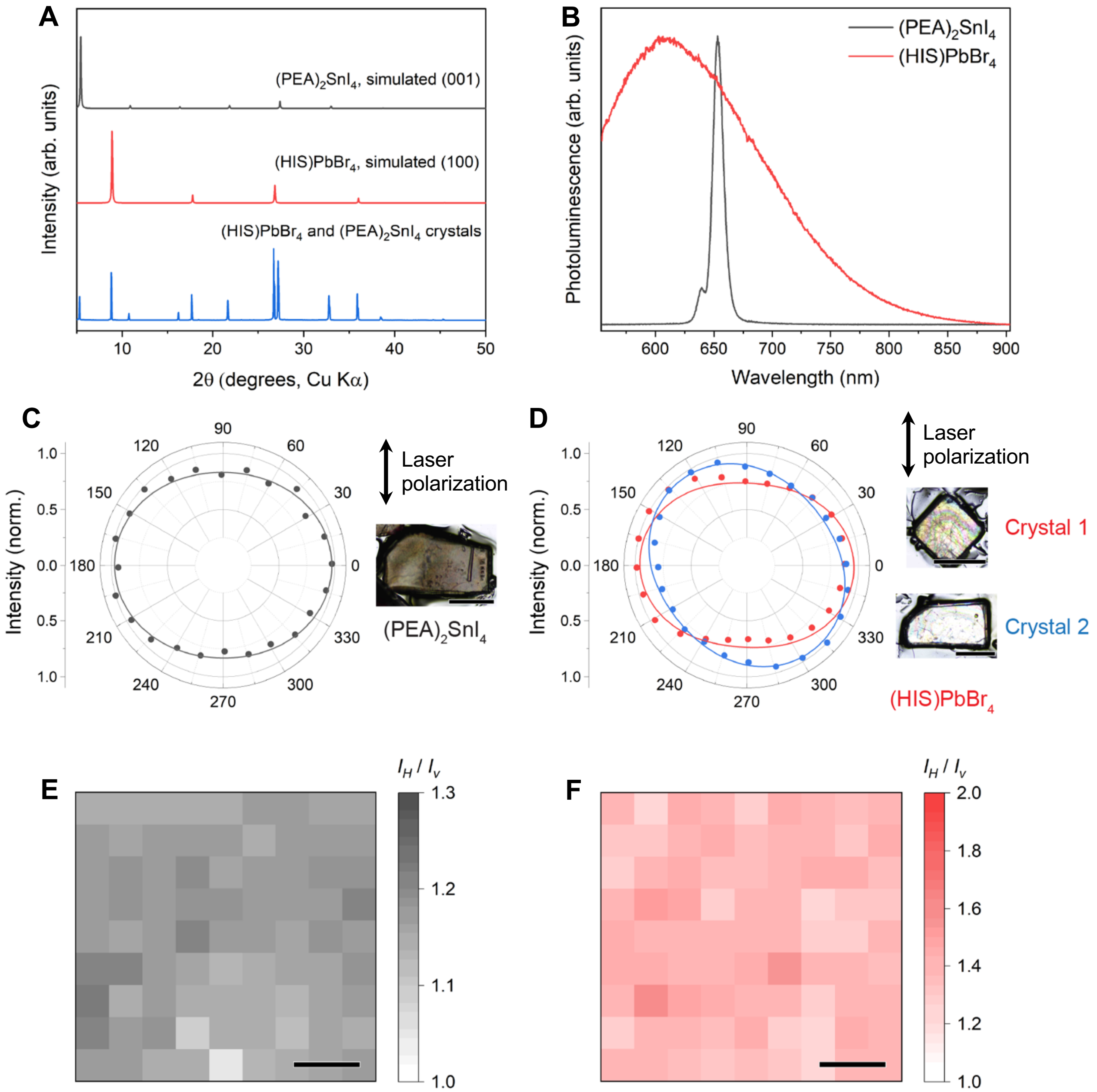

Figure S24. (A) PXRD pattern of crystals of $(PEA)_2SnI_4$ and $(HIS)PbBr_4$, mounted on a silicon substrate for linearly polarized photoluminescence (PL) measurements, confirming the orientation of the crystals with the inorganic planes parallel to the substrate.[5,6,43] (B) PL spectra of $(PEA)_2SnI_4$ and $(HIS)PbBr_4$ at 80 K, with 377 nm vertically polarized excitation. (C, D) Polar plots of PL intensity as a function of the angle of a linear polarizer in the emission path for single crystals of (C) $(PEA)_2SnI_4$ and (D) $(HIS)PbBr_4$. Scale bars for crystal images represent 500 µm. (E, F) Spatial maps of I_H/I_V, where I_H and I_V are the total integrated intensities of the horizontally polarized and vertically polarized components, respectively, of the emission from crystals of (E) $(PEA)_2SnI_4$ and (F) $(HIS)PbBr_4$, with the major emission axis oriented horizontally. For these horizontally oriented crystals, the ratio I_H/I_V is maximized and represents the anisotropy ratio. Scale bars for spatial maps represent 10 µm.

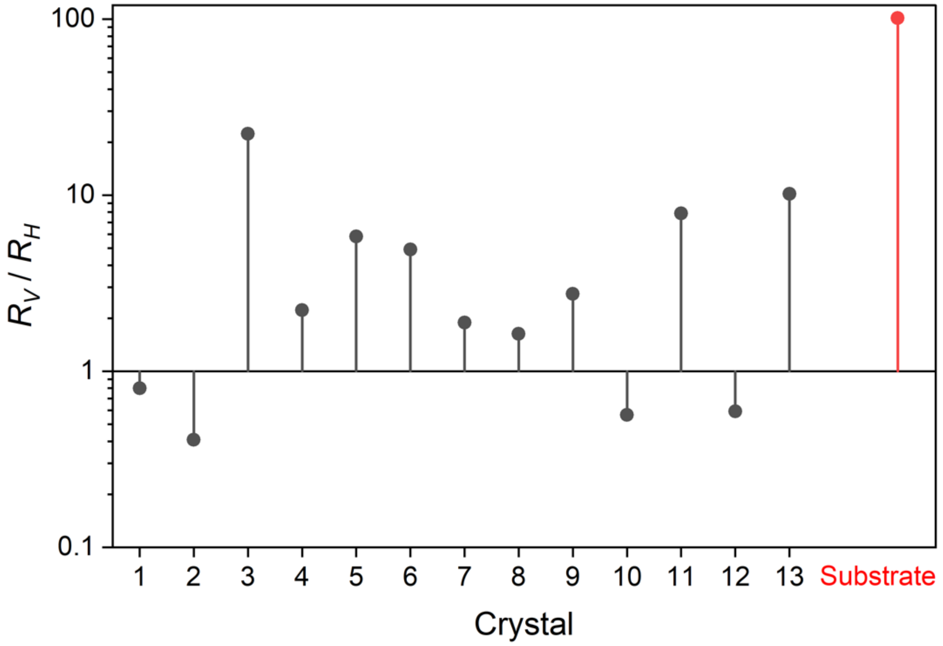

Figure S25. Values of R_V/R_H, where R_V and R_H are the integrated intensities of the vertically polarized and horizontally polarized components, respectively, of the reflectance of a 730 nm vertically polarized laser from crystals of **Sn_CYS**. The orientations of the crystals are shown in Figure S22. Reflectance off the PMMA-coated substrate alone maintains the vertical polarization of the incident laser. Notably, crystals with the long axis aligned closest to the direction of the laser polarization (crystals 3, 11, and 13), show the smallest change in polarization upon reflectance, suggesting that either the fast or the slow axis is aligned with the crystallographic *a*-axis.

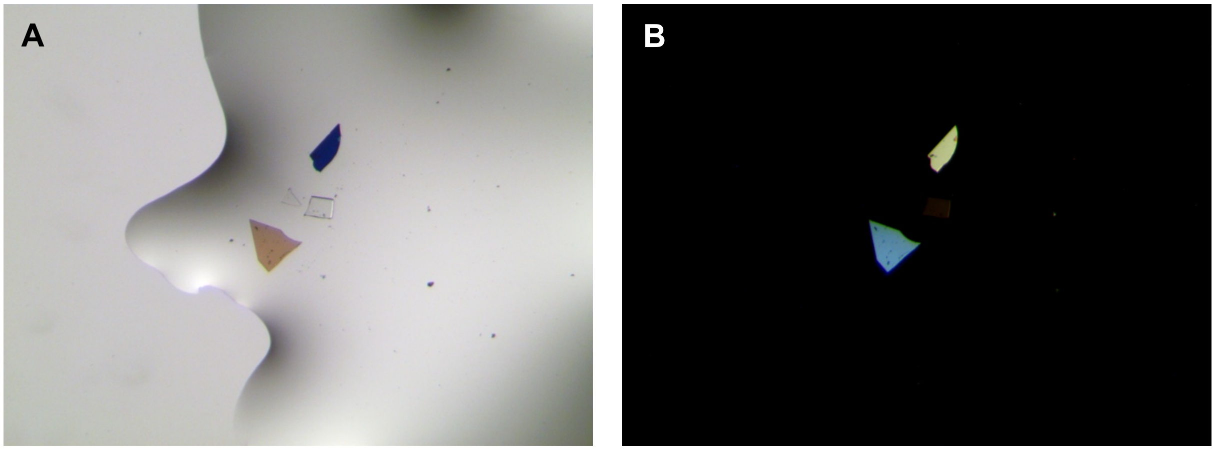

Figure S26. Single crystals of **Sn_CYS** in microscope immersion oil in (A) plane-polarized illumination and (B) cross-polarized illumination. The blue and yellow interference colors observed in cross-polarized light indicate birefringence.

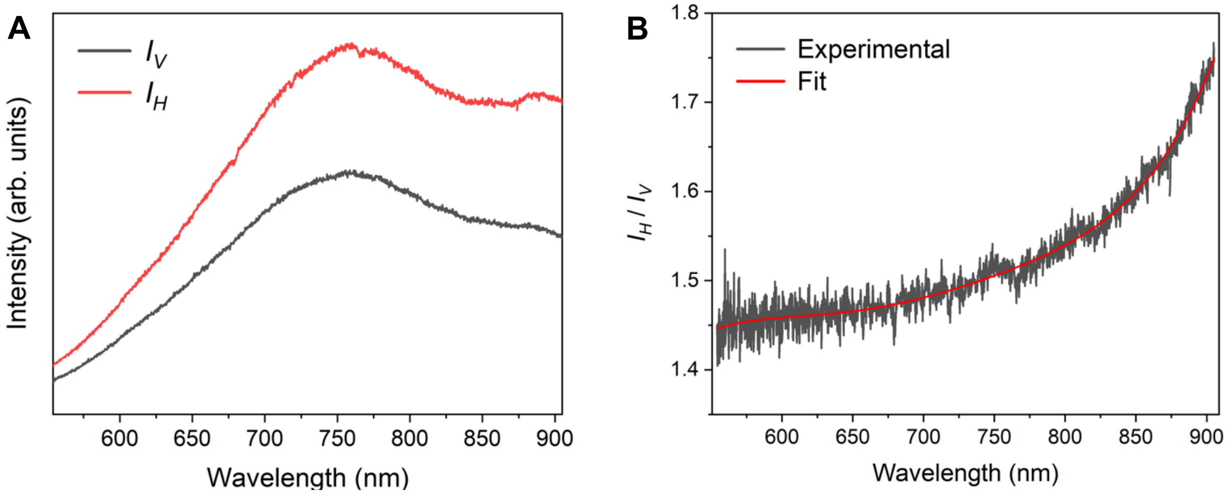

Figure S27. Calibration of the linearly-polarized photoluminescence setup to account for intrinsic polarization bias in the optical components. We used an incandescent lightbulb as an unpolarized light source. The light emitted from the lightbulb propagated through the entire emission path as used in photoluminescence measurements, and the horizontally and vertically polarized components were measured simultaneously. This control revealed a slight bias for horizontally polarized light. (A) Recorded vertical intensity (I_V) and horizontal intensity (I_H) for the incandescent lightbulb, showing a bias for horizontal polarization. (B) Ratio of I_H/I_V as a function of wavelength. The I_H/I_V ratios from two sets of spectra were averaged then fit to a polynomial (red curve) to reduce the effects of noise. The resulting calibration curve was used to scale all I_H and R_H spectra from linearly polarized photoluminescence and reflectance experiments.

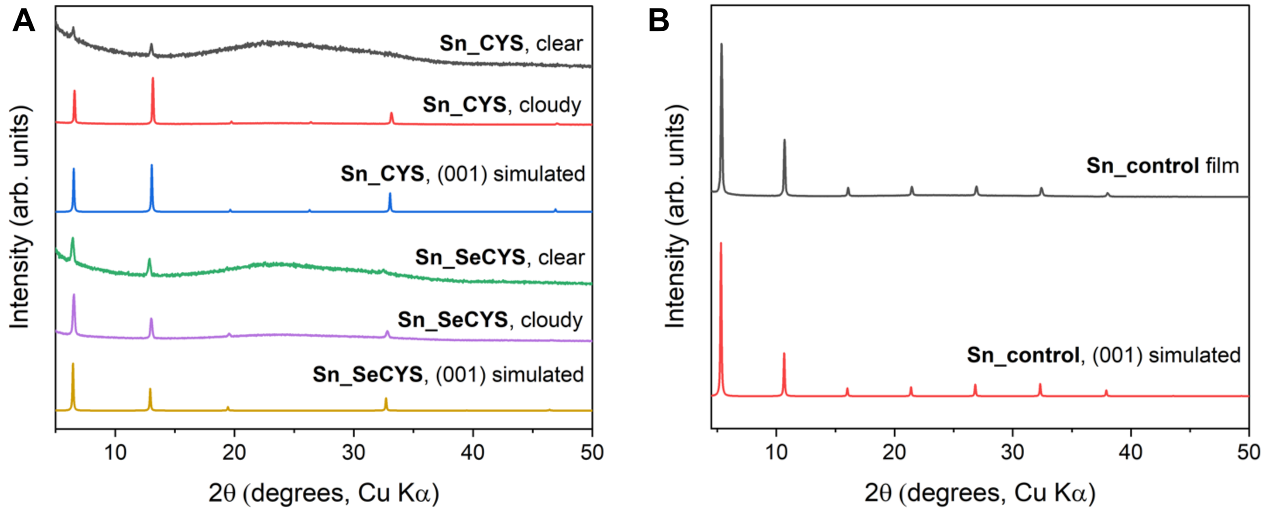

Figure S28. (A) Experimental powder X-ray diffraction patterns of films of **Sn_CYS** and **Sn_SeCYS**, which agree with the simulated patterns from the single-crystal X-ray diffraction structures with (001) preferential orientation (in the $P\bar{1}$ space group). (B) Experimental powder X-ray diffraction patterns of films of **Sn_control**, which agree with the simulated patterns from the single-crystal X-ray diffraction structures with (001) preferential orientation.

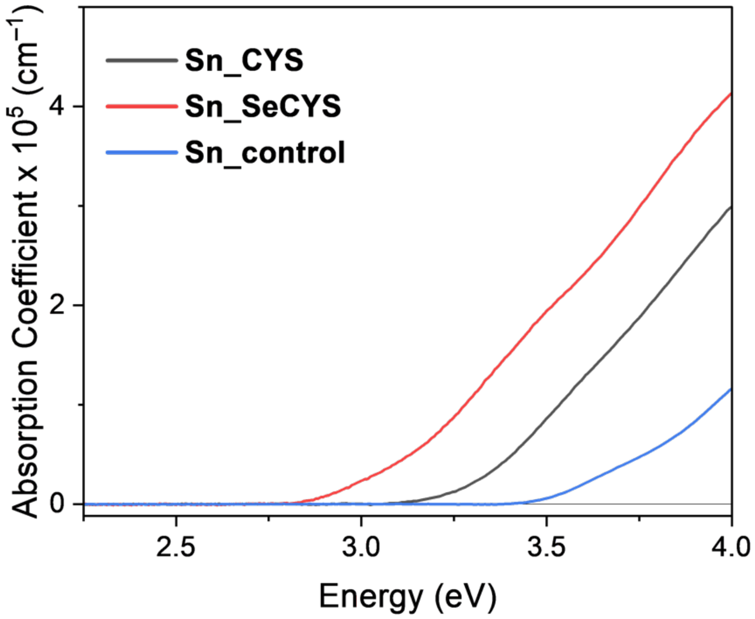

Figure S29. Absorption coefficients of **Sn_CYS**, **Sn_SeCYS**, and **Sn_control**, from transmission measurements of thin films.

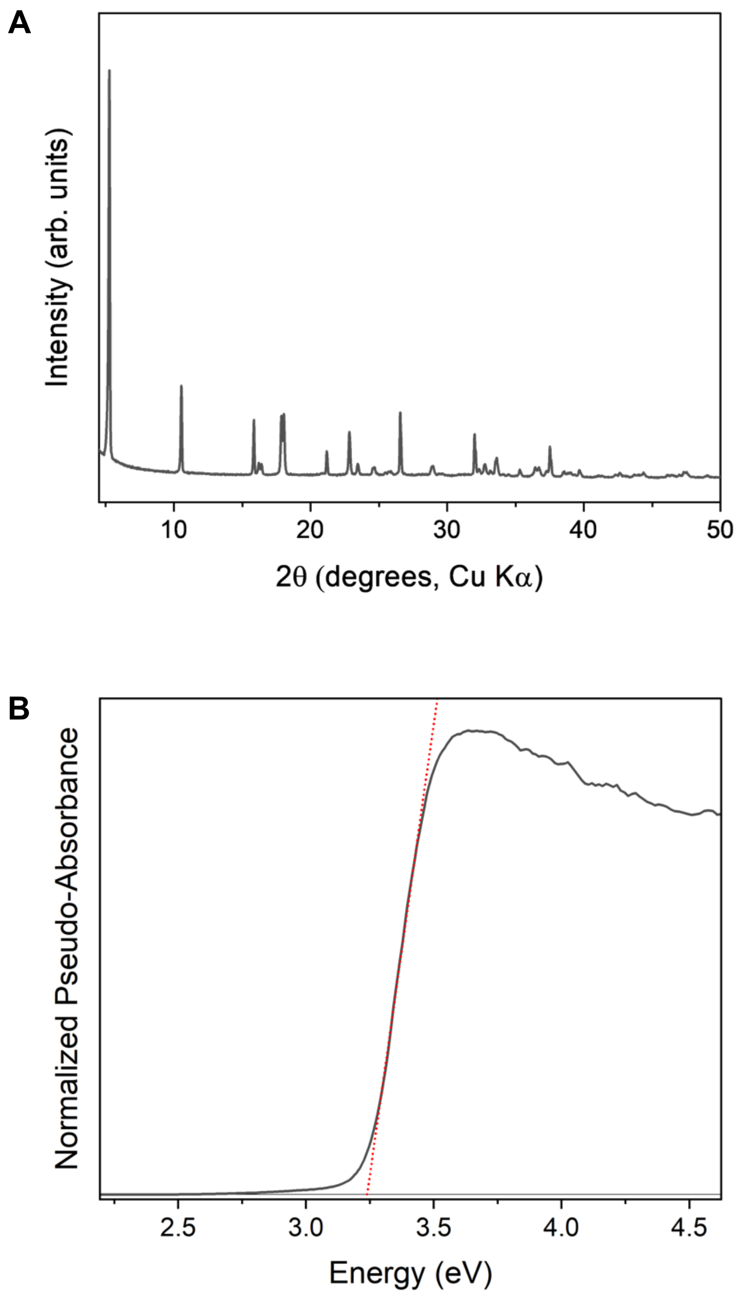

Figure S30. (A) Experimental PXRD pattern of $(BZA)_2SnCl_4$, synthesized based on a previously reported procedure.[4] (B) Diffuse reflectance spectrum of $(BZA)_2SnCl_4$. Fitting to the linear portion of the plot gives an absorption onset of ca. 3.2 eV. We were unable to reproduce the reported bandgap of 3.04 eV.

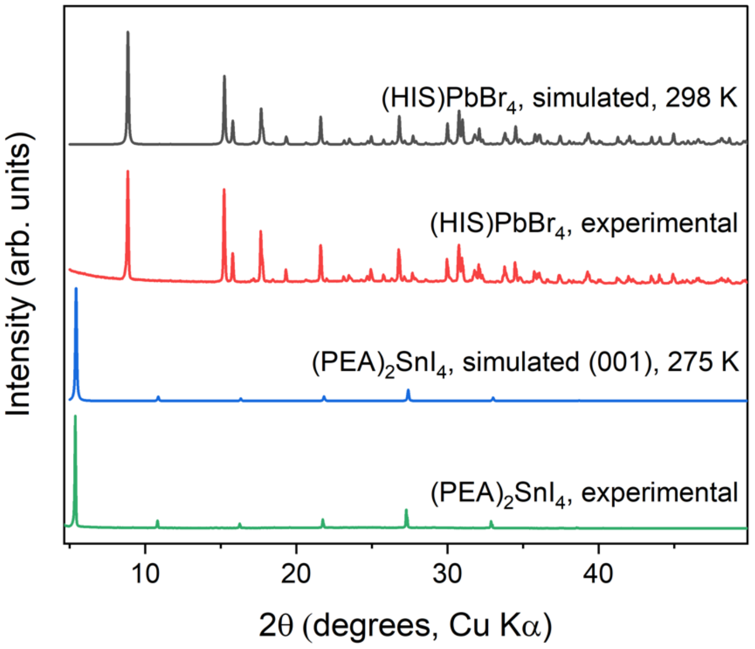

Figure S31. (A) Experimental room-temperature PXRD patterns of pulverized crystals of (HIS)$PbBr_4$ and $(PEA)_2SnI_4$, compared with simulated patterns.[5,43] The PXRD pattern of $(PEA)_2SnI_4$ shows strong (001) orientation even after grinding.

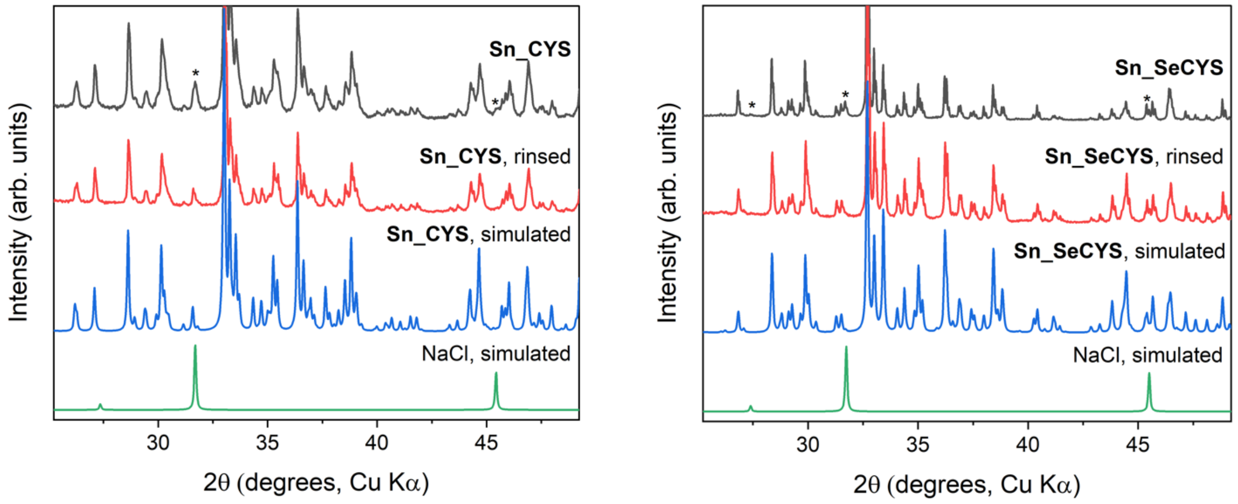

Figure S32. Evidence of small amounts of NaCl in the PXRD patterns of **Sn_RCh**, residual from the concentrated NaCl aqueous solvent used in the synthesis.[44] Residual NaCl can be removed by rinsing the crystals with dilute aqueous NaCl during filtration.

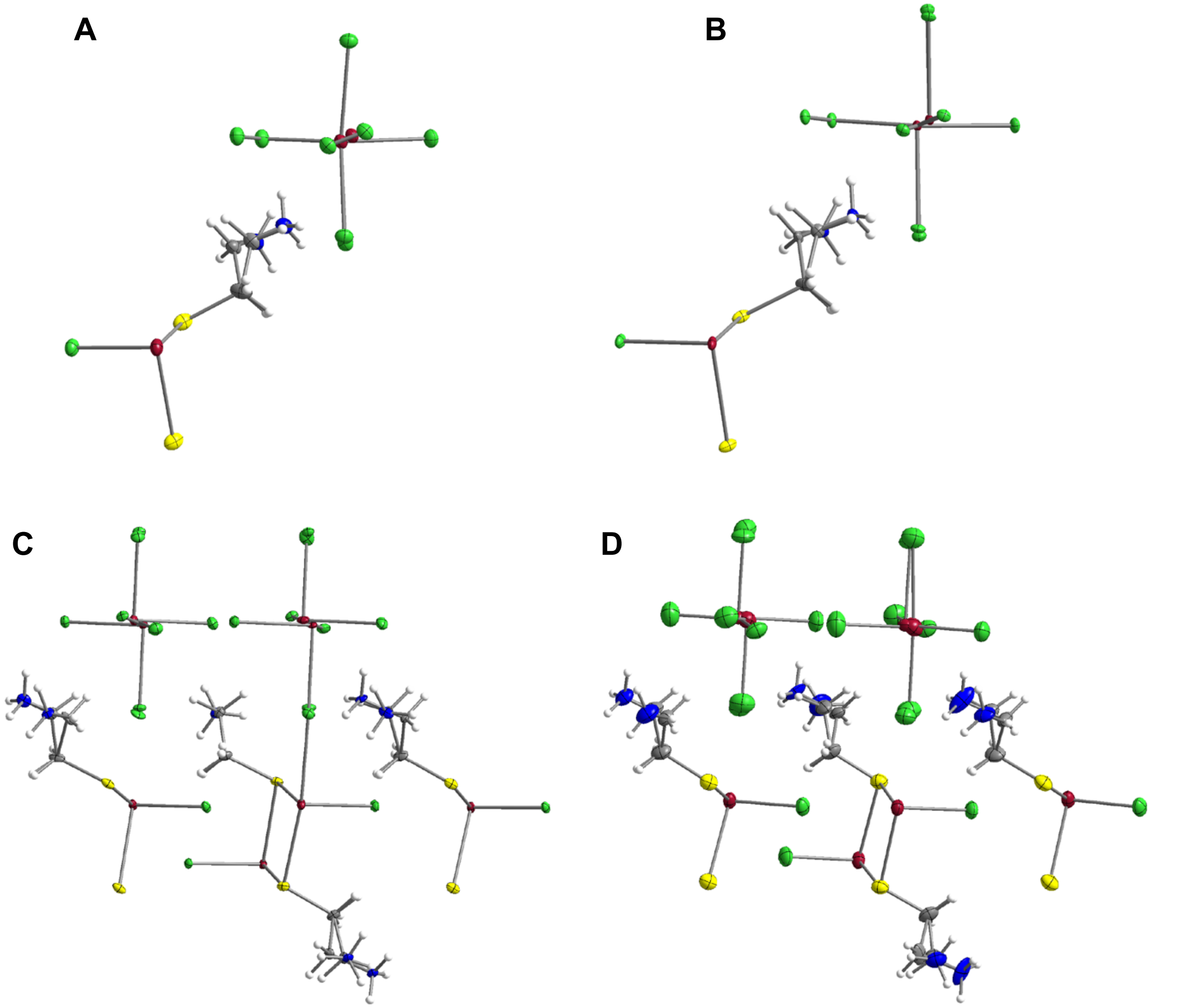

Figure S33. Structures and thermal ellipsoids of **Sn_CYS**, for (A) 100 K, *C*2/*c*; (B) 80 K, *C*2/*c*; (C) 100 K, $P\bar{1}$; (D) 300 K, $P\bar{1}$ crystal structures. Thermal ellipsoids with 50% probability are shown for Sn (maroon), Cl (green), S (yellow), N (blue), and C (grey) atoms. Hydrogen atoms (white) are depicted as spheres.

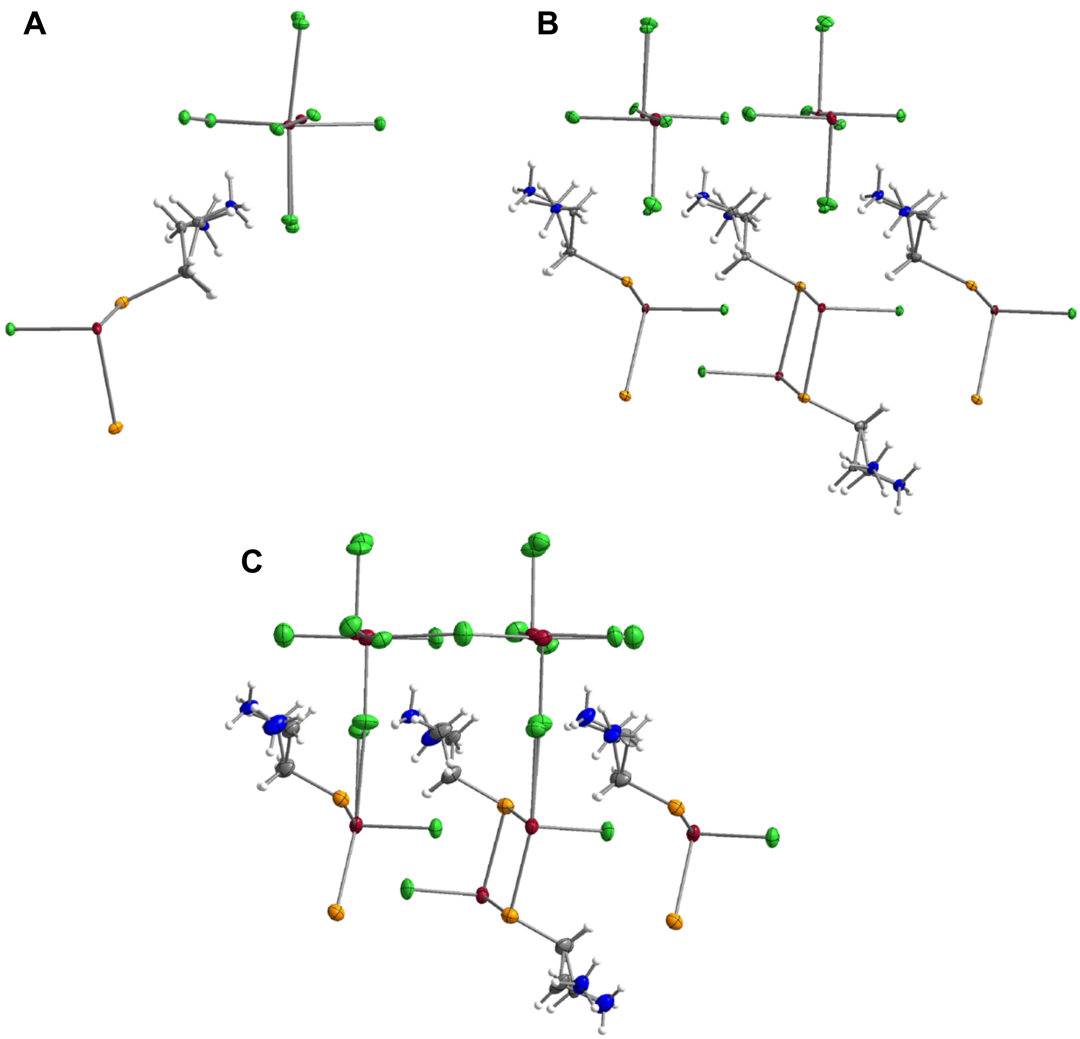

Figure S34. Structures and thermal ellipsoids of **Sn_SeCYS,** for (A) 100 K, *C*2/*c*; (B) 100 K, $P\bar{1}$; (C) 300 K, $P\bar{1}$ crystal structures. Thermal ellipsoids with 50% probability are shown for Sn (maroon), Se (orange), Cl (green), N (blue), and C (grey) atoms. Hydrogen atoms (white) are depicted as spheres.

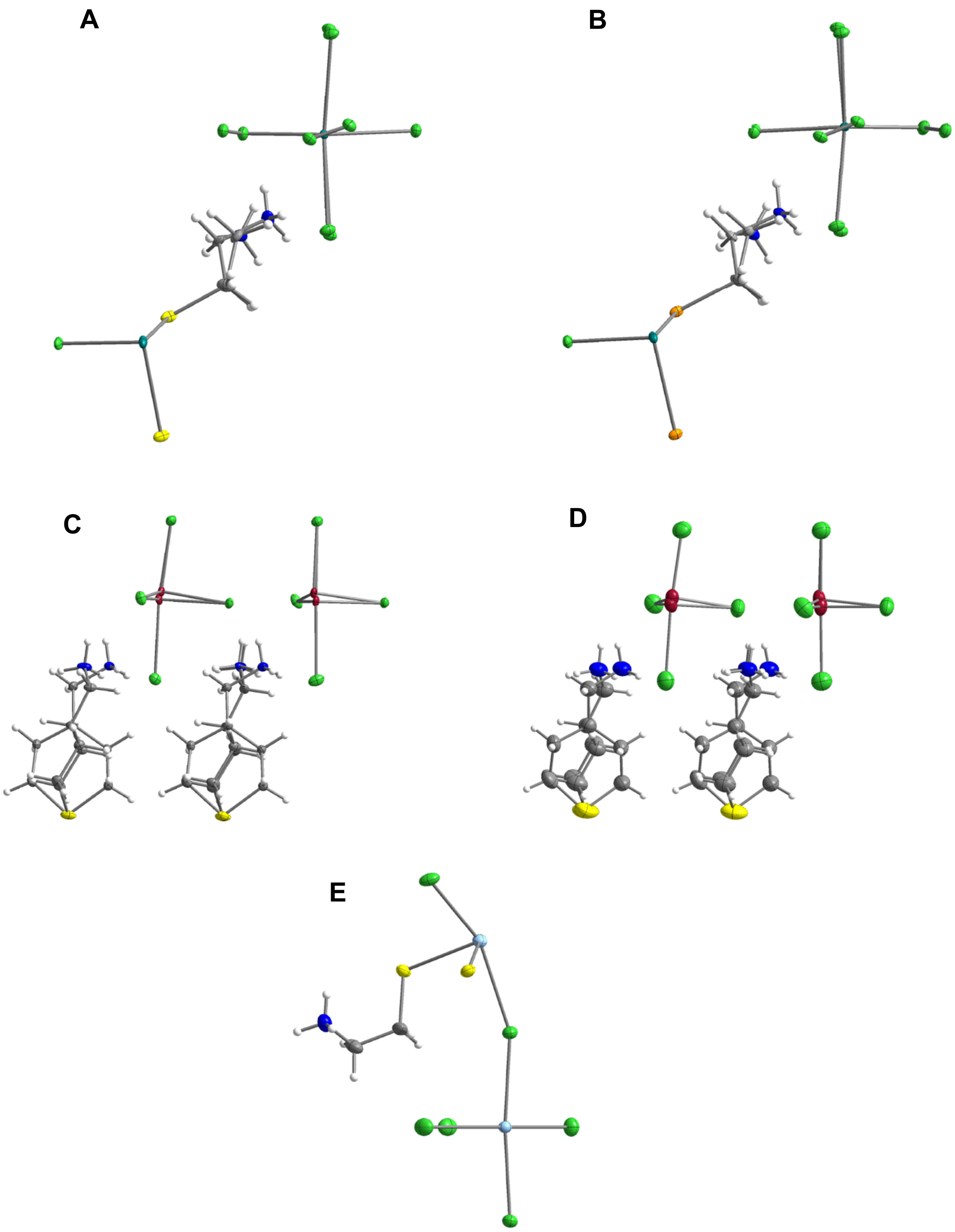

Figure S35. Structures and thermal ellipsoids of (A) **Sn_CYS:Pb**, 100 K; (B) **Sn_SeCYS:Pb**, 100 K; (C) **Sn_control**, 100 K; (D) **Sn_control**, 300 K; (E) **Pb_CYS**, 300 K. Thermal ellipsoids with 50% probability are shown for Pb (turquoise), Sn (maroon), Sn/Pb (teal), Se (orange), Cl (green), S (yellow), N (blue), and C (grey) atoms. Hydrogen atoms (white) are depicted as spheres.

5. Supplementary Discussion

5.1 PL intensity dependence on excitation power density

In order for emission from permanent defects to show a linear dependence on power density, excitation rate << relaxation rate.[45] Here, we follow the analysis of Karunadasa et al.[46] to estimate the defect density that would be required to observe a linear power dependence in **Sn_CYS** from permanent material defects.

σ = absorption cross section, A = absorbance, ε_M = molar extinction coefficient, L = film thickness, C = concentration, N_A = Avogadro's number, q_p = laser photon flux, I = laser intensity, h = Planck's constant, c = velocity of light, λ = laser wavelength.

<u>Excitation rate = σq_p</u>
$A = \varepsilon_M \times C \times L$
$A/L = 1 \times 10^4$ cm^{-1} at 377 nm and $C = 0.00374$ mol/cm^3 (obtained from formula units per unit cell volume at 80 K)
$\varepsilon_M = 2.68 \times 10^6$ cm^2/mol
$\sigma = \ln(10) \times (\varepsilon_M / N_A) = 1.02 \times 10^{-17}$ cm^2 / formula unit
$I = 3$ W/cm^2
$q_p = I\lambda/hc = 5.69 \times 10^{18}$ s^{-1}cm^{-2}
$\sigma q_p = (1.02 \times 10^{-17}$ cm^2 / formula unit$) \times (5.69 \times 10^{18}$ s^{-1}cm$^{-2}) = 58$ (s^{-1} / formula unit)

<u>Relaxation rate = σq_p</u>
N = number of emissive defects per formula unit. PL lifetime at 77 K = 4×10^{-6} s

If emission arises from permanent material defects, because we see no sign of PL saturation at a laser power density of 3 W/cm^3:
Excitation rate << relaxation rate
58 (s^{-1} / formula unit) << $N/(4 \times 10^{-6}$ s)
So, N >> 10^{-4} defects per formula unit, or N' >> 10^{17} defects/cm^3

A defect density significantly larger than 10^{17} defects/cm^3 is unlikely to occur intrinsically in the crystal used in this measurements.[47–49] The linear dependence of emission intensity on incident power density suggests that the emission originates from light-generated defects, rather than permanent material defects.

5.2 Relationship between crystal angle and polarization angle for the linearly polarized photoluminescence of Sn_CYS

We can express the polarization of the emission from a crystal with two orthogonal linearly polarized components: a, corresponding to the intensity polarized along the major emission axis, and b, corresponding to the intensity polarized along the minor emission axis. Let the angle θ represent the angle of the major emission axis with respect to horizontal (the "polarization angle").

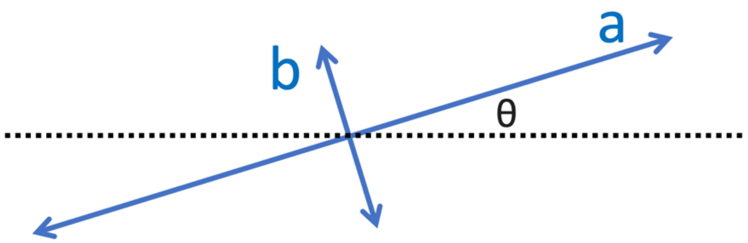

The intensity transmitted through a vertical polarizer (I_V) and a horizontal polarizer (I_H) will be:

$$I_V = asin^2\theta + bcos^2\theta \quad (1)$$
$$I_H = acos^2\theta + bsin^2\theta \quad (2)$$

Therefore:

$$\frac{I_V}{I_H} = \frac{asin^2\theta + bcos^2\theta}{acos^2\theta + bsin^2\theta} \quad (3)$$

Let c be the polarization anisotropy ratio: $c = {}^{a}/_{b}$. Then:

$$\frac{I_V}{I_H} = \frac{csin^2\theta + cos^2\theta}{ccos^2\theta + sin^2\theta} \quad (4)$$

We measured $\frac{I_V}{I_H}$ for 13 crystals, using a Wollaston prism to simultaneously record I_V and I_H. We also estimated the angle of each crystal, defined as the angle of the a-axis with respect to horizontal, from optical microscopy images (see Figure S22).

Fitting to Equation 4 gives c = 9.5(3), with an R^2 value of 0.990.

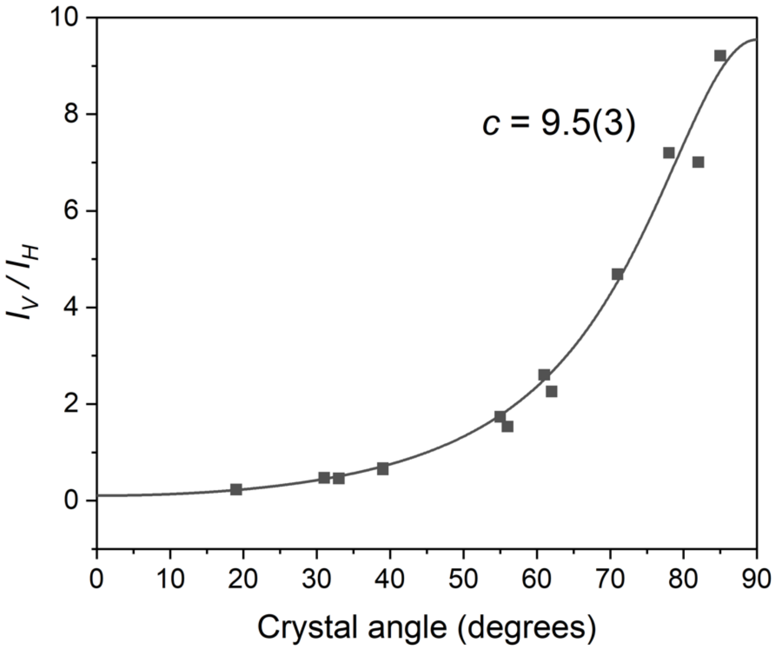

Then, using Equation 4 and the calculated anisotropy ratio of c = 9.5, we can convert each experimental ratio of $\frac{I_V}{I_H}$ to the corresponding polarization angle θ. We can thus transform the y-axis of the above plot to the polarization angle θ. The resulting plot of experimental polarization angle (below; Figure 5B) against measured crystal angle shows a linear relationship, with a slope of 1.01(2), intercept of –1(1)º, and R^2 value of 0.994.

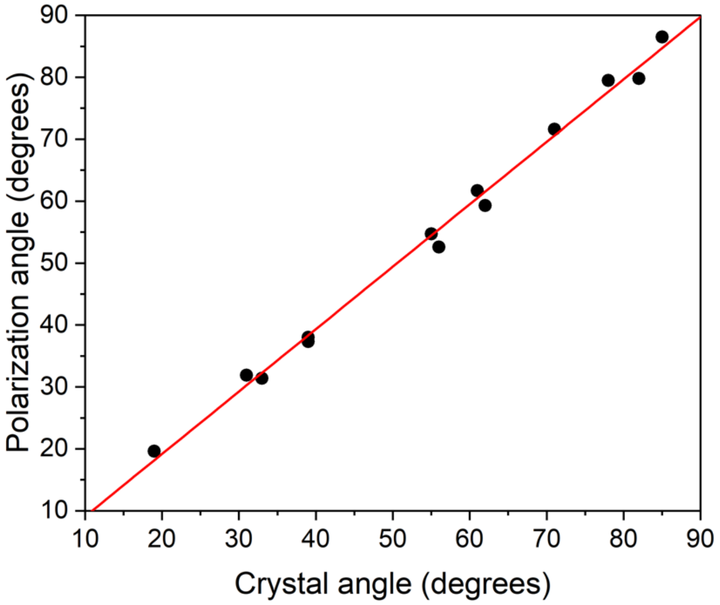

6. References

(1) Li, J.; Wang, Y.; Saha, S.; Chen, Z.; Hofmann, J.; Misleh, J.; Chapman, K. W.; Reimer, J. A.; Filip, M. R.; Karunadasa, H. I. 3D Lead-Organoselenide-Halide Perovskites and Their Mixed-Chalcogenide and Mixed-Halide Alloys. *Angew. Chem. Int. Ed.* **2024**, *63* (41), e202408443. https://doi.org/10.1002/anie.202408443.

(2) Klayman, D. L. The Synthesis of Aminoethyl-Substituted Selenium Compounds. *J. Org. Chem.* **1965**, *30* (7), 2454–2456. https://doi.org/10.1021/jo01018a504.

(3) Aubrey, M. L.; Saldivar Valdes, A.; Filip, M. R.; Connor, B. A.; Lindquist, K. P.; Neaton, J. B.; Karunadasa, H. I. Directed Assembly of Layered Perovskite Heterostructures as Single Crystals. *Nature* **2021**, *597* (7876), 355–359. https://doi.org/10.1038/s41586-021-03810-x.

(4) Pisanu, A.; Coduri, M.; Morana, M.; Ciftci, Y. O.; Rizzo, A.; Listorti, A.; Gaboardi, M.; Bindi, L.; Queloz, V. I. E.; Milanese, C.; Grancini, G.; Malavasi, L. Exploring the Role of Halide Mixing in Lead-Free BZA_2SnX_4 Two Dimensional Hybrid Perovskites. *J. Mater. Chem. A* **2020**, *8*, 1875–1886. https://doi.org/10.1039/c9ta11923j.

(5) Smith, M. D.; Jaffe, A.; Dohner, E. R.; Lindenberg, A. M.; Karunadasa, H. I. Structural Origins of Broadband Emission from Layered Pb–Br Hybrid Perovskites. *Chem. Sci.* **2017**, *8* (6), 4497–4504. https://doi.org/10.1039/C7SC01590A.

(6) Gao, Y.; Wei, Z.; Yoo, P.; Shi, E.; Zeller, M.; Zhu, C.; Liao, P.; Dou, L. Highly Stable Lead-Free Perovskite Field-Effect Transistors Incorporating Linear π-Conjugated Organic Ligands. *J. Am. Chem. Soc.* **2019**, *141* (39), 15577–15585. https://doi.org/10.1021/jacs.9b06276.

(7) Bruker, APEX. SAINT and SADABS, Bruker AXS Inc. Madison, Wisconsin, USA. **2007**.

(8) Sheldrick, G. M. *SHELXT* – Integrated Space-Group and Crystal-Structure Determination. *Acta Crystallogr. A* **2015**, *71* (1), 3–8. https://doi.org/10.1107/S2053273314026370.

(9) Sheldrick, G. M. Crystal Structure Refinement with *SHELXL*. *Acta Crystallogr. C* **2015**, *71* (1), 3–8. https://doi.org/10.1107/S2053229614024218.

(10) Dolomanov, O. V.; Bourhis, L. J.; Gildea, R. J.; Howard, J. A. K.; Puschmann, H. *OLEX2*: A Complete Structure Solution, Refinement and Analysis Program. *J. Appl. Crystallogr.* **2009**, *42* (2), 339–341. https://doi.org/10.1107/S0021889808042726.

(11) Parsons, S. Introduction to Twinning. *Acta Crystallogr. D* **2003**, *59* (11), 1995–2003. https://doi.org/10.1107/S0907444903017657.

(12) Toby, B. H.; Von Dreele, R. B. *GSAS-II*: The Genesis of a Modern Open-Source All Purpose Crystallography Software Package. *J. Appl. Crystallogr.* **2013**, *46* (2), 544–549. https://doi.org/10.1107/S0021889813003531.

(13) Stone, K. H.; Cosby, M. R.; Strange, N. A.; Thampy, V.; Walroth, R. C.; Troxel Jr, C. Remote and Automated High-Throughput Powder Diffraction Measurements Enabled by a Robotic Sample Changer at SSRL Beamline 2-1. *J. Appl. Crystallogr.* **2023**, *56* (5), 1480–1484. https://doi.org/10.1107/s1600576723007148.

(14) Coelho, A. A. *TOPAS* and *TOPAS-Academic*: An Optimization Program Integrating Computer Algebra and Crystallographic Objects Written in C++. *J. Appl. Crystallogr.* **2018**, *51* (1), 210–218. https://doi.org/10.1107/S1600576718000183.

(15) Filik, J.; Ashton, A. W.; Chang, P. C. Y.; Chater, P. A.; Day, S. J.; Drakopoulos, M.; Gerring, M. W.; Hart, M. L.; Magdysyuk, O. V.; Michalik, S.; Smith, A.; Tang, C. C.; Terrill, N. J.; Wharmby, M. T.; Wilhelm, H. Processing Two-Dimensional X-Ray Diffraction and Small-Angle Scattering Data in *DAWN 2*. *J. Appl. Crystallogr.* **2017**, *50* (3), 959–966. https://doi.org/10.1107/S1600576717004708.

(16) Juhás, P.; Davis, T.; Farrow, C. L.; Billinge, S. J. L. *PDFgetX3*: A Rapid and Highly Automatable Program for Processing Powder Diffraction Data into Total Scattering Pair Distribution Functions. *J. Appl. Crystallogr.* **2013**, *46* (2), 560–566. https://doi.org/10.1107/S0021889813005190.

(17) Yang, X.; Juhas, P.; Farrow, C. L.; Billinge, S. J. L. xPDFsuite: An End-to-End Software Solution for High Throughput Pair Distribution Function Transformation, Visualization and Analysis. arXiv **2014**. https://doi.org/10.48550/arXiv.1402.3163.

(18) Kim, C.-H.; Parkin, S.; Bharara, M.; Atwood, D. Linear Coordination of Hg(II) by Cysteamine. *Polyhedron* **2002**, *21* (2), 225–228. https://doi.org/10.1016/S0277-5387(01)00978-0.
(19) Kubelka, P.; Munk, F. Z. Ein Beitrag Zur Optik Der Farbanstriche. *Z. Tech. Phys.* **1931**, *12*, 593–601.
(20) Mooney, J.; Kambhampati, P. Get the Basics Right: Jacobian Conversion of Wavelength and Energy Scales for Quantitative Analysis of Emission Spectra. *J. Phys. Chem. Lett.* **2013**, *4* (19), 3316–3318. https://doi.org/10.1021/jz401508t.
(21) Pan, F.; Li, X.; Johnson, A. C.; Dhuey, S.; Saunders, A.; Hu, M.-X.; Dixon, J. P.; Dagli, S.; Lau, S.-C.; Weng, T.; Chen, C.-Y.; Zeng, J.-H.; Apte, R.; Heinz, T. F.; Liu, F.; Deng, Z.-L.; Dionne, J. A. Room-Temperature Valley-Selective Emission in Si-$MoSe_2$ Heterostructures Enabled by High-Quality-Factor Chiroptical Cavities. *Nat. Commun.* **2025**. https://doi.org/10.1038/s41467-025-66502-4.
(22) Greenspan, L. Humidity Fixed Points of Binary Saturated Aqueous Solutions. *J. Res. Natl. Bur. Stand. Sect. Phys. Chem.* **1977**, *81A* (1), 89. https://doi.org/10.6028/jres.081A.011.
(23) Campbell, B. J.; Stokes, H. T.; Tanner, D. E.; Hatch, D. M. *ISODISPLACE*: A Web-Based Tool for Exploring Structural Distortions. *J. Appl. Crystallogr.* **2006**, *39* (4), 607–614. https://doi.org/10.1107/S0021889806014075.
(24) Stokes, H. T.; Hatch, D. M.; Campbell, B. J. ISOCIF and ISODISTORT, ISOTROPY Software Suite, iso.byu.edu.
(25) Hohenberg, P.; Kohn, W. Inhomogeneous Electron Gas. *Phys. Rev.* **1964**, *136* (3B), B864–B871. https://doi.org/10.1103/PhysRev.136.B864.
(26) Kohn, W.; Sham, L. J. Self-Consistent Equations Including Exchange and Correlation Effects. *Phys. Rev.* **1965**, *140* (4A), A1133–A1138. https://doi.org/10.1103/PhysRev.140.A1133.
(27) Giannozzi, P.; Baroni, S.; Bonini, N.; Calandra, M.; Car, R.; Cavazzoni, C.; Ceresoli, D.; Chiarotti, G. L.; Cococcioni, M.; Dabo, I.; Dal Corso, A.; De Gironcoli, S.; Fabris, S.; Fratesi, G.; Gebauer, R.; Gerstmann, U.; Gougoussis, C.; Kokalj, A.; Lazzeri, M.; Martin-Samos, L.; Marzari, N.; Mauri, F.; Mazzarello, R.; Paolini, S.; Pasquarello, A.; Paulatto, L.; Sbraccia, C.; Scandolo, S.; Sclauzero, G.; Seitsonen, A. P.; Smogunov, A.; Umari, P.; Wentzcovitch, R. M. QUANTUM ESPRESSO: A Modular and Open-Source Software Project for Quantum Simulations of Materials. *J. Phys. Condens. Matter* **2009**, *21* (39), 395502. https://doi.org/10.1088/0953-8984/21/39/395502.
(28) Giannozzi, P.; Andreussi, O.; Brumme, T.; Bunau, O.; Buongiorno Nardelli, M.; Calandra, M.; Car, R.; Cavazzoni, C.; Ceresoli, D.; Cococcioni, M.; Colonna, N.; Carnimeo, I.; Dal Corso, A.; De Gironcoli, S.; Delugas, P.; DiStasio, R. A.; Ferretti, A.; Floris, A.; Fratesi, G.; Fugallo, G.; Gebauer, R.; Gerstmann, U.; Giustino, F.; Gorni, T.; Jia, J.; Kawamura, M.; Ko, H.-Y.; Kokalj, A.; Küçükbenli, E.; Lazzeri, M.; Marsili, M.; Marzari, N.; Mauri, F.; Nguyen, N. L.; Nguyen, H.-V.; Otero-de-la-Roza, A.; Paulatto, L.; Poncé, S.; Rocca, D.; Sabatini, R.; Santra, B.; Schlipf, M.; Seitsonen, A. P.; Smogunov, A.; Timrov, I.; Thonhauser, T.; Umari, P.; Vast, N.; Wu, X.; Baroni, S. Advanced Capabilities for Materials Modelling with Quantum ESPRESSO. *J. Phys. Condens. Matter* **2017**, *29* (46), 465901. https://doi.org/10.1088/1361-648X/aa8f79.
(29) Hamann, D. R. Optimized Norm-Conserving Vanderbilt Pseudopotentials. *Phys. Rev. B* **2013**, *88* (8), 085117. https://doi.org/10.1103/PhysRevB.88.085117.
(30) Perdew, J. P.; Burke, K.; Ernzerhof, M. Generalized Gradient Approximation Made Simple. *Phys. Rev. Lett.* **1996**, *77* (18), 3865–3868. https://doi.org/10.1103/PhysRevLett.77.3865.
(31) Van Setten, M. J.; Giantomassi, M.; Bousquet, E.; Verstraete, M. J.; Hamann, D. R.; Gonze, X.; Rignanese, G.-M. The PseudoDojo: Training and Grading a 85 Element Optimized Norm-Conserving Pseudopotential Table. *Comput. Phys. Commun.* **2018**, *226*, 39–54. https://doi.org/10.1016/j.cpc.2018.01.012.
(32) Lufaso, M. W.; Woodward, P. M. Jahn–Teller Distortions, Cation Ordering and Octahedral Tilting in Perovskites. *Acta Crystallogr. B* **2004**, *60* (1), 10–20. https://doi.org/10.1107/S0108768103026661.

(33) Robinson, K.; Gibbs, G. V.; Ribbe, P. H. Quadratic Elongation: A Quantitative Measure of Distortion in Coordination Polyhedra. *Science* **1971**, *172* (3983), 567–570. https://doi.org/10.1126/science.172.3983.567.
(34) Yao, R.; Lin, J.; Liu, K.; Xu, Y.; Xiao, B.; Zhao, J.; Guo, Z.; Liu, Q.; Yuan, W. Structure and Optical Properties of Sn-Based Halide Perovskites ($C_{10}H_{18}N_2$)SnX_4 (X = Cl, Br, I). *ACS Omega* **2024**, *9* (20), 22352–22359. https://doi.org/10.1021/acsomega.4c01835.
(35) Yamada, K.; Kuranaga, Y.; Ueda, K.; Goto, S.; Okuda, T.; Furukawa, Y. Phase Transition and Electric Conductivity of $ASnCl_3$ (A = Cs and CH_3NH_3). *Bull. Chem. Soc. Jpn.* **1998**, *71* (1), 127–134. https://doi.org/10.1246/bcsj.71.127.
(36) Wang, L.; Ou, T.; Wang, K.; Xiao, G.; Gao, C.; Zou, B. Pressure-Induced Structural Evolution, Optical and Electronic Transitions of Nontoxic Organometal Halide Perovskite-Based Methylammonium Tin Chloride. *Appl. Phys. Lett.* **2017**, *111* (23), 233901. https://doi.org/10.1063/1.5004186.
(37) van den Berg, J. M. The Crystal Structure of $SnCl_2$. *Acta Crystallogr.* **1961**, *14* (9), 1002–1003. https://doi.org/10.1107/S0365110X61002886.
(38) Islam, M. J.; Yuyama, K.; Takahashi, K.; Nakamura, T.; Konishi, K.; Biju, V. Mixed-Halide Perovskite Synthesis by Chemical Reaction and Crystal Nucleation under an Optical Potential. *NPG Asia Mater.* **2019**, *11* (1), 31. https://doi.org/10.1038/s41427-019-0131-0.
(39) Lumbreras, M.; Protas, J.; Jebbari, S.; Dirksen, G. J.; Schoonman, J. Structure and Ionic Conductivity of Mixed Lead Halides $PbCl_{2x}Br_{2(1-x)}$. II. *Solid State Ion.* **1986**, *20* (4), 295–304. https://doi.org/10.1016/0167-2738(86)90049-4.
(40) Kudelski, A.; Hill, W. Raman Study on the Structure of Cysteamine Monolayers on Silver. *Langmuir* **1999**, *15* (9), 3162–3168. https://doi.org/10.1021/la9811463.
(41) Aroyo, M. I.; Orobengoa, D.; De La Flor, G.; Tasci, E. S.; Perez-Mato, J. M.; Wondratschek, H. Brillouin-Zone Database on the *Bilbao Crystallographic Server*. *Acta Crystallogr. A* **2014**, *70* (2), 126–137. https://doi.org/10.1107/s205327331303091x.
(42) Kokalj, A. XCrySDen—a New Program for Displaying Crystalline Structures and Electron Densities. *J. Mol. Graph. Model.* **1999**, *17* (3–4), 176–179. https://doi.org/10.1016/S1093-3263(99)00028-5.
(43) Zhang, T.; Zhou, C.; Feng, X.; Dong, N.; Chen, H.; Chen, X.; Zhang, L.; Lin, J.; Wang, J. Regulation of the Luminescence Mechanism of Two-Dimensional Tin Halide Perovskites. *Nat. Commun.* **2022**, *13* (1), 60. https://doi.org/10.1038/s41467-021-27663-0.
(44) Fontana, P.; Schefer, J.; Pettit, D. Characterization of Sodium Chloride Crystals Grown in Microgravity. *J. Cryst. Growth* **2011**, *324* (1), 207–211. https://doi.org/10.1016/j.jcrysgro.2011.04.001.
(45) Reshchikov, M. A.; Korotkov, R. Y. Analysis of the Temperature and Excitation Intensity Dependencies of Photoluminescence in Undoped GaN Films. *Phys. Rev. B* **2001**, *64* (11), 115205. https://doi.org/10.1103/PhysRevB.64.115205.
(46) Dohner, E. R.; Jaffe, A.; Bradshaw, L. R.; Karunadasa, H. I. Intrinsic White-Light Emission from Layered Hybrid Perovskites. *J. Am. Chem. Soc.* **2014**, *136* (38), 13154–13157. https://doi.org/10.1021/ja507086b.
(47) Ju, D.; Dang, Y.; Zhu, Z.; Liu, H.; Chueh, C. C.; Li, X.; Wang, L.; Hu, X.; Jen, A. K. Y.; Tao, X. Tunable Band Gap and Long Carrier Recombination Lifetime of Stable Mixed $CH_3NH_3Pb_xSn_{1-x}Br_3$ Single Crystals. *Chem. Mater.* **2018**, *30* (5), 1556–1565. https://doi.org/10.1021/acs.chemmater.7b04565.
(48) Siekmann, J.; Ravishankar, S.; Kirchartz, T. Apparent Defect Densities in Halide Perovskite Thin Films and Single Crystals. *ACS Energy Lett.* **2021**, *6* (9), 3244–3251. https://doi.org/10.1021/acsenergylett.1c01449.
(49) Wu, T.; Liu, X.; Luo, X.; Lin, X.; Cui, D.; Wang, Y.; Segawa, H.; Zhang, Y.; Han, L. Lead-Free Tin Perovskite Solar Cells. *Joule* **2021**, *5* (4), 863–886. https://doi.org/10.1016/j.joule.2021.03.001.
